# Exploring the Self-Assembled Tacticity in Aurophilic Polymeric Arrangements of Diphosphanegold(I) Fluorothiolates

**DOI:** 10.3390/molecules24234422

**Published:** 2019-12-03

**Authors:** Guillermo Moreno-Alcántar, Laura Salazar, Guillermo Romo-Islas, Marcos Flores-Álamo, Hugo Torrens

**Affiliations:** 1 Facultad de Química, Universidad Nacional Autónoma de México, Mexico City 04510, Mexico; lauris.kawai@gmail.com (L.S.); memo_romo14@hotmail.com (G.R.-I.); mfa@unam.mx (M.F.-Á.); 2 Institut de Science et d’Ingénierie Supramoleculaires (ISIS), University of Strasbourg, 67083 Strasbourg, France

**Keywords:** gold, aurophilic, supramolecular chirality, tacticity

## Abstract

Despite the recurrence of aurophilic interactions in the solid-state structures of gold(I) compounds, its rational control, modulation, and application in the generation of functional supramolecular structures is an area that requires further development. The ligand effects over the aurophilic-based supramolecular structures need to be better understood. This paper presents the supramolecular structural diversity of a series of new 1,3-bis(diphenylphosphane)propane (dppp) gold(I) fluorinated thiolates with the general formula [Au_2_(SR_F_)_2_(μ-dppp)] (SR_F_ = SC_6_F_5_ (**1**); SC_6_HF_4_-4 (**2**); SC_6_H_3_(CF_3_)_2_-3,5 (**3**); SC_6_H_4_CF_3_-2 (**4**); SC_6_H_4_CF_3_-4 (**5**); SC_6_H_3_F_2_-3,4 (**6**); SC_6_H_3_F_2_-3,5 (**7**); SC_6_H_4_F-2 (**8**); SC_6_H_4_F-3 (**9**); SC_6_H_4_F-4 (**10**)). These compounds were synthesized and characterized, and six of their solid-state crystalline structures were determined using single-crystal X-ray diffraction. In the crystalline arrangement, they form aurophilic-bridged polymers. In these systems, the changes in the fluorination patterns of the thiolate ligands tune the aurophilic-induced self-assembly of the compounds causing tacticity and chiral differentiation of the monomers. This is an example of the use of ligand effects on the tune of the supramolecular association of gold complexes.

## 1. Introduction

Gold(I) coordination chemistry is intrinsically conjoined to supramolecular chemistry by the aurophilic interactions [1,2,3]. These counterintuitive interactions, which have attracted attention since the beginning of the twenty-first century, are still a subject of interest due to the potential applicability of supramolecular gold assemblies in the production of materials including, for example, luminescent materials, gelators, and liquid crystals [4,5,6,7]. In this quest, one of the main challenges is the rational control of the aurophilic contact and its supramolecular consequences [8,9,10,11,12].

In gold(I) linear compounds, aurophilic contacts form whenever the volumes of the ligands allow it [13,14,15]. Due to the high energy of the Au–Au contact, only the inclusion of synthons that promote strong competing interactions, such as hydrogen bonding motifs, could avoid gold–gold contacts [2,16]. If a truly rational use of aurophilicity as a supramolecular assembling tool could be achieved, the recurrence of the contact must be ensured and the available tools to control their directionality must be improved. Due to this lack in directionality, aurophilic-built architectures have a tendency to form diverse aggregates even if they have similar ligands [17,18,19,20,21,22]. As a consequence, the fine-tuning of the characteristics of the ligands become important for building supramolecular gold architectures. In the case of bisphosphane ligands, the length and flexibility of the bridging group fundamentally influence the type of supramolecular network that will form [1]. 

Previously, we have reported the importance of the fluorothiolate ligand when choosing the supramolecular packing motif in 1,2-bis(diphenylphosphane)ethane (dppe) gold(I) fluorothiolates [16]. Continuing with our research in this type of system, we synthesized and characterized a series of 10 new [Au_2_(SRF)_2_(μ-dppp)] derivatives containing fluorinated thiolates (SR_F_ = SC_6_F_5_ (**1**); SC_6_HF_4_-4 (**2**); SC_6_H_3_(CF_3_)_2_-3,5 (**3**); SC_6_H_4_(CF_3_)-2 (**4**); SC_6_H_4_(CF_3_)-4 (**5**); SC_6_H_3_F_2_-3,4 (**6**); SC_6_H_3_F_2_-3,5 (**7**); SC_6_H_4_F-2 (**8**); SC_6_H_4_F-3 (**9**); SC_6_H_4_F-4 (**10**)) and 1,3-bis(diphenylphosphano)propane (dppp). The single-crystal structure was determined for six of the obtained compounds. In contrast with our previous results—the use of dppe allows the formation of diverse crystalline arrangements—the addition of one extra carbon atom to the bridge using dppp seems to enhance the formation of polymeric chains, relegating the influence of the fluorothiolate to a second plane (i.e., the fluorinated thiolates participate in the folding of the lateral phenyl groups around the polymeric aurophilic metalorganic chain). The fluorinated moieties influence the kind of interactions between neighboring chains. This influence of the fluorinate groups is relevant in the supramolecular induction of particular arrangements, causing different kinds of tacticity in the polymeric chains and chiral differentiation of the initially achiral molecular units. Despite the growing attention on supramolecular tacticity and chirality [23,24] and the interesting features of aurophilic coordination polymers [25,26,27], to the extent of our knowledge, there is no previous examination of the tacticity in this kind of system.

## 2. Results

Via X-ray diffraction, we determined the structure of six out of the 10 synthesized compounds (Figures S1–S6). Table 1 lists bonding distances and angles around the gold atoms; all the bonding Au–P and Au–S distances remained within fixed ranges close to 2.26 and 2.30 Å, respectively. The distortions observed from the linearity expected for the P–Au–S moiety are mainly due to the formation of the Au–Au contacts; however, no correlation exists between the aurophilic distance and the Au–S/P bond distances, nor with the distortion angles, evidencing also a strong influence of packing effects. The recurrence of the Au–Au interactions in all the compounds is evidence of the intensity of the interaction despite the different electronic and steric properties of the used ligands.

All the obtained structures display linear polymeric arrangements in which the adjacent molecular units are linked by aurophilic contacts. Structures of **1** and **5** show syndiotactic polymers, whereas compounds **2**, **4**, **9**, and **10** crystallize forming polymeric isotactic arrangements. In all the compounds, the two gold atoms of each molecular unit are inequivalent but, in all cases, the obtained polymers are head-to-tail polymers with Au1–Au2 aurophilic interactions making all the gold to gold distances identical.

## 3. Discussion

### 3.1. Isotactic Polymers

**Figure 1:**
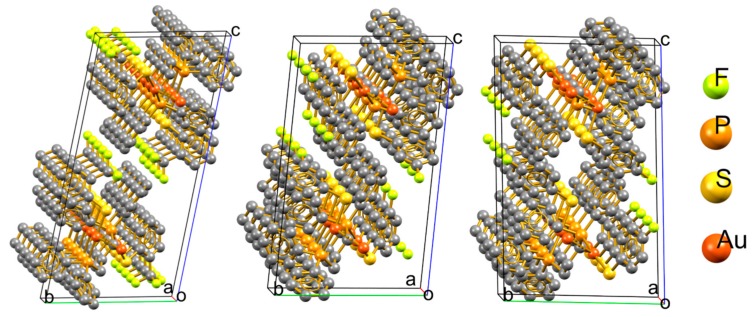
View of the unit cells in the polymeric aggregates of compounds **4**, **9**, and **10**.

Compounds **4**, **9**, and **10** crystallize in the triclinic P_−1_ spatial group; in the three cases, two molecular units are related by the inversion center per unit cell and the aurophilic polymers containing these units grow parallel to the *a* crystallographic axis in opposite directions from the other. Figure 1 shows the unit cells within an overview of the polymeric growth. All three arrangements are similar in terms of the polymer inside the backbone built by the aurophilic contacts and the phosphine bridges. The volumes of the unit cells (1971.3, 1770.8, and 1731.3 Å^3^ for **4**, **9**, and **10**, respectively) show that the packing is considerably looser in **4**.

**Figure 2:**
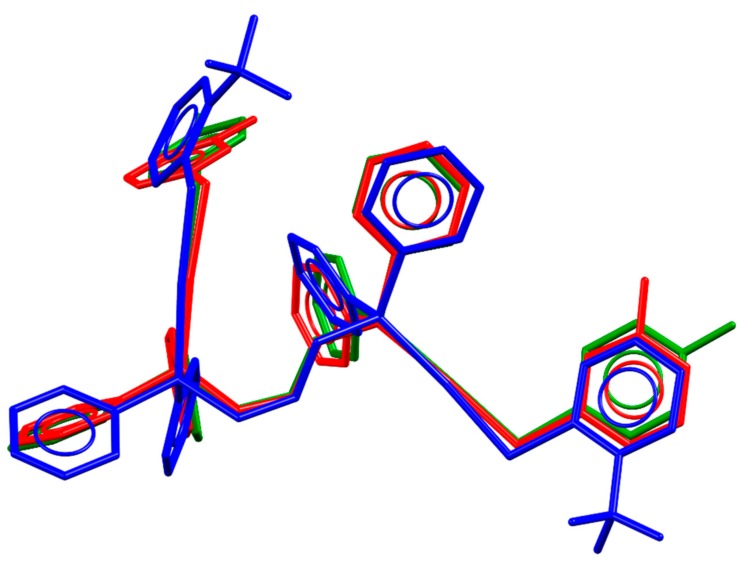
Overlapping molecular structures of compounds **4** (blue), **9** (red), and **10** (green), showing the similarities within this group of compounds.

In terms of the packing similarities, the molecular structures of the three compounds are also similar with few conformational differences in the phenyl rings of the ligands. The overlap of the molecular structure of these compounds is shown in Figure 2. The phosphine digold fragment remains almost unchanged through the compounds having the main differences in the thiolate and phosphine pendant phenyl groups. The biggest change is observed for compound **4** due to the difference in the volume of trifluoromethyl groups in comparison with fluorine or hydrogen, which also explains the decrease in the packing compactness observed in this compound.

**Figure 3:**
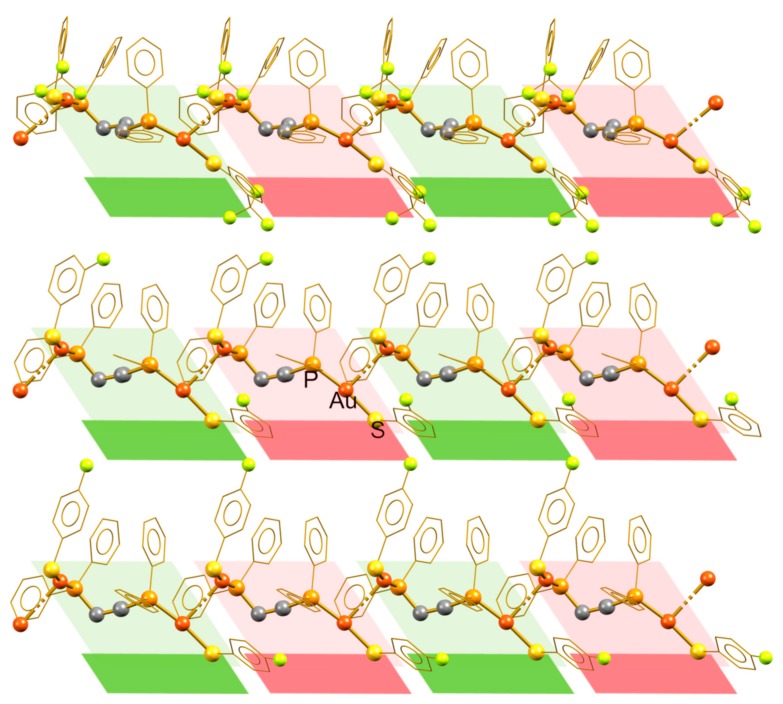
Isotactic aurophilic structures formed by compounds **4** (**top**), **9** (**middle**), and **10** (**bottom**), showing the structurally equivalent alternating molecular units.

The self-aggregation of the molecular units of these compounds through the formation of intermolecular aurophilic contacts yields the supramolecular linear polymers displayed in Figure 3. In the three cases, the polymeric aggregate can be generated by simple translation operations from the molecular unit, originating isotactic polymeric structures. Figure 3 distinguishes vicinal molecular units, by alternating green and red, to demonstrate that the conformation of the units remains along the polymeric chains. In these compounds, the supramolecular interactions holding together vicinal chains are not particularly strong. The analysis of the packing revealed some H···F contacts, which do not promote important changes in the morphology of the inner polymeric structure.

**Figure 4:**
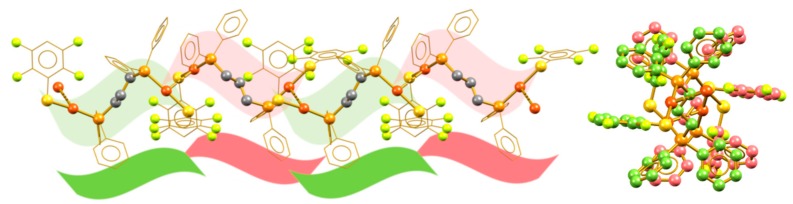
Isotactic aurophilic structures formed by compound **2** (**left**) and the lateral view of the polymeric chain showing the vicinal alternated monomers in red and green (**right**).

Compound **2** also forms isotactic polymeric chains in the crystalline packing, but, different from the other isotactic arrangements, the vicinal molecular units cannot be generated from simple translation operations, but are instead related by a second-order screw axis operation. Figure 4 shows the polymeric arrangement and the lateral view of the compound, displaying the existence of this relationship. The crystalline system of compound **2** is also different, showing a C_2/C_ monoclinic arrangement. The structural change in **2** is related to the existence of strong π-stacking interactions between neighboring chains (Figure S7). These interactions distort the molecular structure with respect to the conformation observed in the other compounds. Thus, the increase in the fluorination degree of compound **2** drives the change in the structural motif of the compound.

### 3.2. Syndiotactic Polymers 

**Figure 5:**
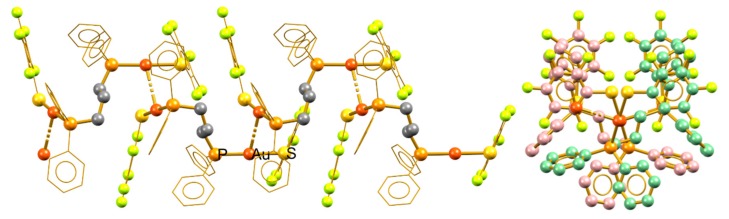
Syndiotactic aurophilic structures formed by compound **1**; the monomeric vicinal units are indicated in red and green.

Compounds **1** and **5** form syndiotactic aggregates in which vicinal units are not related to simple translation or rotation operations; instead, they show conformational differences. Figure 5 shows the polymeric aggregate in compound **1** and a visualization over the polymer growth direction. Unlike the previously examined compounds, the fluorinated phenyl rings exist in the same face of the polymeric chain as a result of the appearance of π_F_–π_F_ stacking interactions. The neighboring units in the rigidity of the crystal arrangement are enantiomers. Thus, the minimal expression of the polymeric chain consists of a *r* diad (i.e., a pair of enantiomeric molecular fragments) [28]. This relationship is due to the crystalline packing and no evidence exists of its prevalence in solution as the polymeric arrangements are broken by solvation. 

**Figure 6:**
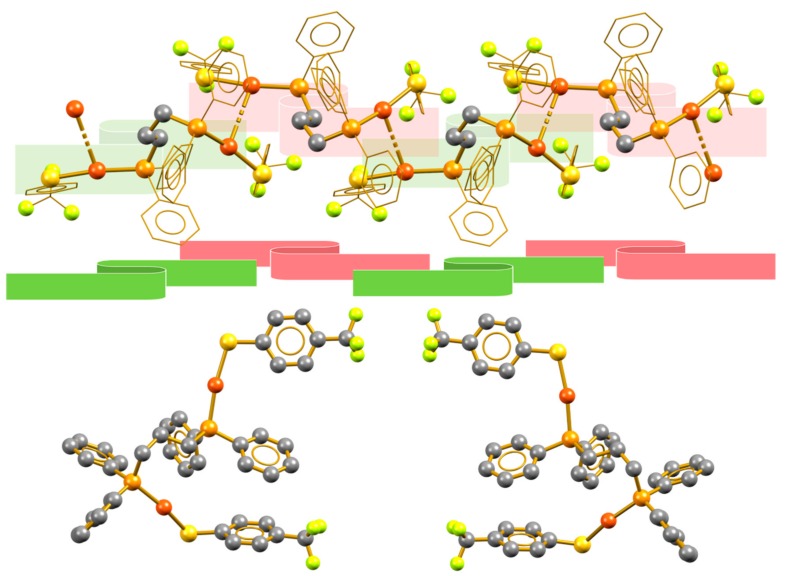
(**Top**) Syndiotactic aurophilic structures formed by compound **5**, the monomeric vicinal units are indicated in red and green. (**Bottom**) Conformation of the vicinal molecules showing their enantiomeric relation.

Similarly, compound **5** shows another kind of syndiotactic arrangement; in this case, the solvent molecules in the crystalline packing participate in directing the trifluoromethyl groups of the molecule to the same face, in which a layer of solvent molecules displays alternating Cl···H, Cl···F, and H···F interactions with the CF_3_ and hydrogen atoms in the phosphine phenyl rings (Figure S8). This facial differentiation, caused by the supramolecular interactions in the crystal, results in a loss of symmetry that yields to the formation of a syndiotactic polymer in which vicinal units of the polymer are stereoisomers (Figure 6). As in compound **1**, this relationship is inherent to the crystal packing structure and thus, in principle, should not be maintained in solution because aurophilic interactions are normally overcome by solvation.

## 4. Conclusions

We observed that the length of the bridge in the ligand 1,3-bis(diphenylphosphane)propane promotes the formation of supramolecular aurophilic coordination polymers, rather than other arrangements observed for different bridge lengths. In all the studied cases, the formation of the central Au–Au contacts seems to be the main force directing the crystalline packing.

Our results show that in this series of gold(I) supramolecular polymers, the different interactions promoted by the fluorinated moieties impacts the conformation of the molecular units, forming the dominant aurophilic coordination polymeric chains; by changing the fluorination pattern in the ancillary ligands, it is possible to induce structural properties such as tacticity and even chiral differentiation of the units. The possibility of controlling the prevalence of these properties is a major challenge in the building of self-assembled systems. This work demonstrated the feasibility of using the ligand-induced modulation in that pursuit.

## 5. Materials and Methods 

Fluorophenylthiols (HSR_F_), Pb(CH_3_COO)_2_, K[AuCl_4_], tetrahydrothiophene (tht), and 1,3-bis(diphenylphosphano) propane were purchased from Sigma-Aldrich and used without additional treatment. Solvents were obtained from JT Baker and used without previous treatment. 

IR spectra were obtained using a Perkin-Elmer Spectrum 400 (PerkinElmer, Inc., Waltham, MA, USA) in the range of 4000 to 400 cm^−1^ using attenuated total reflectance (ATR-FTIR). Elemental analysis was performed with a Thermo Scientific Flash 200 (Thermo Fisher Scientific., Waltham, MA, USA) at 950 °C. NMR spectra were recorded on a 9.4 T Varian VNMRS spectrometer (Varian, Inc., Palo Alto, CA, USA) in CDCl_3_. Chemical shifts are reported in ppm relative to internal TMS δ = 0 ppm (^1^H, ^13^C) and to external references of CFCl_3_ (for ^19^F) and H_3_PO_4_ (for ^31^P) at 0 ppm. Positive-ion fast atom bombardment mass spectrometry (FAB+MS) spectra were measured on an MStation JMS-700 (JEOL, Ltd., Tokyo, Japan). Crystals were grown by slow (1 week) evaporation of solutions of the compounds in chloroform.

### 5.1. Synthesis and Characterization

[AuCl(tht)] was synthesized by a modification of published methods [29,30]: A solution of 5.0 g (13.2 mmol) of K[AuCl_4_] in a mixture of 25 mL of water and 5 mL of ethanol were mixed with 2.5 mL (2.5 g, 28.35 mmol) of tetrahydrothiophene in a 100 mL round bottom flask (Caution: tht is highly odorous and volatile, thus the procedure must be conducted in a fume hood; the materials can be washed in a NaClO solution to mitigate the odor). The mixture was stirred for 1 h at room temperature and the product appeared as a white precipitate. The precipitate was filtered and washed 2× with 25 mL ice-cold ethanol and 3× with 25 mL hexane. 

[Au_2_Cl_2_(μ-dppp)] was synthesized according to previous reports [31]: We added 1.5 g (3.6 mmol) of solid dppp to a suspension of 2 g (6.2 mmol) [AuCl(tht)] in 50 mL of a 1:1 mixture of CH_2_Cl_2_ and acetone. After 2 h stirring at room temperature, a clear transparent solution was obtained. The solution was concentrated by reduced pressure evaporation, and when the total volume was about 5 mL, an excess of hexane (ca. 50 mL) was added causing the precipitation of the [Au_2_Cl_2_(dppp)] as a white powder. 

Pb(SR_F_)_2_: All lead thiolates were prepared by modification of previously published methods [32,33,34,35,36]: To a solution of Pb(CH_3_COO)_2_ (5.2 mmol) in 100 mL water, thiol (HSR_F_) (10.0 mmol) dissolved in about 10 mL of ethanol was added under vigorous stirring at room temperature. A white or yellow precipitate was rapidly formed. The solid was filtrated and washed 3× with 50 mL methanol and 3× with 25 mL hexane. Caution: Lead derivatives are extremely toxic and must be handled following the proper security procedures. Thiols and thiolates are odorous; consequently, all procedures must be completed in a fume hood. IR spectra of the lead thiolates are available in the SI.

All 10 compounds were prepared in a similar manner so only the synthesis of compound **1** is described in detail.

[Au_2_(SC_6_F_5_)_2_(μ-dppp)] (**1**). A solution of the precursor [Au_2_Cl_2_(dppp)] (210.0 mg; 0.24 mmol) in 10 mL CH_2_Cl_2_ was mixed under stirring at room temperature with a solution (or suspension for most of the less-fluorinated lead thiolates) of 170.0 mg (0.24 mmol) Pb(SC_6_F_5_)_2_ in 10 mL acetone. After 3 h of stirring, we obtained a clear solution. The solvent was evaporated until a volume of about 3 mL and then 20 mL of hexane was added to promote the precipitation of the product as a white powder. Yield: 75%; mp 193–195 °C; anal. C 39.2, H 1.8, S 4.8%, calcd for C_39_H_26_Au_2_F_10_S_2_P_2_, C 38.9, H 2.2 S 5.3%; IR (ATR) ν_max_ 3062, 2901, 1503, 1472, 967 cm^−1^; ^1^H-NMR (CDCl_3_, 400 MHz) δ 7.67–7.61 (8H, m), 7.53–7.42 (12H, m,), 2.83 (4H, dt, *J* = 10.5, 7.2 Hz), 2.03–1.88 (2H, m); ^31^P-NMR (CDCl_3_, 162 MHz) δ 27.94 ppm, ^19^F-NMR (CDCl_3_, 376.5 MHz) δ1 −35.32 (2F, m),1 −65.24 (1F, m),1 −66.80 (2F, m) ppm; FAB+ *m/z* 1401 [MAu]+ (20), 1005 [C_33_H_26_Au_2_F_5_P_2_S]^+^ (100).

[Au_2_(SC_6_HF_4_-4)_2_(μ-dppp)] (**2**). White powder. Yield: 76.5%. mp 171–173 °C; anal. C 40.3, H 2.1, S 5.4%, calcd for C_39_H_28_Au_2_F_8_S_2_P_2_, C 40.1, H 2.4, S 5.5%; IR (ATR) ν_max_ 3079, 2861, 1625, 1424, 886 cm^−1^; ^1^H-NMR (CDCl_3_, 400 MHz) δ 7.68–7.62 (8H, m), 7.52–7.41 (12H, m,), 6.68 (2H, tt, *J* = 9.8, 7.3 Hz), 2.83 (4H, dt, *J* = 10.5, 7.3 Hz), 1.97 (2H, tp, *J* = 14.7, 7.3 Hz); ^31^P-NMR (CDCl_3_, 162 MHz) δ 28.39 ppm, ^19^F-NMR (CDCl_3_, 376.5 MHz) δ1 −35.41 (2F, m),1 −43.34 (2F, m) ppm; FAB+ *m/z* 1365 [MAu]+ (15), 987 [C_33_H_27_Au_2_F_4_P_2_S]^+^ (100).

[Au_2_(SC_6_H_3_(CF)_2_-3,5)_2_(μ-dppp)] (**3**). White powder. Yield: 66%. mp 125–128 °C; anal. C 39.5, H 2.2, S 5.1%, calcd for C_43_H_32_Au_2_F_12_S_2_P_2_, C 39.8, H 2.5, S 4.9%; IR (ATR) ν_max_ 2921, 2855, 1591, 1348, 1274, 1109 cm^−1^; ^1^H-NMR (CDCl_3_, 400 MHz) δ 7.94 (4H, br, s), 7.69–7.63 (8H, m), 7.53–7.38 (14H, m), 2.88 (4H, dt, *J* = 10.4, 7.0 Hz), 1.97 (2H, tp, *J* = 16.3, 7.1 Hz); ^31^P-NMR (CDCl_3_, 162 MHz) δ 28.21 ppm, ^19^F-NMR (CDCl_3_, 376.5 MHz) δ6 −5.79 (12F, s) ppm; FAB+ *m/z* 1493 [MAu]+ (15), 1051 [C_35_H_29_Au_2_F_6_P_2_S]^+^ (100).

[Au_2_(SC_6_H_4_CF_3_-2)_2_(μ-dppp)] (**4**). White powder. Yield: 87%. mp 121–124 °C; anal. C 42.4, H 2.8, S 5.2%, calcd for C_41_H_34_Au_2_F_6_S_2_P_2_, C 42.4, H 2.9, S 5.5%; IR (ATR) ν_max_ 2921, 2859, 1436, 1309, 1100, 1027 cm^−1^; ^1^H-NMR (CDCl_3_, 400 MHz) δ 7.68–7.58 (8H, m), 7.52–7.48 (12H, m), 6.96–6.85 (8H, m), 2.82 (4H, dt, *J* = 10.5, 7.3 Hz), 1.96 (2H, tp, *J* = 14.8, 7.2 Hz); ^31^P-NMR (CDCl_3_, 162 MHz) δ 29.28 ppm, ^19^F-NMR (CDCl_3_, 376.5 MHz) δ −64.59 (6F, s) ppm; FAB+ *m/z* 1357 [MAu]+ (25), 983 [C_34_H_30_Au_2_F_3_P_2_S]^+^ (100).

[Au_2_(SC_6_H_4_CF_3_-4)_2_(μ-dppp)] (**5**). White powder. Yield: 79%. mp 158–160 °C; anal. C 42.6, H 2.7, S 5.1%, calcd for C_41_H_34_Au_2_F_6_S_2_P_2_, C 42.4, H 2.9, S 5.5%; IR (ATR) ν_max_ 2906, 2862, 1599, 1326, 1088 cm–1; ^1^H-NMR (CDCl_3_, 400 MHz) δ 7.68–7.57 (12H, m), 7.51–7.39 (12H, m), 7.24 (4H, d), 2.83 (4H, dt, *J* = 10.4, 7.2 Hz), 1.97 (2H, tp, *J* = 14.6, 7.2 Hz); ^31^P-NMR (CDCl_3_, 162 MHz) δ 28.64 ppm, ^19^F-NMR (CDCl_3_, 376.5 MHz) δ6 −4.73 (6F, s) ppm; FAB+ *m/z* 1357 [MAu]+ (45), 983 [C_34_H_30_Au_2_F_3_P_2_S]^+^ (100).

[Au_2_(SC_6_H_3_F_2_-3,4)_2_(μ-dppp)] (**6**). White powder. Yield: 92%. mp 174–176 °C; anal. C 42.5, H 2.4, S 5.6%, calcd for C_39_H_32_Au_2_F_4_S_2_P_2_, C 42.7, H 2.9, S 5.8%; IR (ATR) ν_max_ 2958, 2855, 1492, 1268, 1105 cm^−1^; ^1^H-NMR (CDCl_3_, 400 MHz) δ 7.67–7.60 (8H, m), 7.53–7.41 (12H, m,), 7.33–7.27 (2H, m), 7.20–7.15 (2H, m), 6.83 (2H, dt, *J* = 10.5, 7.3 Hz), 2.84 (4H, dt, *J* = 10.5, 7.2 Hz), 2.02–1.85 (2H, m); ^31^P-NMR (CDCl_3_, 162 MHz) δ 28.06 ppm, ^19^F-NMR (CDCl_3_, 376.5 MHz) δ1 −41.24 (2F, m), −147.95 (2F, m) ppm; FAB+ *m/z* 1293 [MAu]+ (20), 951 [C_33_H_29_Au_2_F_2_P_2_S]^+^ (100).

[Au_2_(SC_6_H_3_F_2_-3,5)_2_(μ-dppp)] (**7**). White powder. Yield: 92%. mp 141–143 °C; anal. C 43.0, H 2.6, S 5.5%, calcd for C_39_H_32_Au_2_F_4_S_2_P_2_, C 42.7, H 2.9, S 5.8%; IR (ATR) ν_max_ 3059, 2902, 1575, 1435, 978 cm^−1^; ^1^H-NMR (CDCl_3_, 400 MHz) δ 7.68–7.62 (8H, m), 7.54–7.41 (12H, m,), 7.11–6.97 (4H, m), 6.43 (2H, tt, *J* = 9.1, 2.3 Hz), 2.85 (4H, dt, *J* = 10.5, 7.2 Hz), 1.96 (2H, tp, *J* = 14.7, 7.3 Hz); ^31^P-NMR (CDCl_3_, 162 MHz) δ 26.39 ppm, ^19^F-NMR (CDCl_3_, 376.5 MHz) δ1 −10.63 (4F, s) ppm; FAB+ *m/z* 1293 [MAu]+ (10), 951 [C_33_H_29_Au_2_F_2_P_2_S]^+^ (100).

[Au_2_(SC_6_H_4_F-2)_2_(μ-dppp)] (**8**). White powder. Yield: 67%. mp 138–140 °C; anal. C 44.5, H 3.1, S 6.2%, calcd for C_39_H_34_Au_2_F_2_S_2_P_2_, C 44.2, H 3.2, S 6.0%; IR (ATR) ν_max_ 3055, 2928, 1464, 1435, 1102 cm^−1^; ^1^H-NMR (CDCl_3_, 400 MHz) δ 7.70–7.58 (8H, m), 7.52–7.38 (12H, m), 6.96–6.84 (8H, m), 2.82 (4H, dt, *J* = 10.5, 7.3 Hz), 1.96 (2H, tp, *J* = 14.8. 7.3 Hz); ^31^P-NMR (CDCl_3_, 162 MHz) δ 27.95 ppm, ^19^F-NMR (CDCl_3_, 376.5 MHz) δ1 −07.70 (2F, m) ppm; FAB+ *m/z* 1257 [MAu]+ (30), 933 [C_33_H_30_Au_2_FP_2_S]^+^ (100).

[Au_2_(SC_6_H_4_F-3)_2_(μ-dppp)] (**9**). White powder. Yield: 80%. mp 168–170 °C; anal. C 44.1, H 3.0, S 5.7%, calcd for C_39_H_34_Au_2_F_2_S_2_P_2_, C 44.2, H 3.2, S 6.0%; IR (ATR) νmax 2926, 2871, 1568, 1462, 1104 cm^−1^; ^1^H-NMR (CDCl_3_, 400 MHz) δ 7.68–7.59 (8H, m), 7.51–7.37 (12H, m), 7.33–7.23 (4H, m), 7.01 (2H, td, *J* = 8.0, 6.3 Hz), 6.67 (2H, ttd, *J* = 8.3, 2.5, 1.0 Hz), 2.84 (4H, dt, *J* = 10.5, 7.3 Hz), 1.96 (2H, tp, *J* = 14.8, 7.2 Hz); ^31^P-NMR (CDCl_3_, 162 MHz) δ 28.56 ppm, ^19^F-NMR (CDCl_3_, 376.5 MHz) δ −116.90 (2F, s) ppm; FAB+ *m/z* 1257 [MAu]+ (23), 933 [C_33_H_30_Au_2_FP_2_S]^+^ (100).

[Au_2_(SC_6_H_4_F-4)_2_(μ-dppp)] (**10**). White powder. Yield: 80%. mp 178–180 °C; anal. C 44.3, H 3.4, S 5.6%, calcd for C_39_H_34_Au_2_F_2_S_2_P_2_, C 44.2, H 3.2, S 6.0%; IR (ATR) ν_max_ 2905, 2861, 1598, 1327, 1089 cm^−1^; ^1^H-NMR (CDCl_3_, 400 MHz) δ 7.64-7.59 (8H, m), 7.49–7.36 (16H, m), 6.77–6.71 (4H, m), 2.81 (4H, dt, *J* = 10.6, 7.4 Hz), 1.93 (2H, tp, *J* = 14.9, 7.5 Hz); ^31^P-NMR (CDCl_3_, 162 MHz) δ 32.11 ppm, ^19^F-NMR (CDCl_3_, 376.5 MHz) δ1 −20.62 (2F, s) ppm; FAB+ *m/z* 1257 [MAu]+ (35), 933 [C_33_H_30_Au_2_FP_2_S]^+^ (100).

### 5.2. Crystal Structure Determination 

A suitable single crystal of compounds **1**, **2**, **4**, **5**, **9**, and **10** were mounted on a glass fiber and crystallographic data were collected with an Oxford Diffraction Gemini “A” diffractometer with a Charge Coupled Device (CCD)area detector with monochromator of graphite for λ_MoKα_ = 0.71073 Å. CrysAlisPro and CrysAlis RED software packages were used for data collection and integration [37]. The double pass scanning method was used to exclude any noise. The collected frames were integrated using an orientation matrix determined from the narrow frame scans. Final cell constants were determined by global refinement; collected data were corrected for absorbance using analytical numeric absorption correction, using a multifaceted crystal model based on expressions upon the Laue symmetry with equivalent reflections [38]. Structures solutions and refinement were conducted with the SHELXS-2014 [39] and SHELXL-2014 [40] packages. WinGX v2018.3 [41] software was used to prepare material for publication. Full-matrix least-squares were refined by minimizing (*Fo^2^ − Fc^2^*)^2^. All non-hydrogen atoms were refined anisotropically. H atoms attached to C atoms were placed in geometrically idealized positions and refined as riding on their parent atoms, with C–H = 0.95–1.00 Å and *U_iso_*(H) = 1.2*U_eq_*(C) for aromatic, methylene, and methine groups. Crystallographic data for all complexes are presented in Tables S1–S18. The crystallographic data for the structures reported in this paper were deposited with the Cambridge Crystallographic Data Centre (CCDC) as supplementary publication no. CCDC 1957797–1957802. Copies of the data can be obtained free of charge on application to CCDC, 12 Union Road, Cambridge, CB21EZ, U.K. (fax: (+44) 1223-336-033, e-mail: deposit@ccdc.cam.ac.uk).

## Figures and Tables

**Table 1:** Selected angles (θ) and distances (d) in the reported compounds.

Compound	d_Au–Au_ (Å)	d_Au–P_ (Å)		d_Au–S_ (Å)		θ_P–Au–S_ (°)	
**1**	3.0924 (7)	2.254 (2)	2.263 (1)	2.308 (2)	2.309 (1)	173.59 (7)	175.72 (7)
**2**	3.0288 (6)	2.257 (1)	2.259 (1)	2.308 (1)	2.313 (1)	178.40 (4)	173.63 (4)
**4**	3.2276 (3)	2.258 (1)	2.260 (1)	2.299 (1)	2.303 (1)	175.75 (4)	169.04 (4)
**5**	3.2071 (5)	2.259 (1)	2.272 (2)	2.302 (1)	2.301 (2)	169.93 (5)	165.83 (5)
**9**	3.1475 (5)	2.262 (3)	2.272 (3)	2.309 (3)	2.315 (3)	167.0 (1)	171.5 (1)
**10**	3.1325 (3)	2.259 (1)	2.271 (1)	2.305 (1)	2.316 (1)	169.88 (4)	172.02 (4)

## Supplementary Materials

The following are available online, supplementary figures, additional characterization information and crystallographic information.
