# Supplementary material for: Exploring the Self-Assembled Tacticity in Aurophilic Polymeric Arrangements of Diphosphanegold(I) Fluorothiolates
Source: Molecules. 2019 Dec 3;24(23):4422. doi: 10.3390/molecules24234422 (PMC6930485; doi:10.3390/molecules24234422)
Supplement: Supplementary file 1 [file molecules-24-04422-s001.pdf]

Supplementary Information

Exploring the self-assembled tacticity in aurophilic polymeric arrangements of diphosphanegold(I) fluorothiolates

Guillermo Moreno-Alcántar [1,2]*, Laura Salazar [2], Guillermo Romo-Islas [2], Marcos Flores-Álamo [2] and Hugo Torrens [2]*

[1] Institut de Science et d'Ingénierie Supramoleculaires (ISIS), University of Strasbourg;
[2] Facultad de Química, Universidad Nacional Autónoma de México.
* Correspondence: morenoalcantar@unistra.fr, lgma@comunidad.unam.mx; Tel.: +52-55-5622-3724

Contents

Supplementary figures 2
NMR Spectra 6
Crystallographic tables 21
Lead Thiolates ($Pb(SR_F)_2$) IR Spectra 59

Supplementary figures

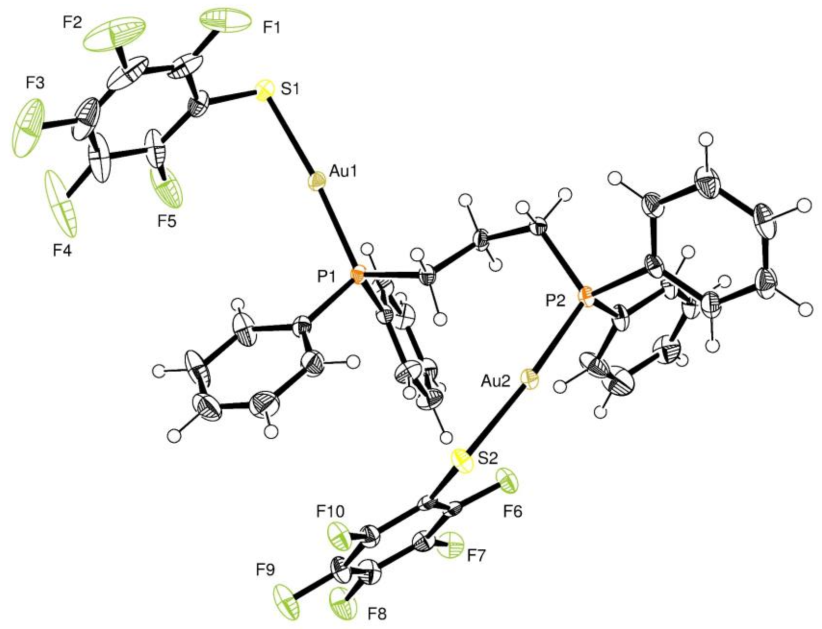

Figure S1 ORTEP drawing of the asymmetric unit for compound 1 with the thermal ellipsoids drawn at the 50% of probability.

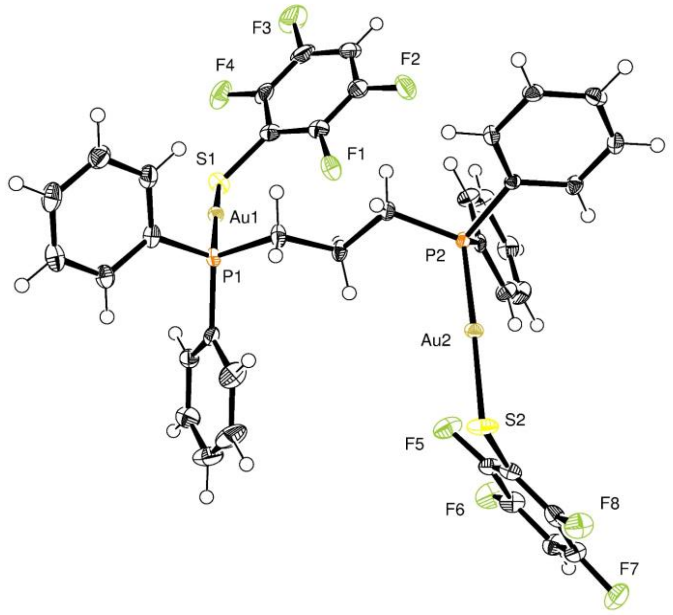

Figure S2 ORTEP drawing of the asymmetric unit for compound 2 with the thermal ellipsoids drawn at the 50% of probability.

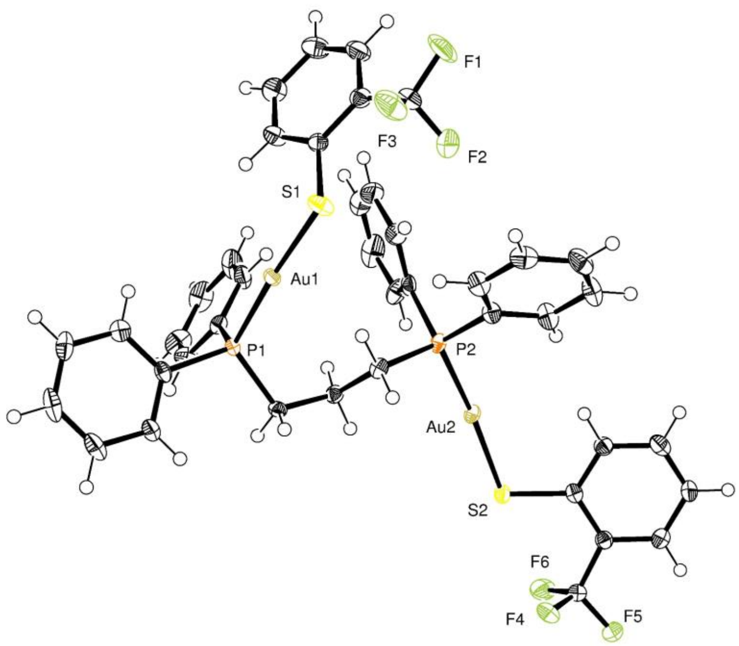

Figure S3 ORTEP drawing of the asymmetric unit for compound 4 with the thermal ellipsoids drawn at the 50% of probability.

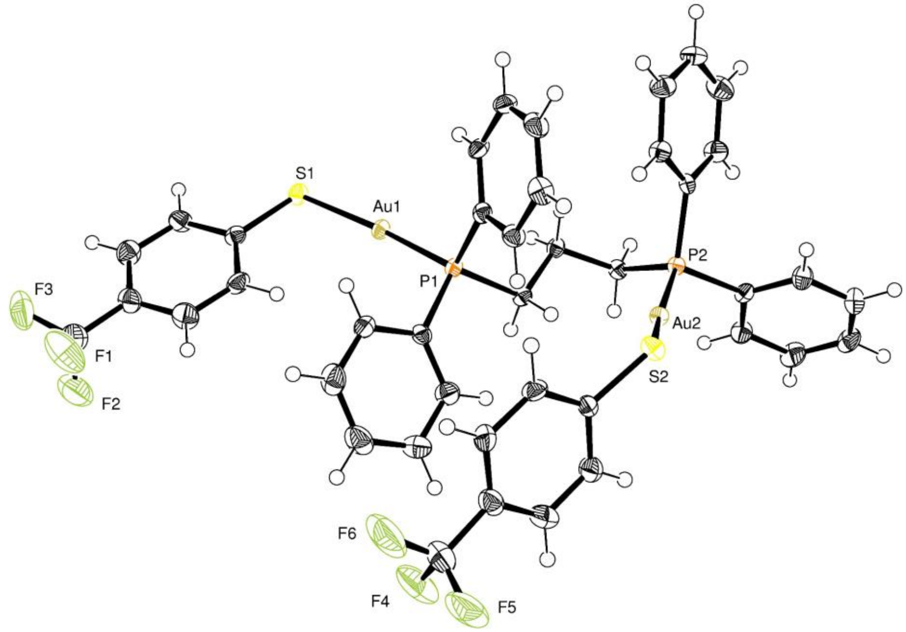

Figure S4. ORTEP drawing of the asymmetric unit for compound 5 with the thermal ellipsoids drawn at the 50% of probability. Chloroform molecules solvate are omitted for clarity.

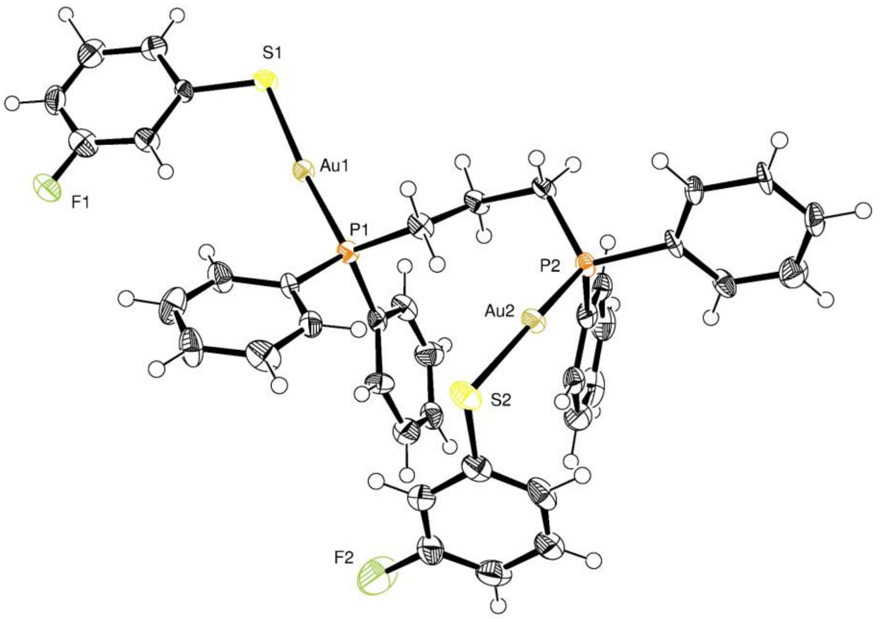

Figure S5 ORTEP drawing of the asymmetric unit for 9 with the thermal ellipsoids drawn at the 50% of probability.

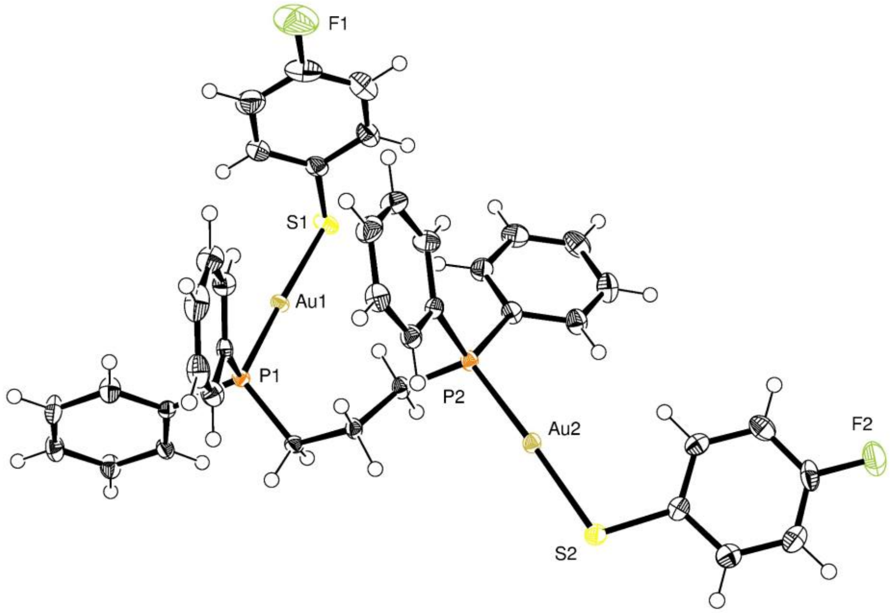

Figure S6 ORTEP drawing of the asymmetric unit for compound 10 with the thermal ellipsoids drawn at the 50% of probability.

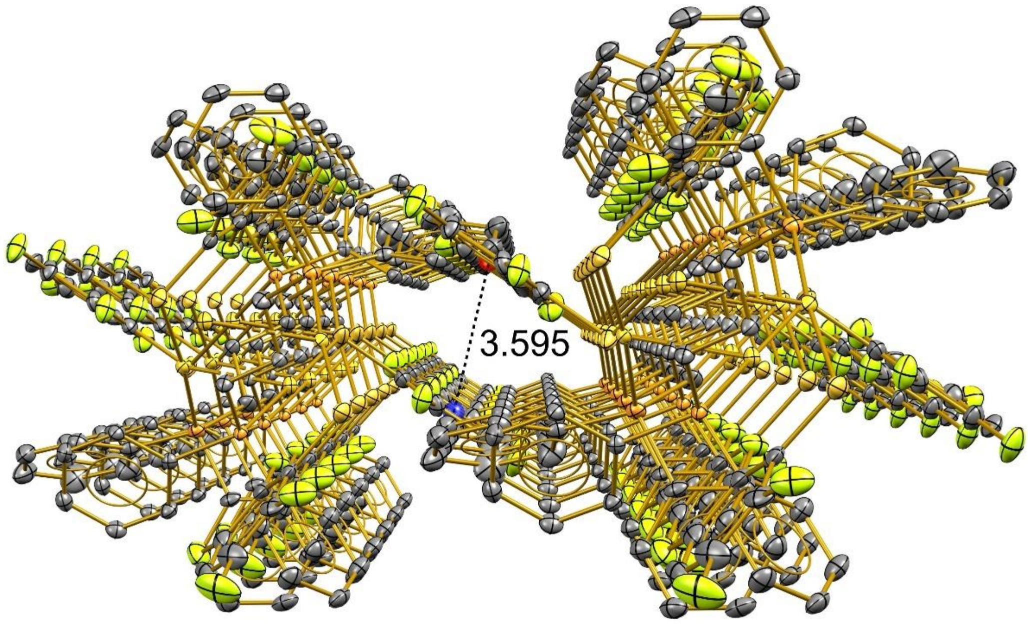

Figure S7 Lateral view over the b crystallographic axis of compound 2 showing two vicinal strands interacting by the stacking of fluorinated phenyl rings showing the centroid to centroid distance.

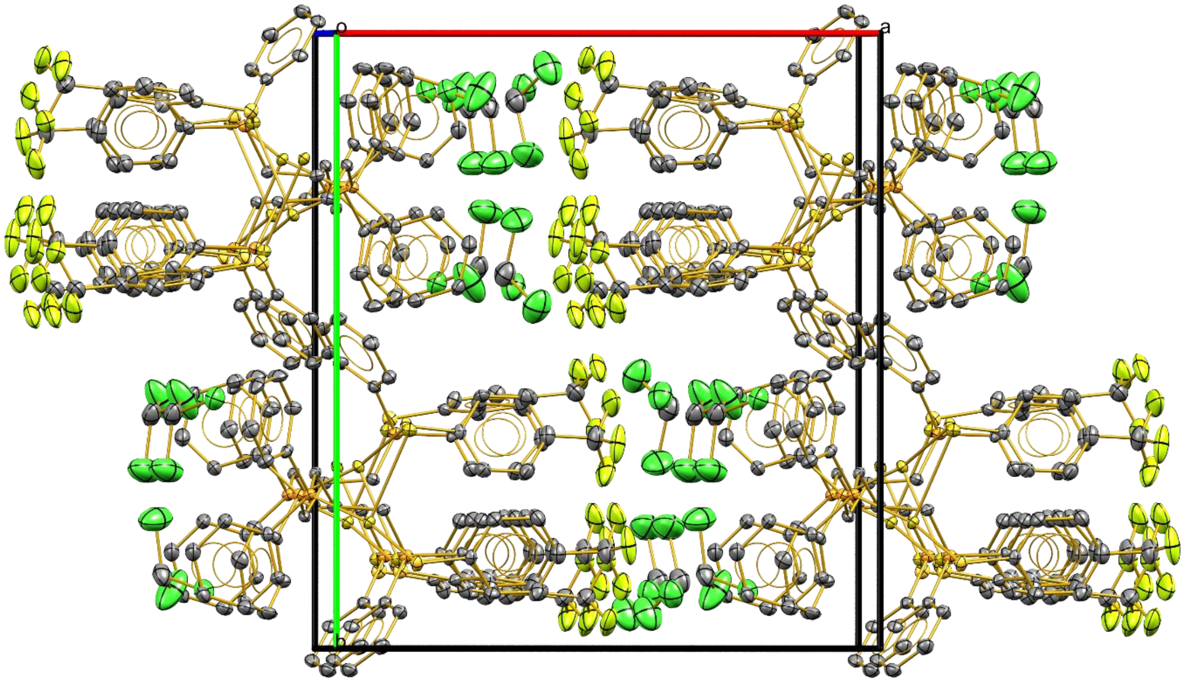

Figure S8 View of the crystalline packing of compound 5 showing the inclusion of solvent molecules that direct the CF3 groups.

NMR Spectra

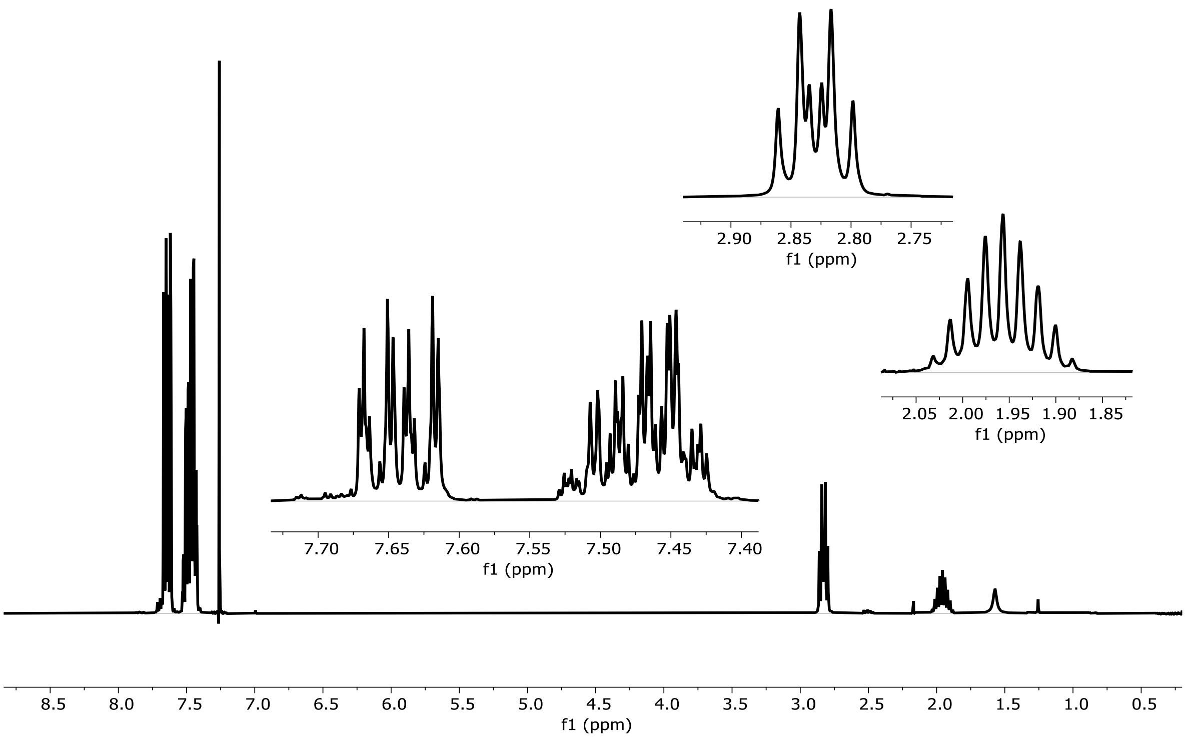

*Figure S9 Detail of the ^{1}H-NMR spectra of compound **1***

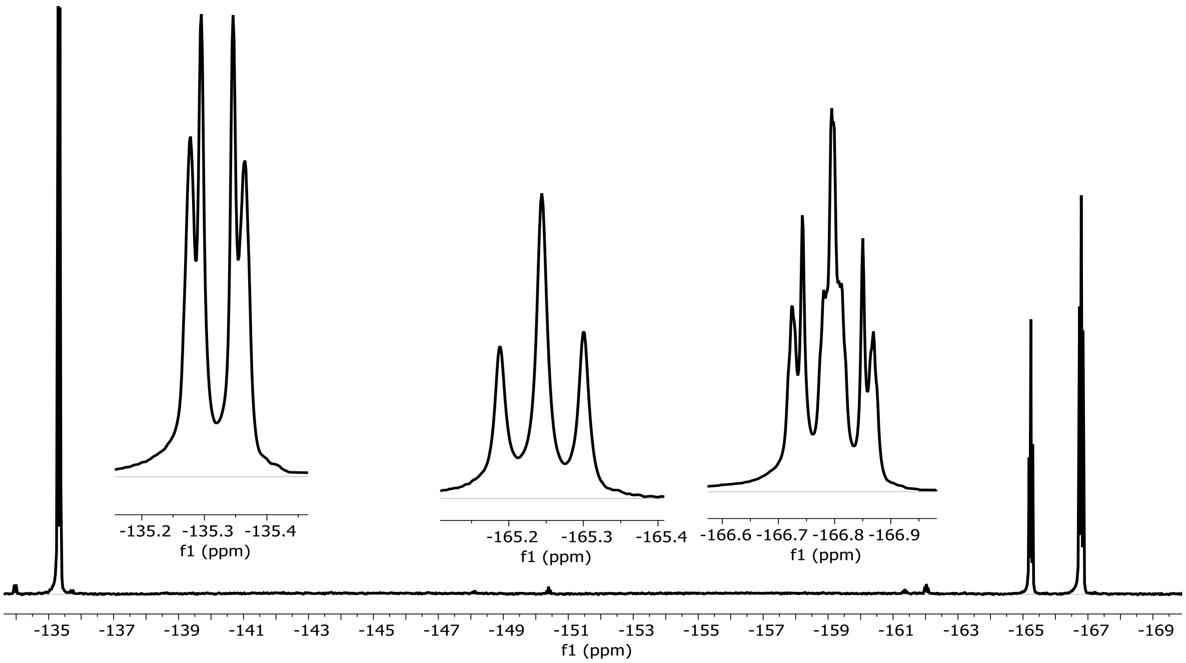

*Figure S10 Detail of the ^{19}F-NMR spectra of compound **1***

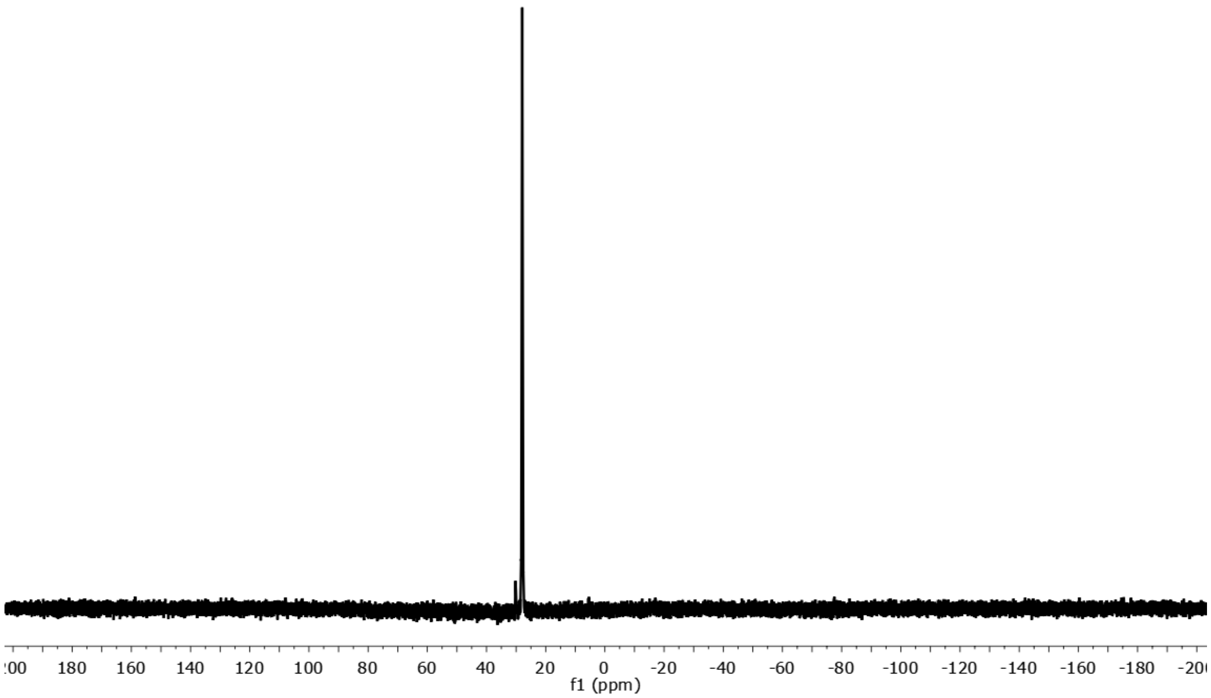

*Figure S11 Detail of the ^{31}P-NMR spectra of compound **1***

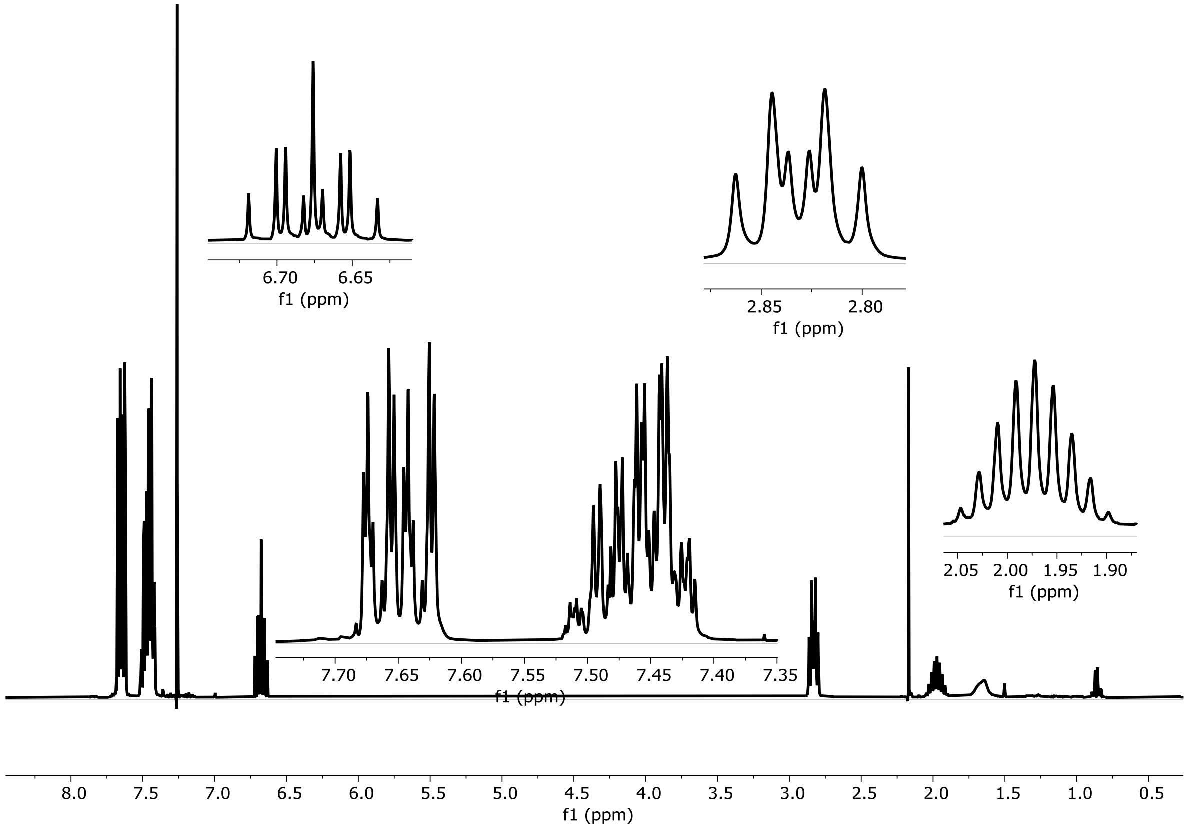

Figure S12 Detail of the ^{1}H-NMR spectra of compound **2**

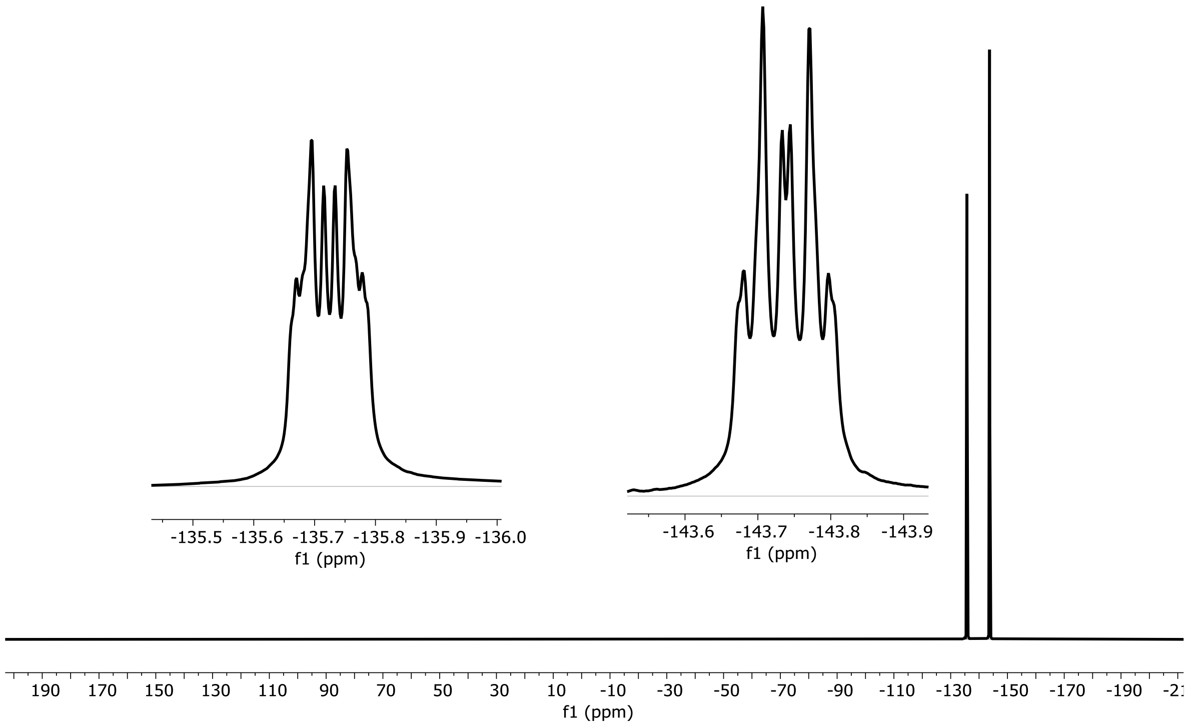

Figure S13 Detail of the ^{19}F-NMR spectra of compound 2

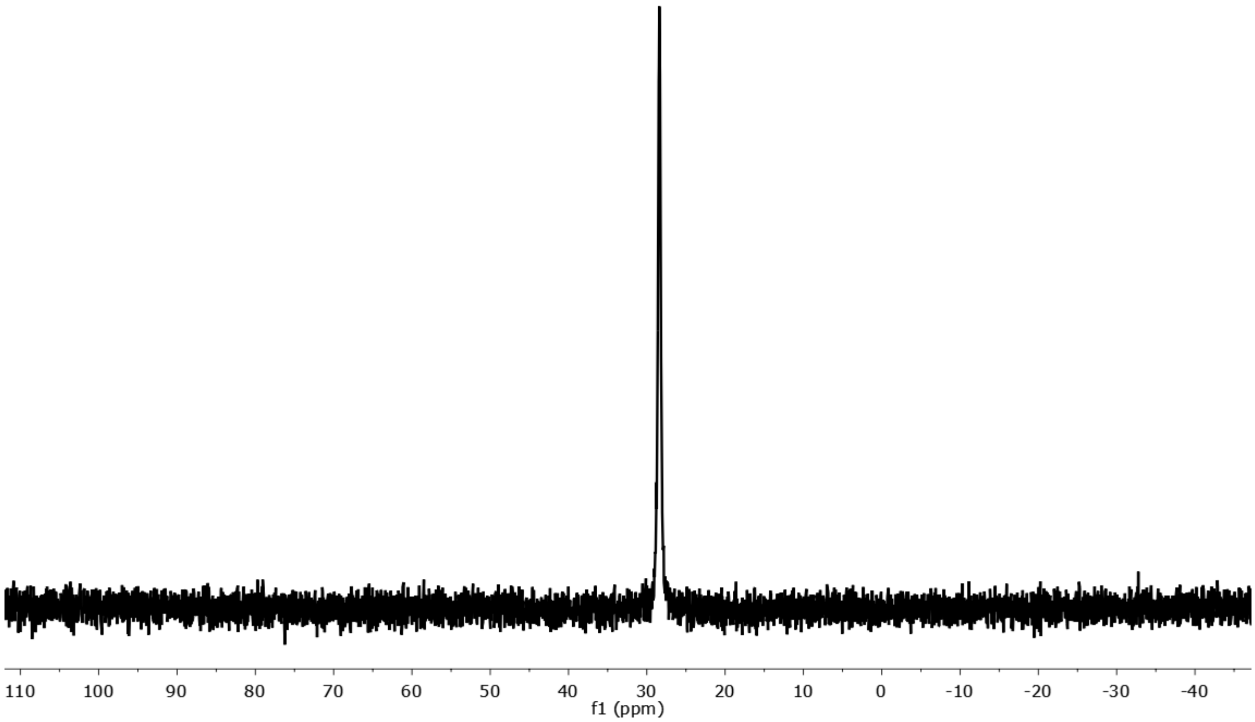

Figure S14 Detail of the ^{31}P-NMR spectra of compound 2

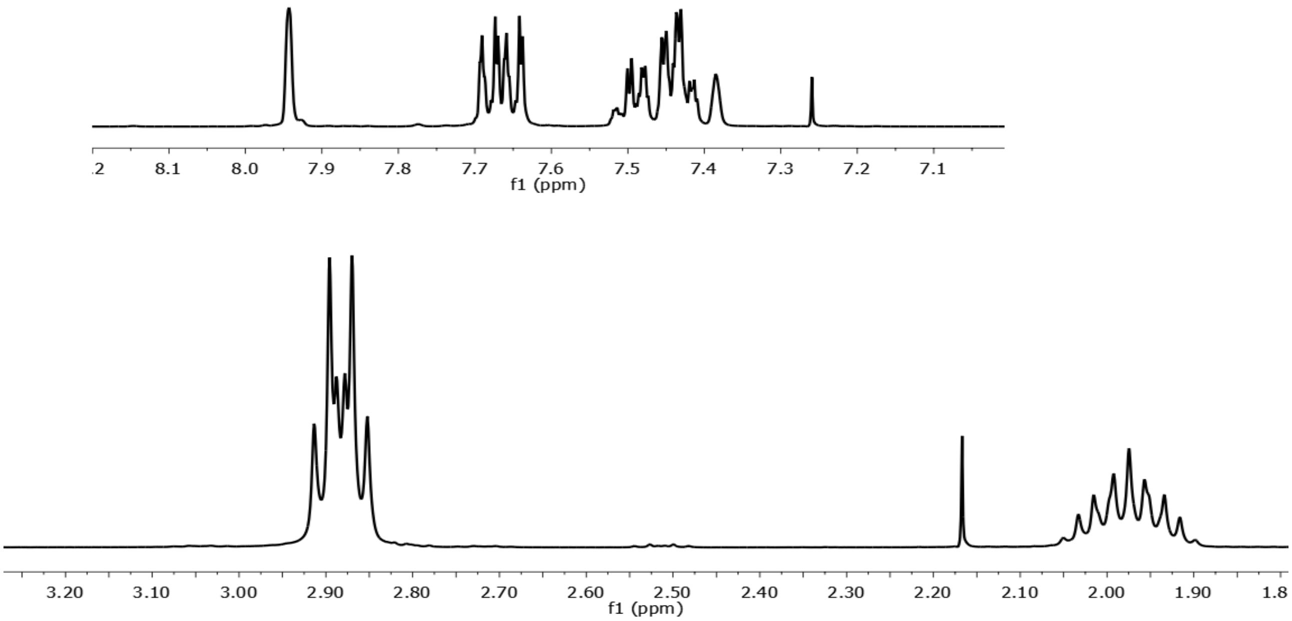

*Figure S15 Detail of the ¹H-NMR spectra of compound **3***

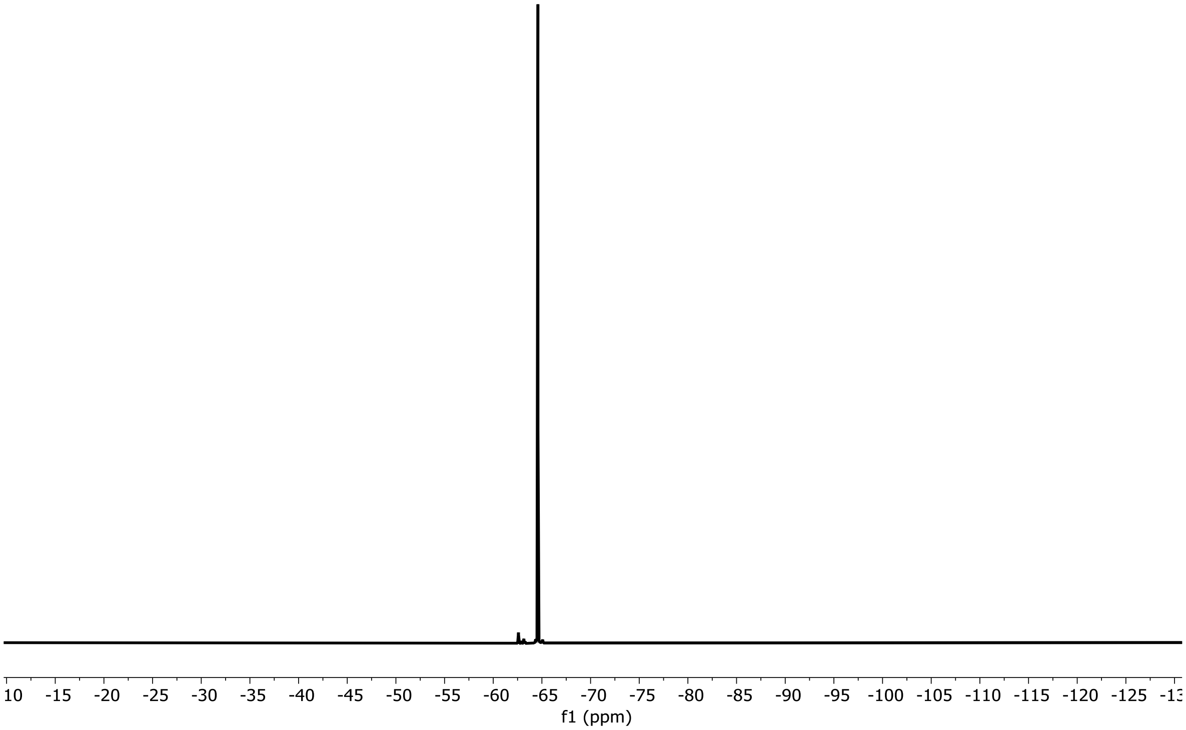

Figure S16 Detail of the ¹⁹F-NMR spectra of compound 3

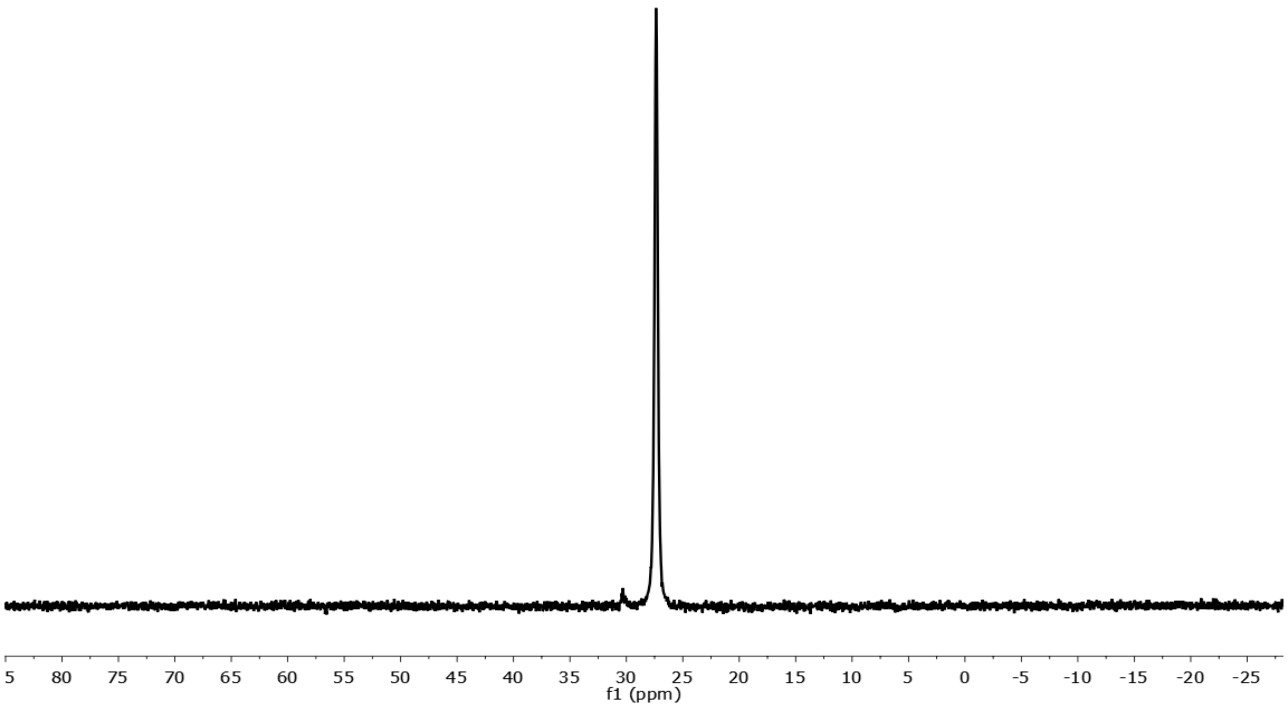

Figure S17 Detail of the ^{31}P-NMR spectra of compound 3

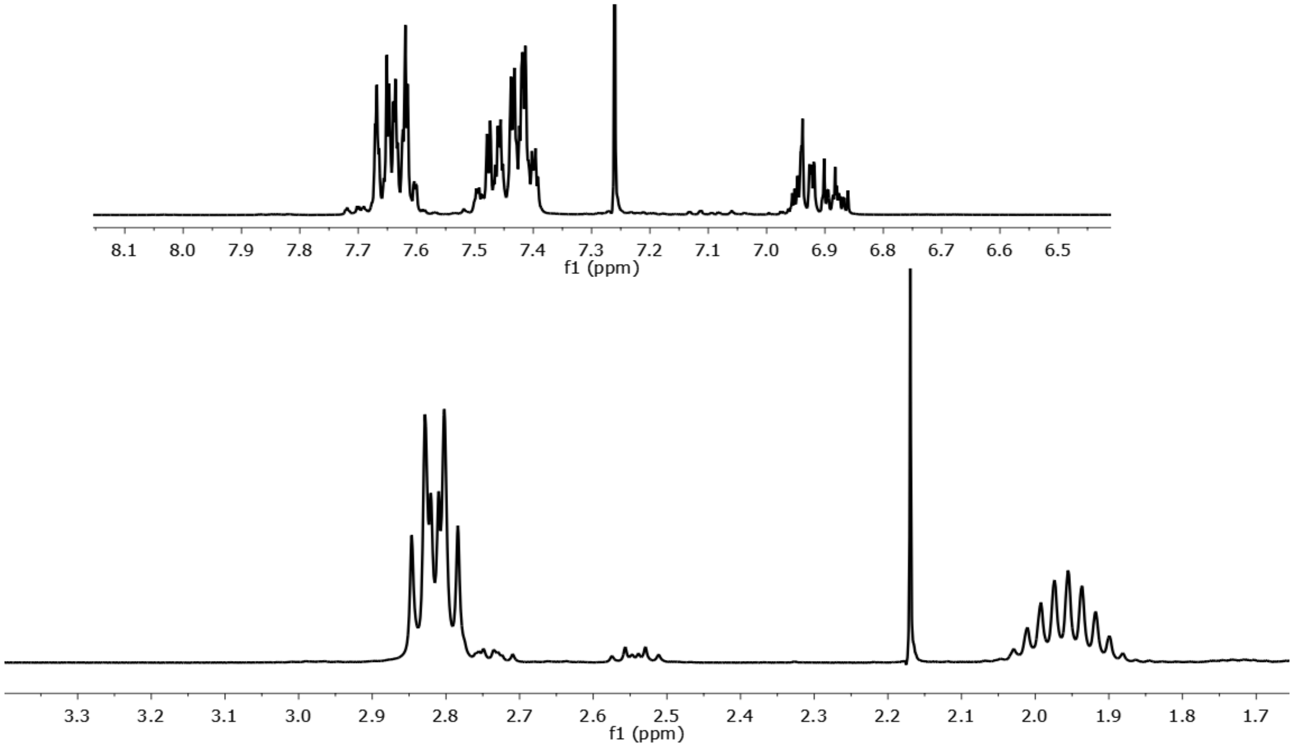

Figure S18 Detail of the ^{1}H-NMR spectra of compound 4

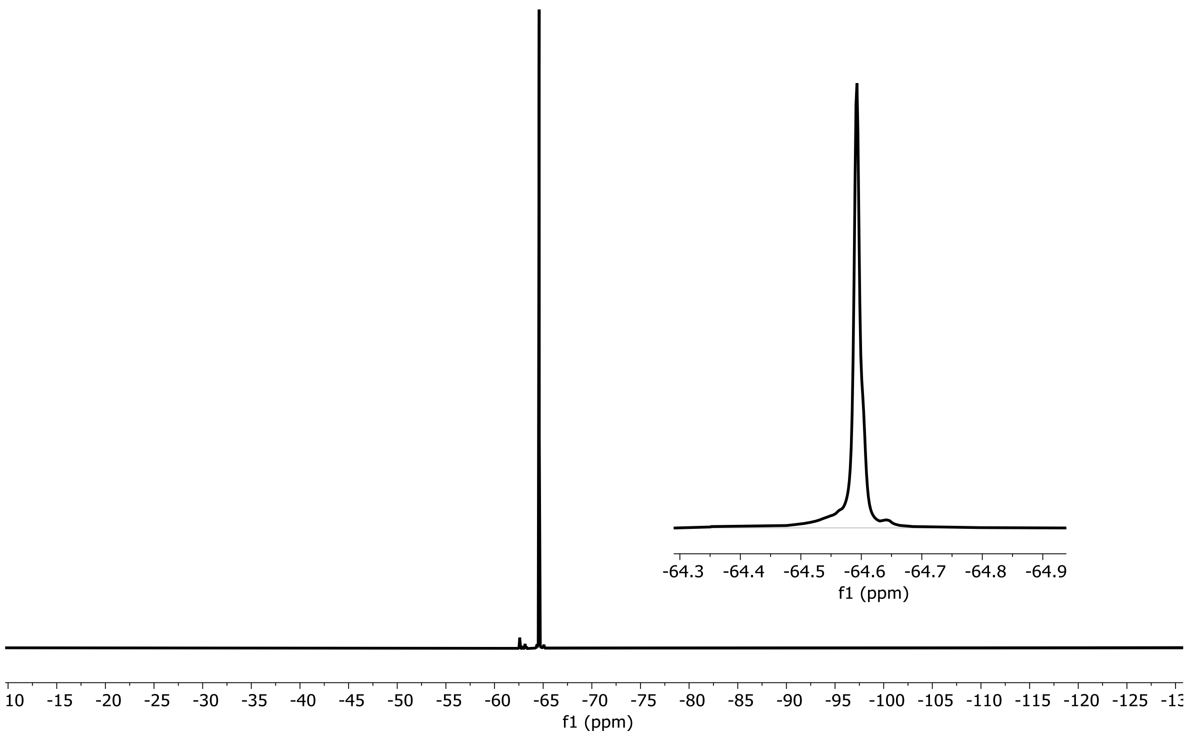

Figure S19 Detail of the ^{19}*F-NMR spectra of compound 4*

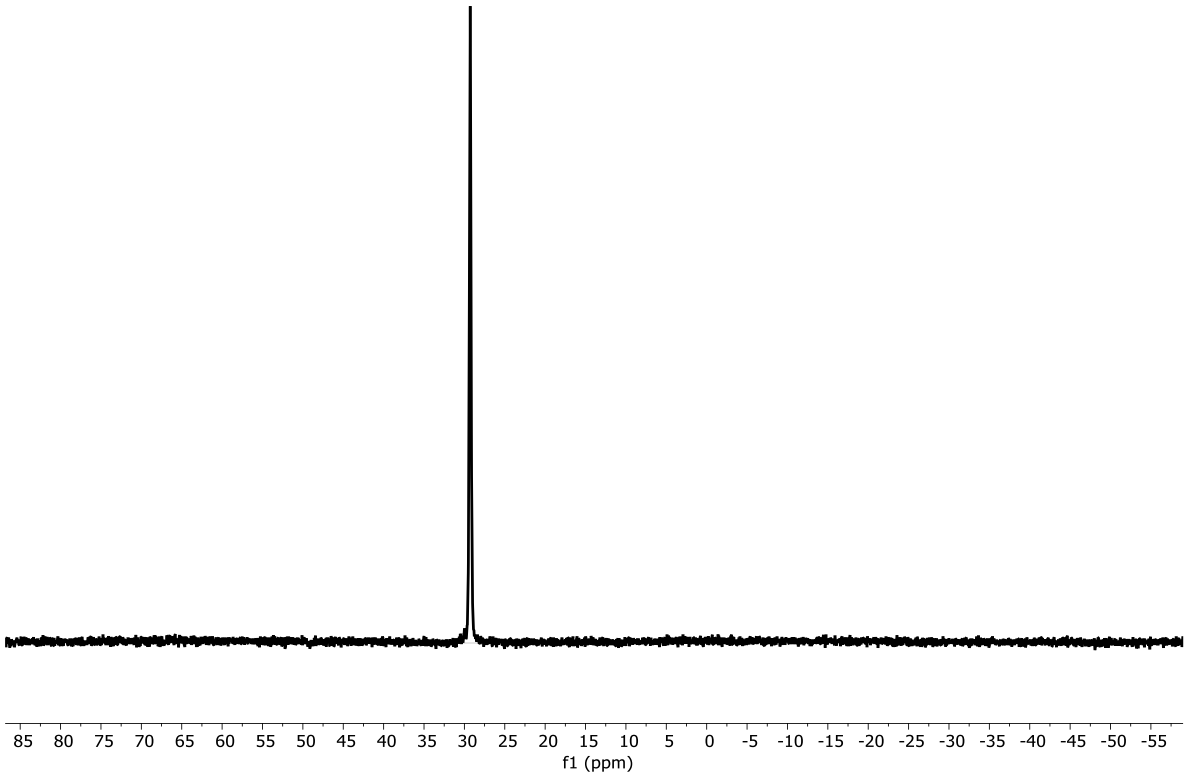

Figure S20 Detail of the ^{31}*P-NMR spectra of compound 4*

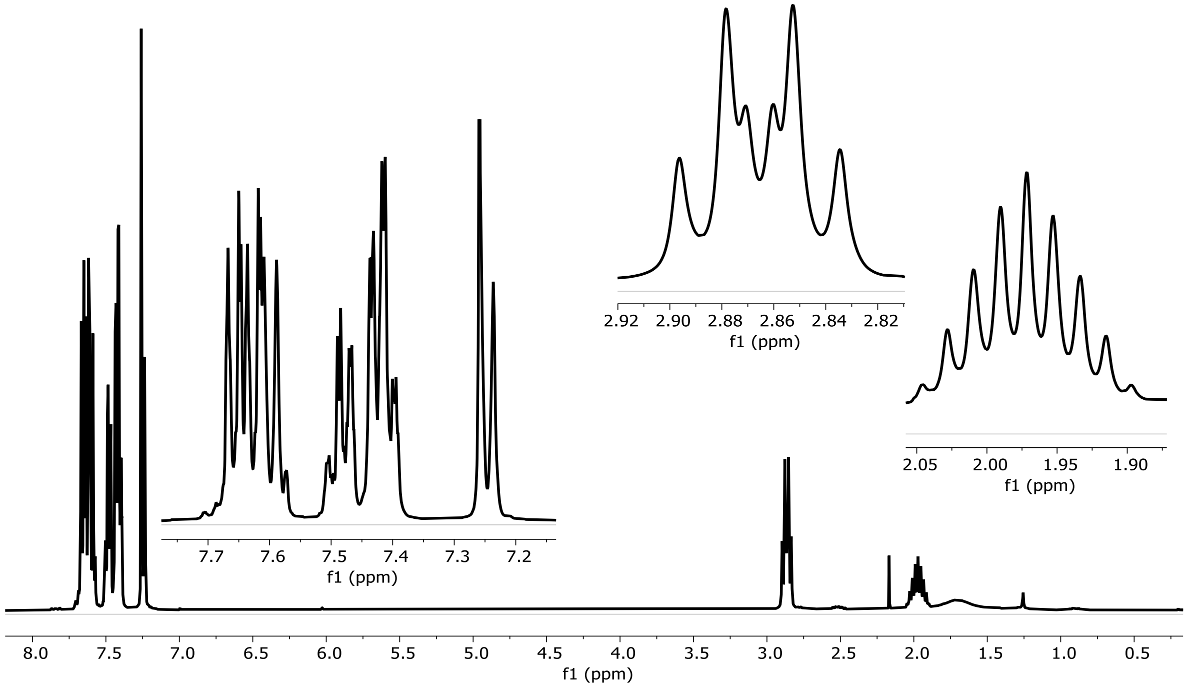

Figure S21 Detail of the ^{1}H-NMR spectra of compound 5

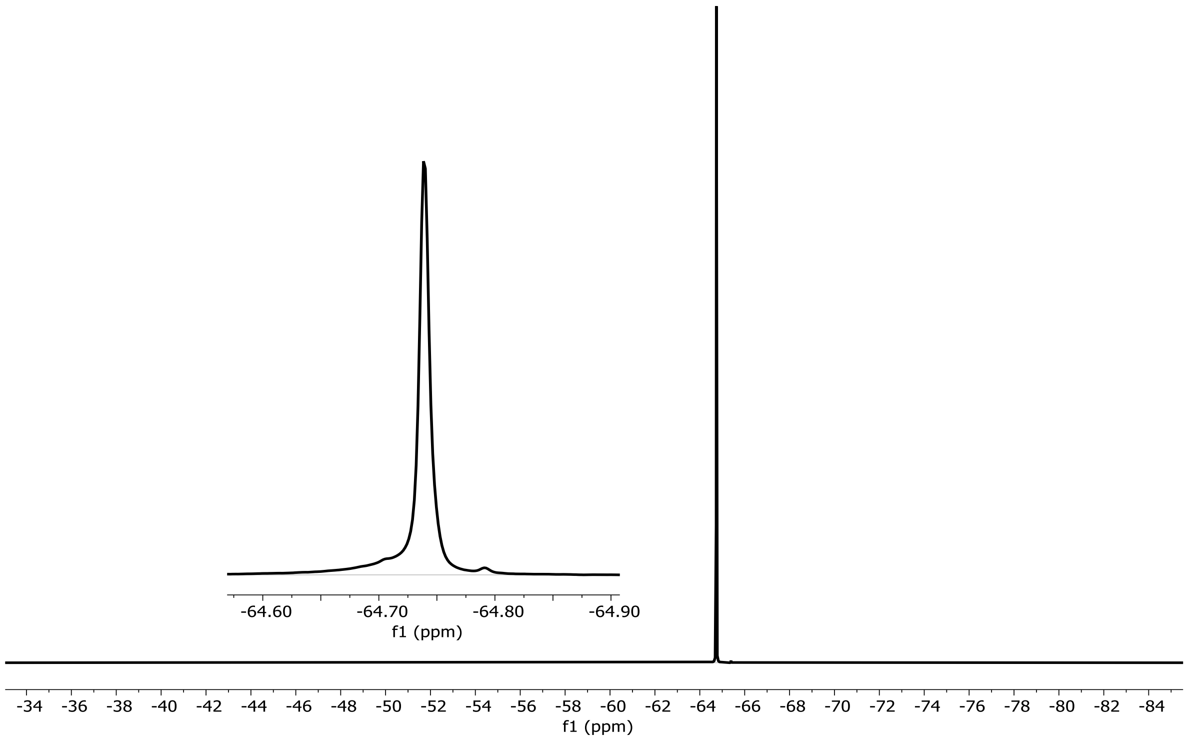

Figure S22 Detail of the ^{19}F-NMR spectra of compound 5

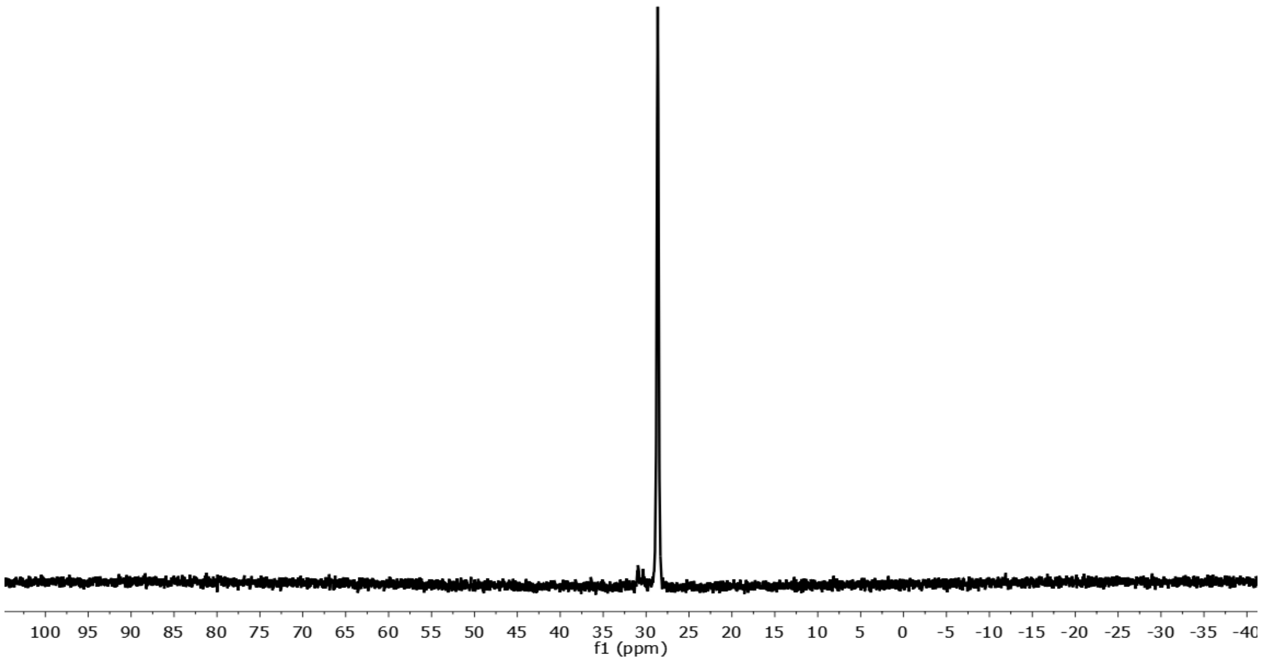

Figure S23 Detail of the ³¹P-NMR spectra of compound 5

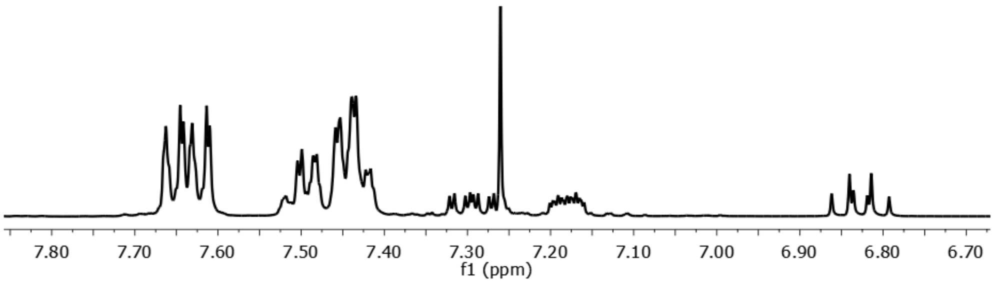

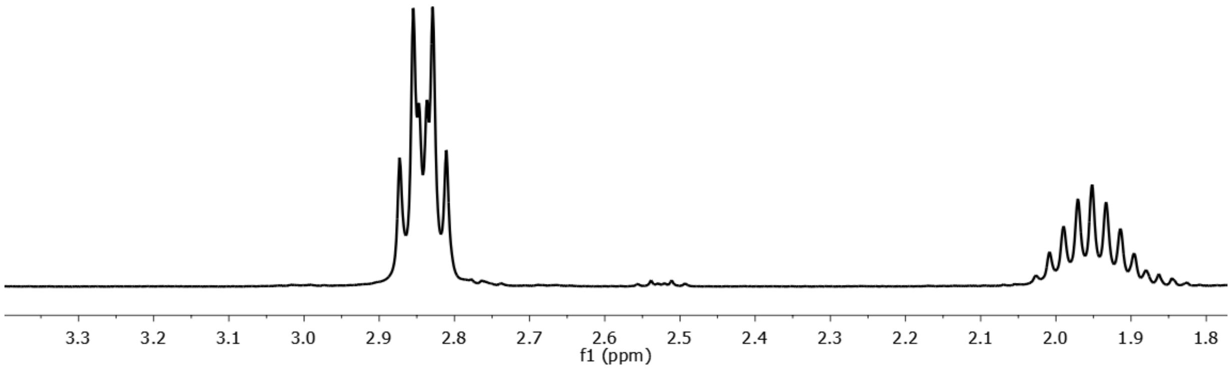

Figure S24 Detail of the ¹H-NMR spectra of compound 6

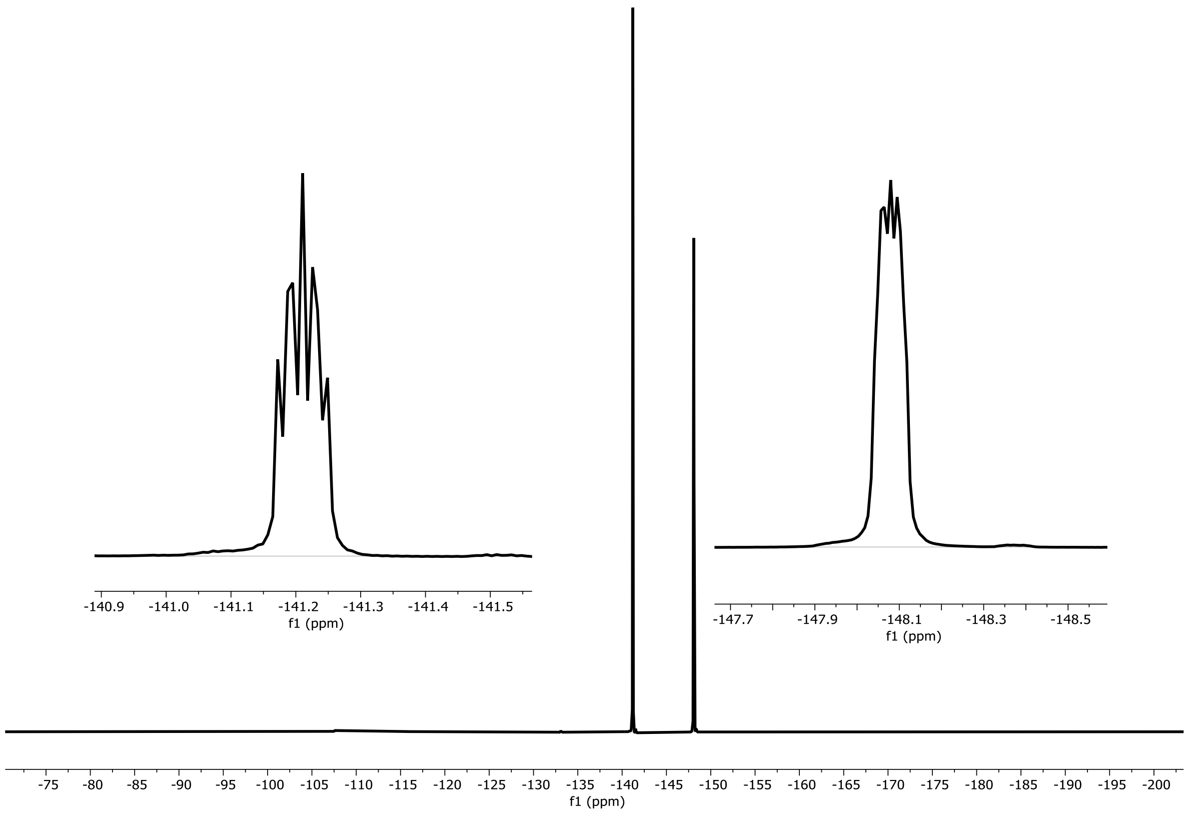

Figure S25 Detail of the ^{19}F-NMR spectra of compound 6

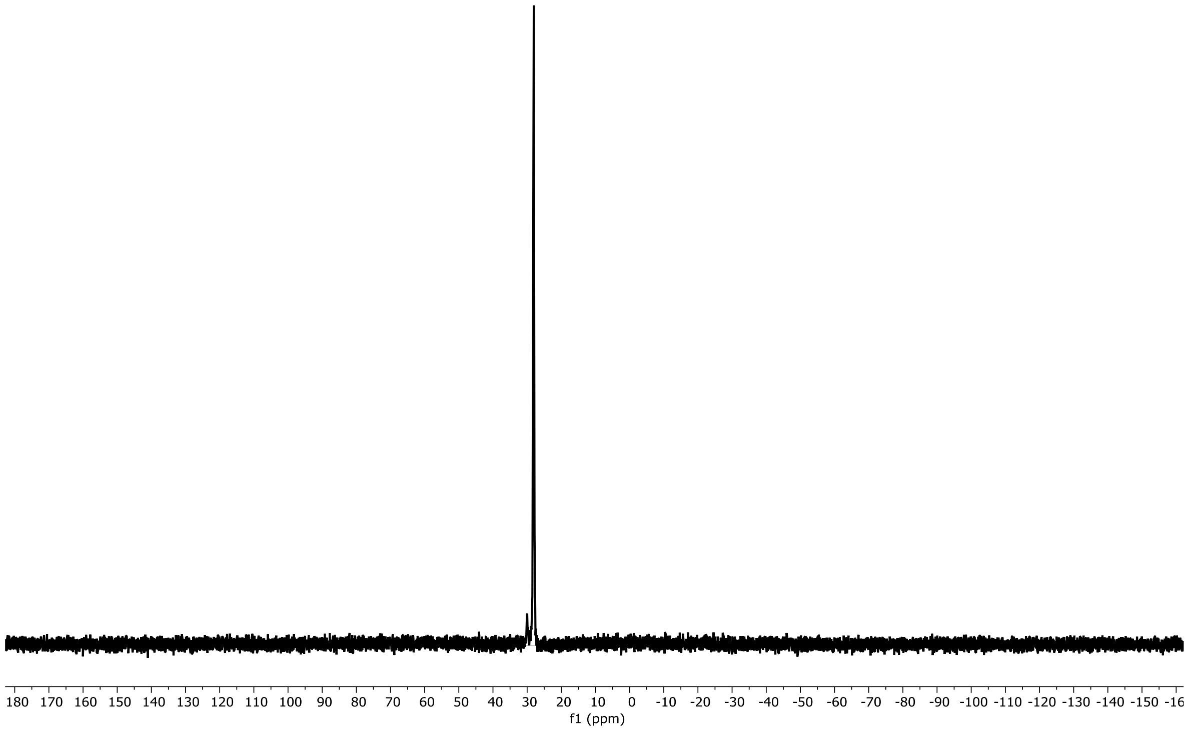

Figure S26 Detail of the ^{31}P-NMR spectra of compound 6

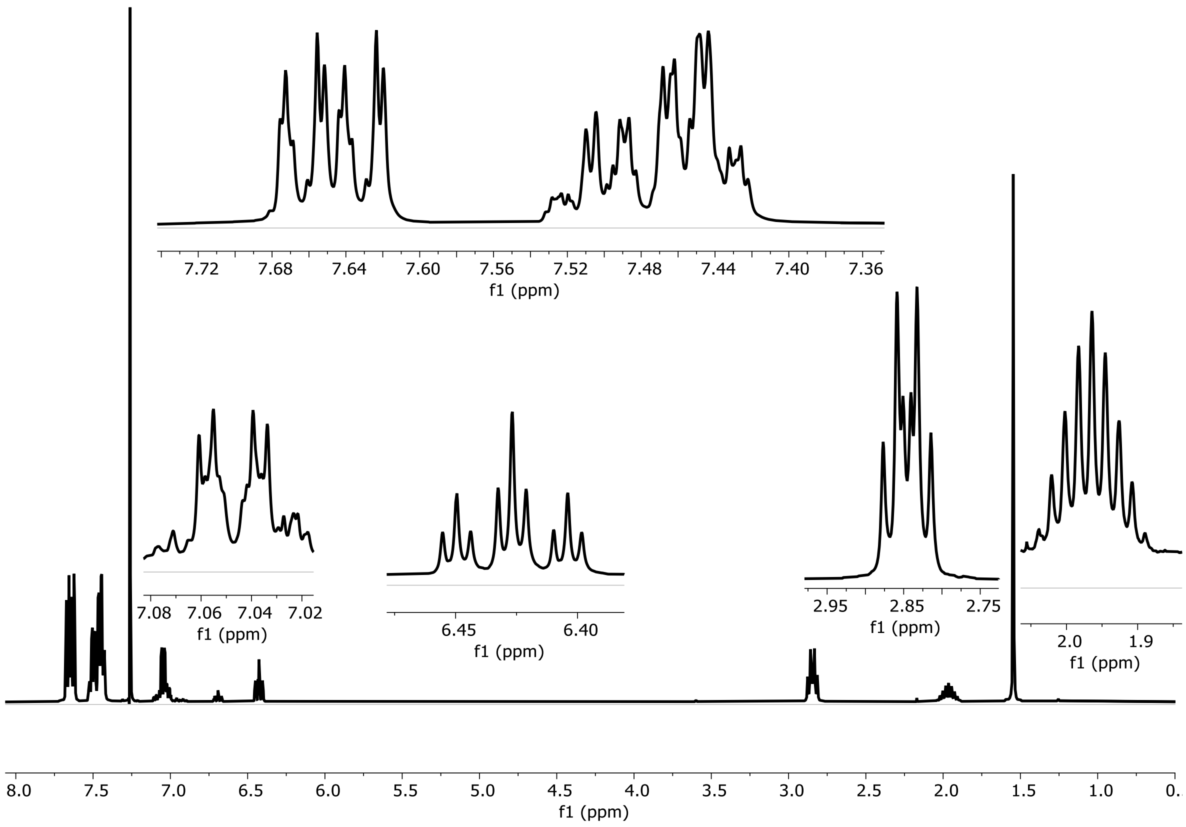

Figure S27 Detail of the ^{1}H-NMR spectra of compound 7

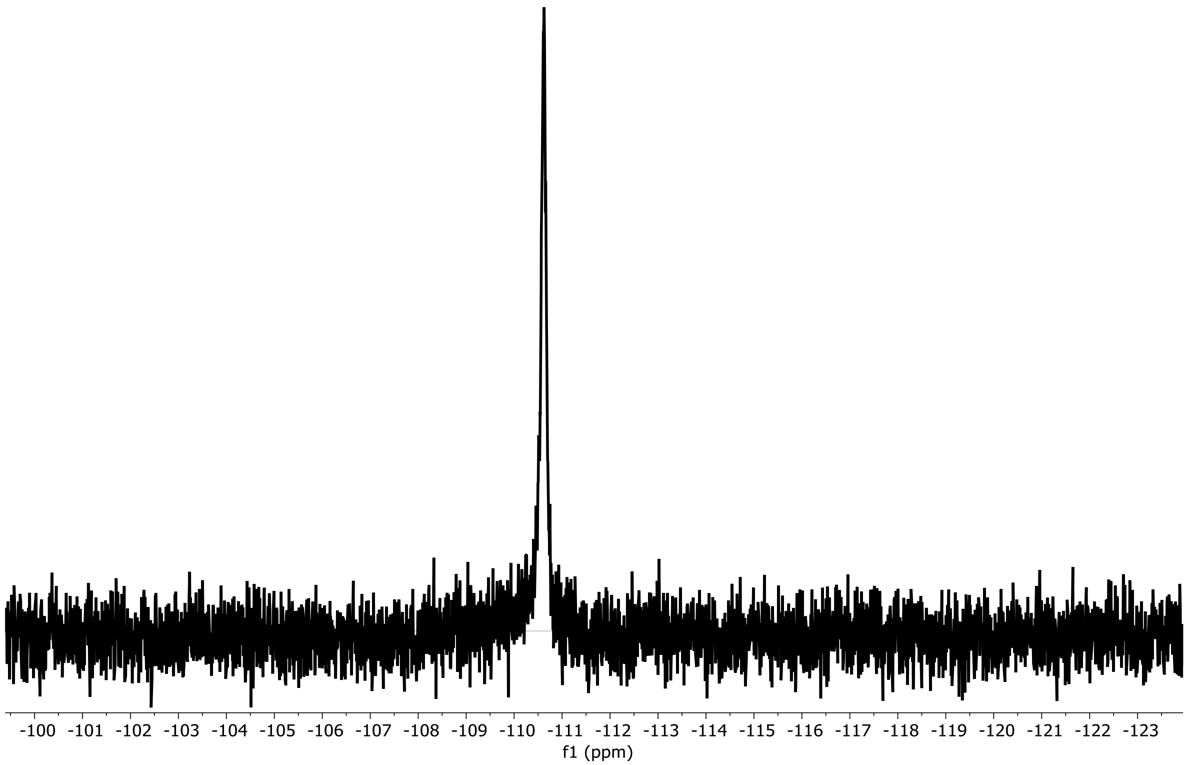

Figure S28 Detail of the ^{19}F-NMR spectra of compound 7

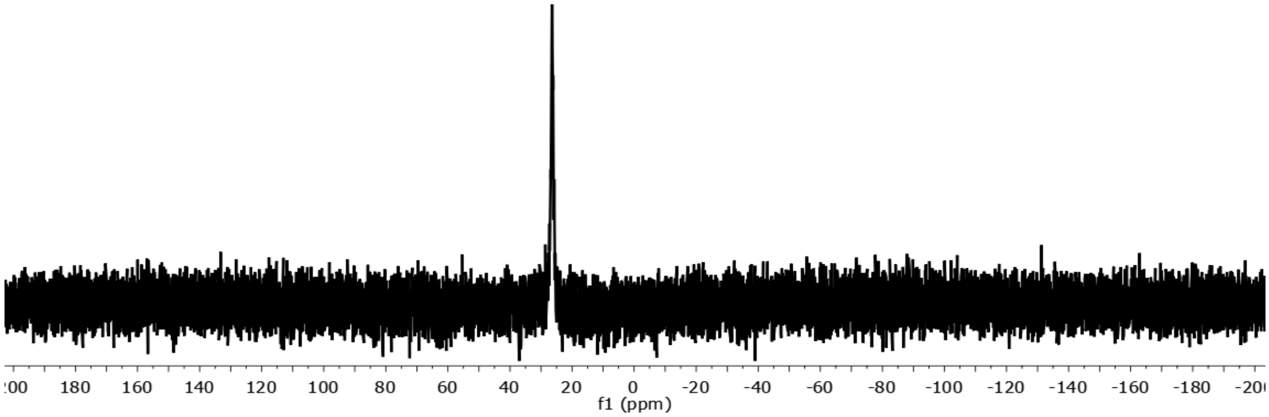

Figure S29 Detail of the ^{31}P-NMR spectra of compound 7

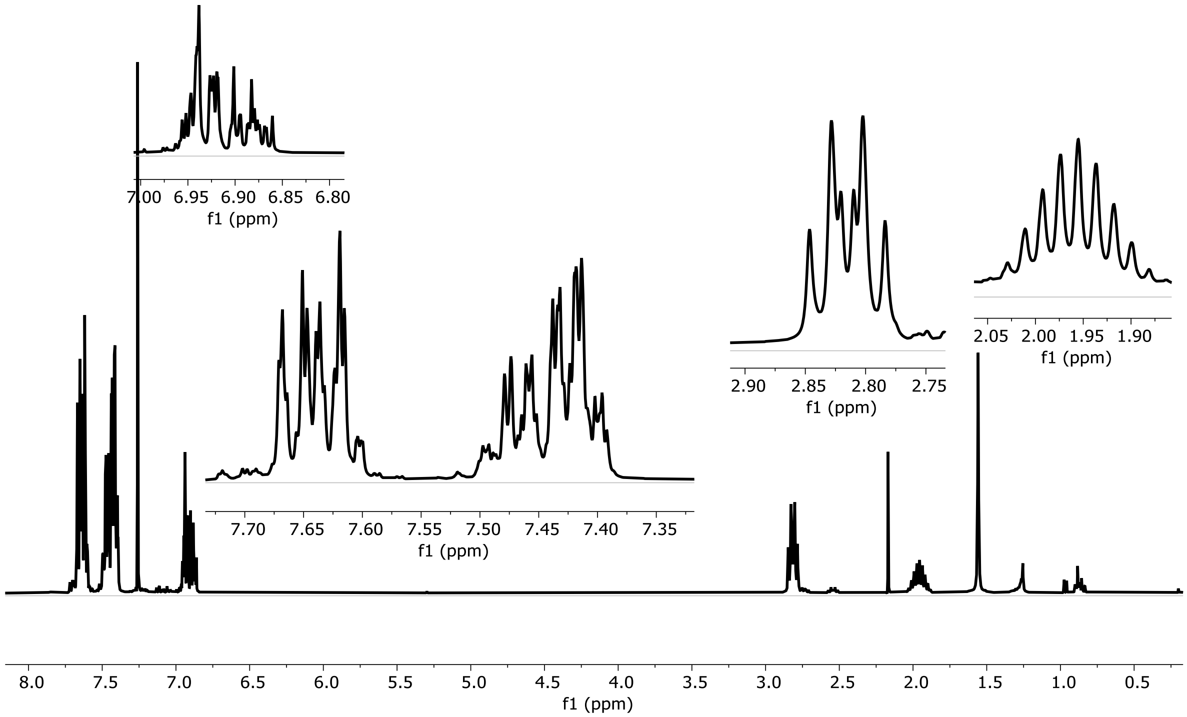

Figure S30 Detail of the ^{1}H-NMR spectra of compound 8

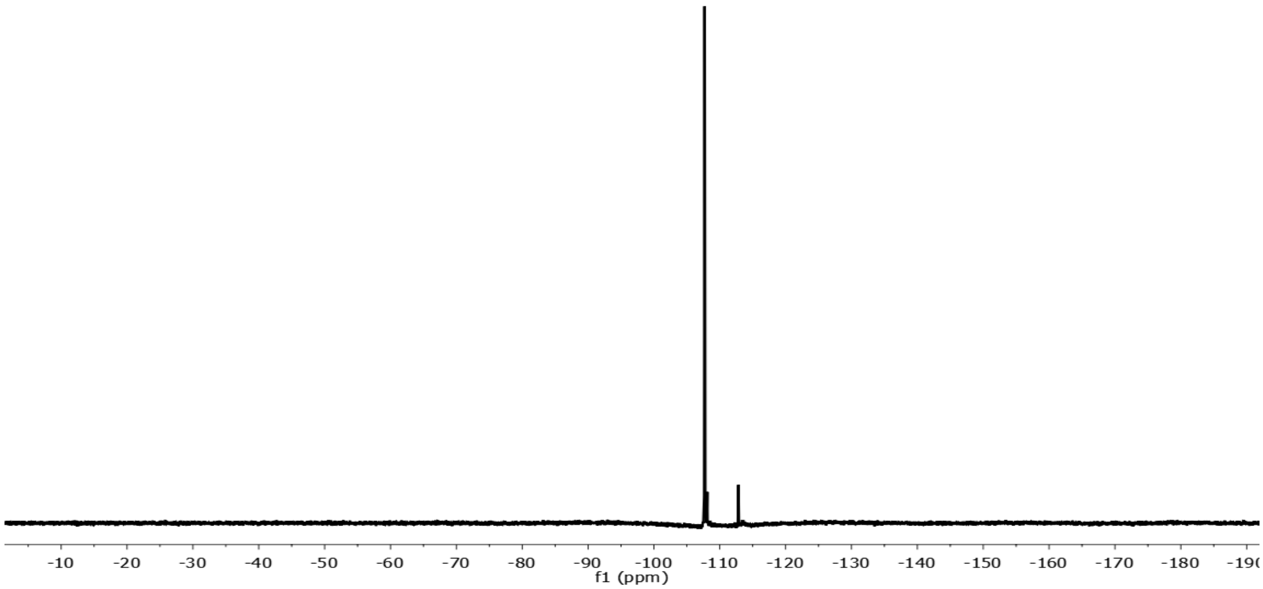

Figure S31 Detail of the ^{19}F-NMR spectra of compound 8

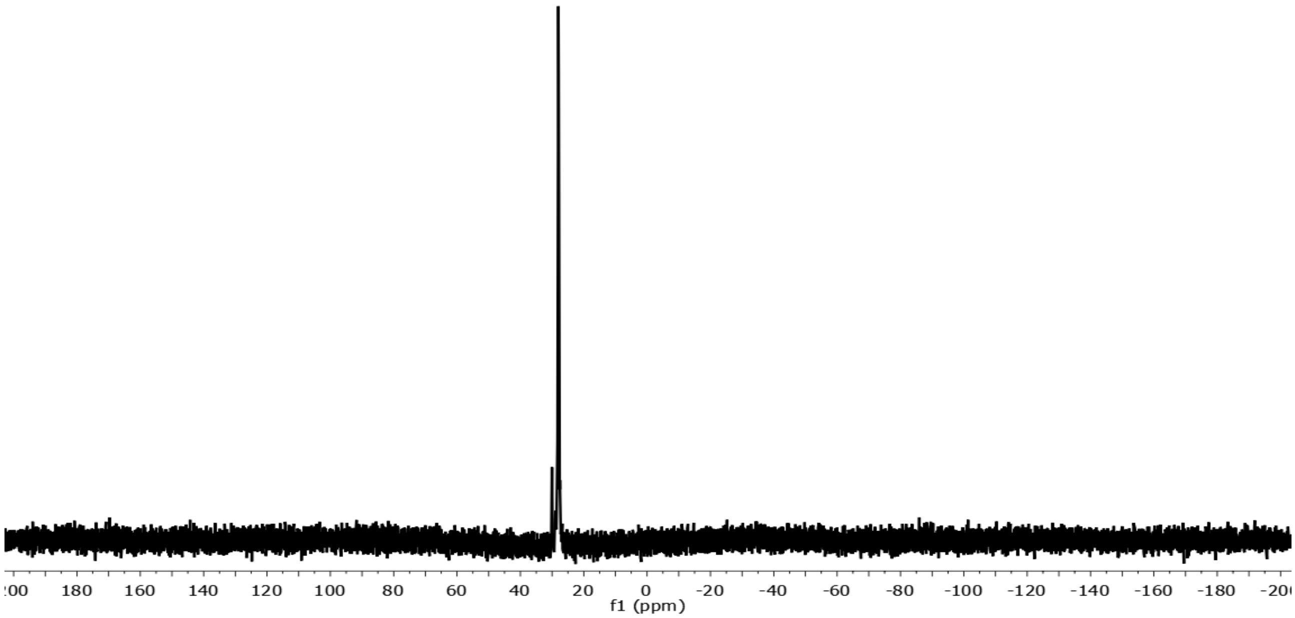

Figure S32 Detail of the ^{31}P-NMR spectra of compound 8

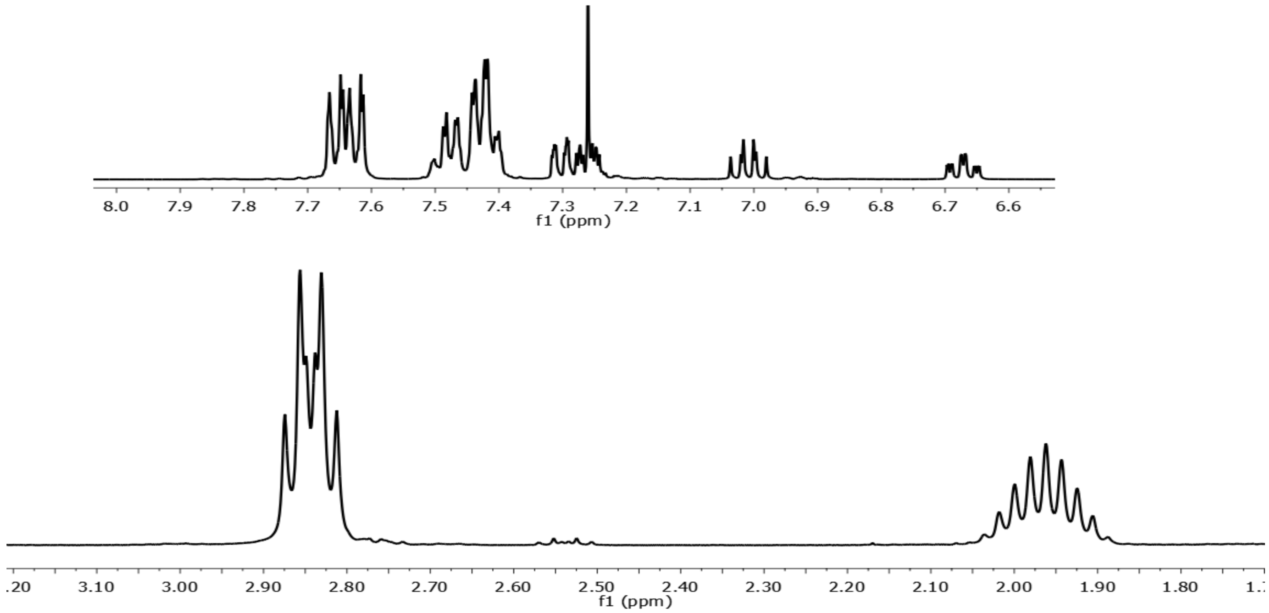

Figure S33 Detail of the ^{1}H-NMR spectra of compound 9

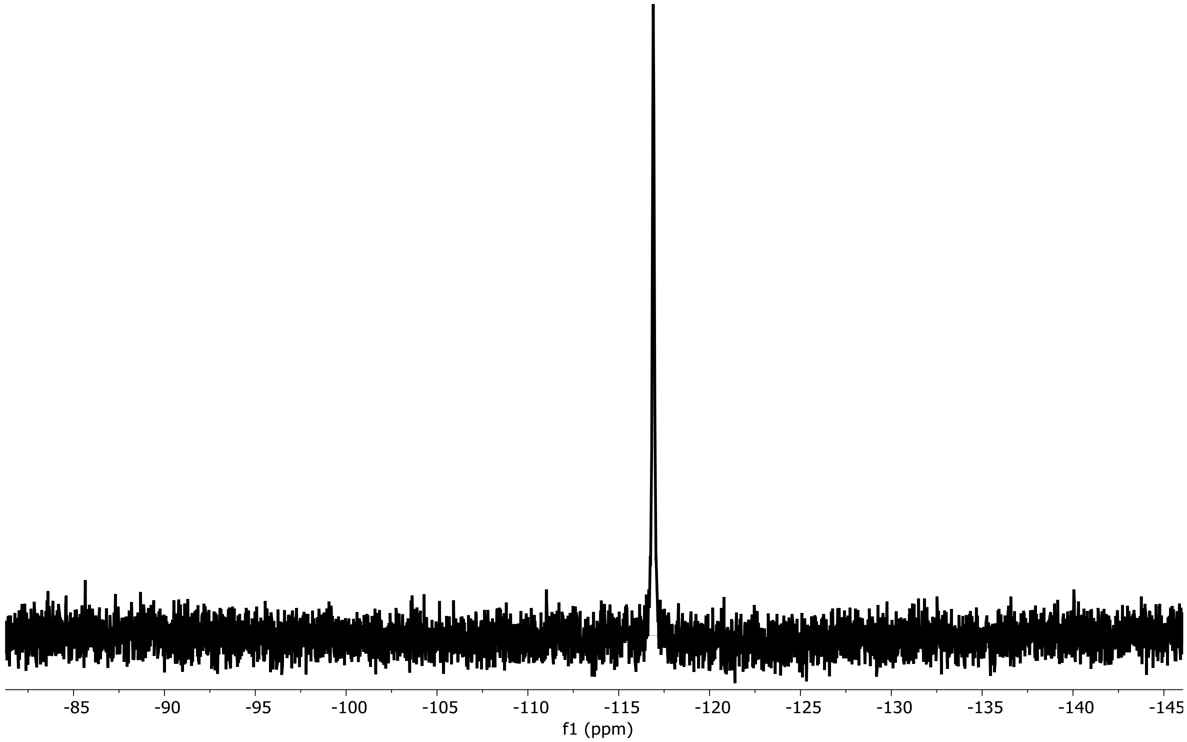

Figure S34 Detail of the ^{19}F-NMR spectra of compound 9

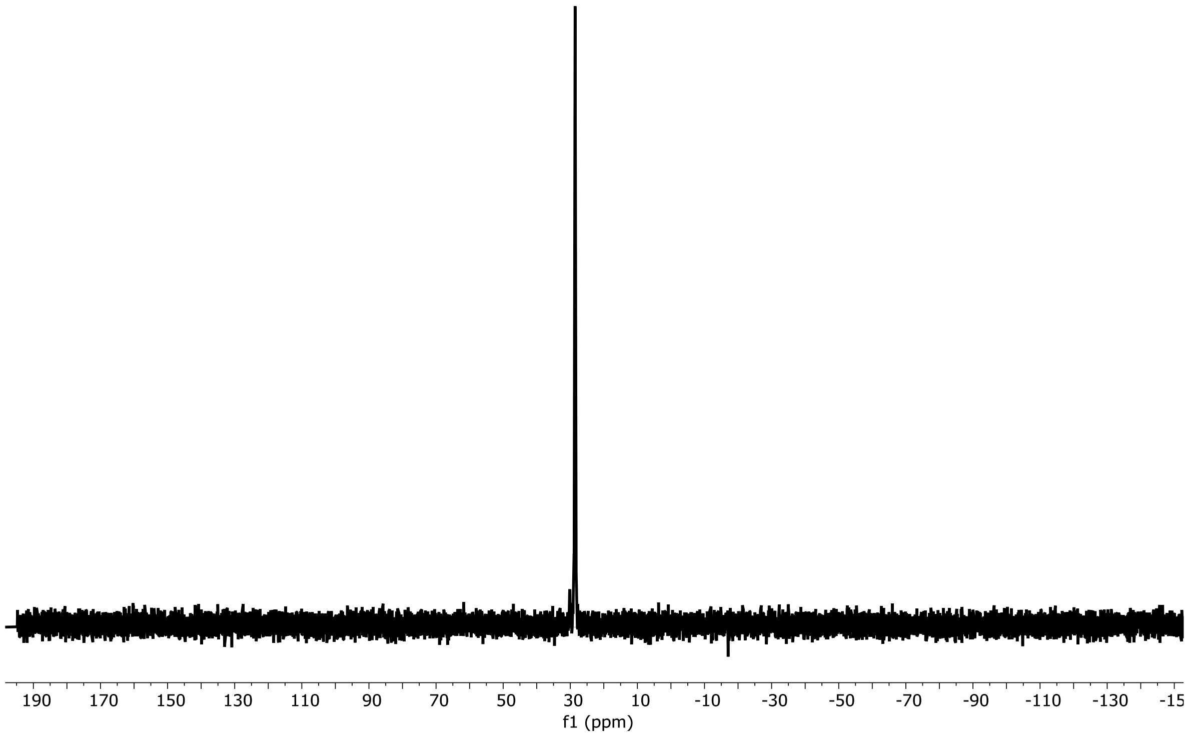

Figure S35 Detail of the ^{31}P-NMR spectra of compound 9

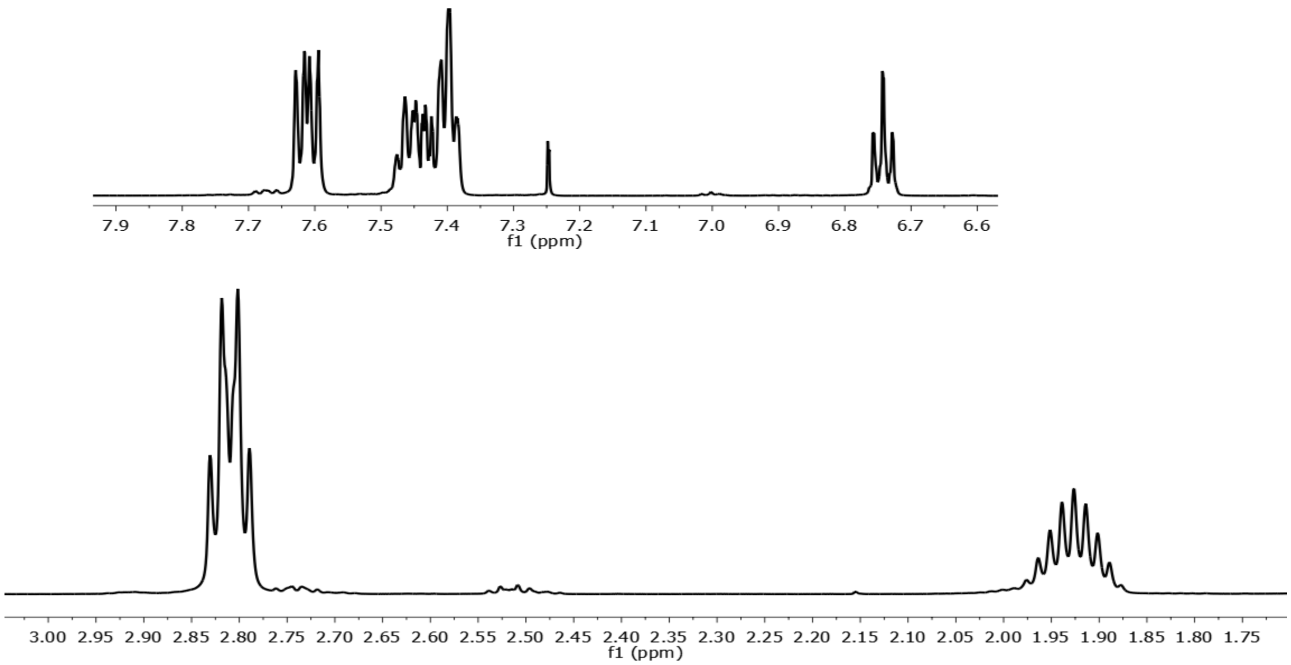

Figure S36 Detail of the ^{1}H-NMR spectra of compound 10

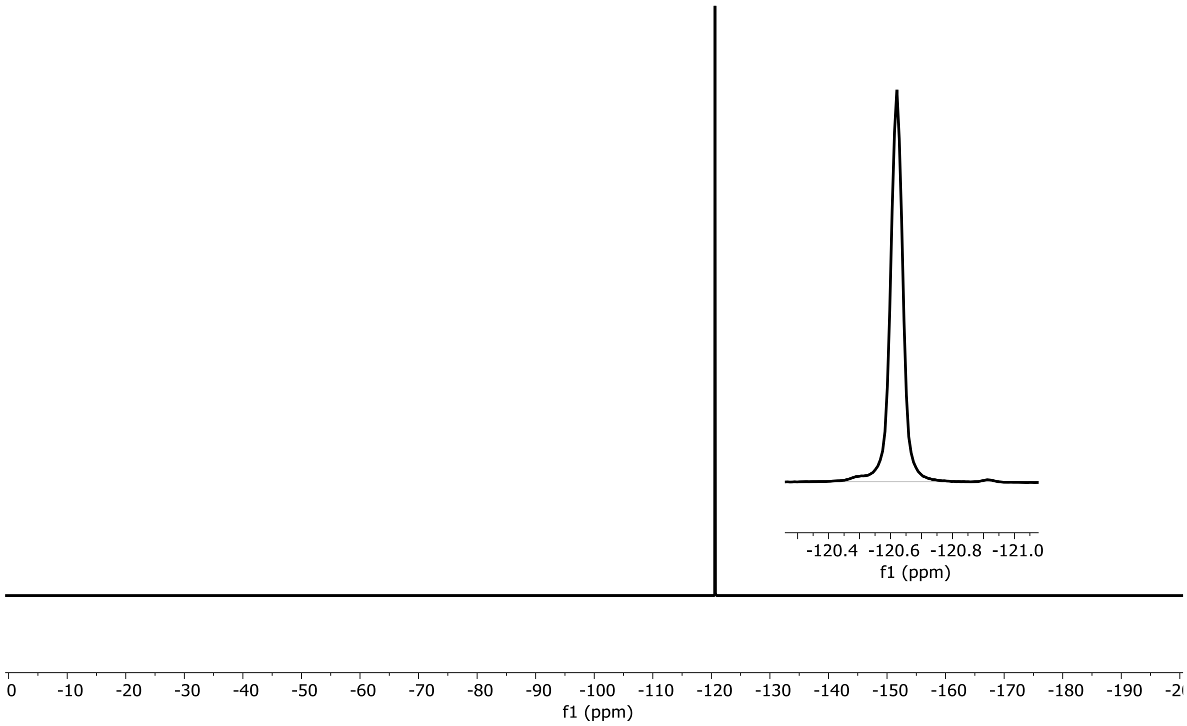

Figure S37 Detail of the ^{19}*F-NMR spectra of compound 10*

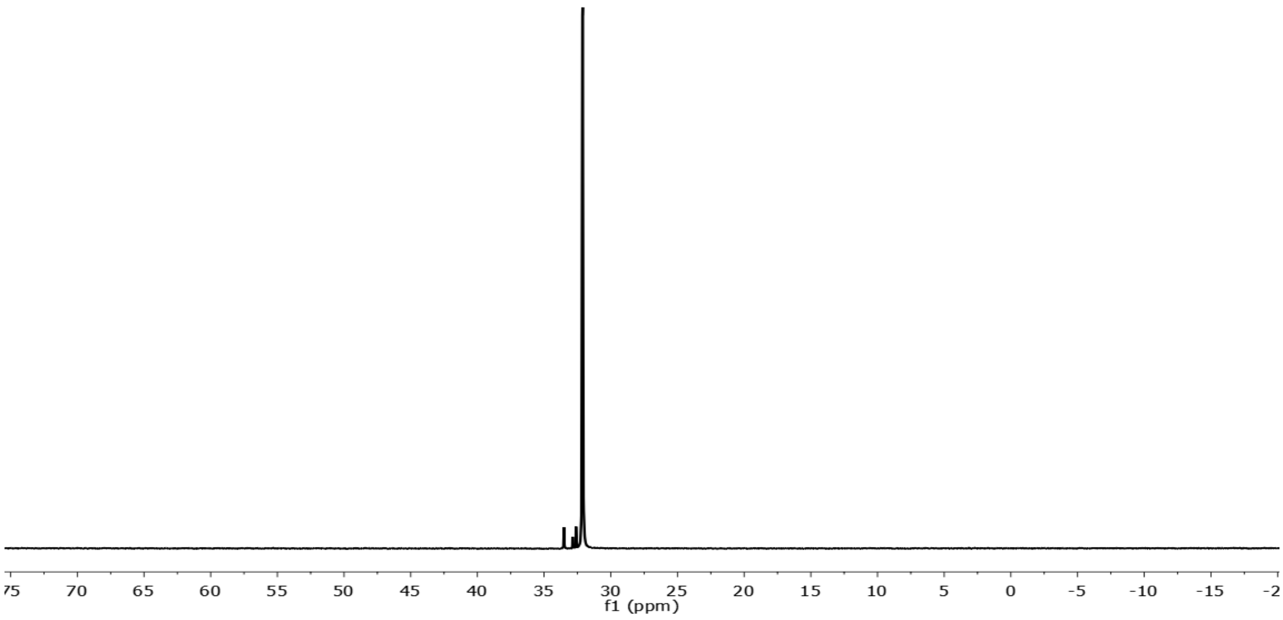

Figure S38 Detail of the ^{31}*P-NMR spectra of compound 10*

Crystallographic tables

Table S1. Crystal data and structure refinement for **1**.

Identification code	**1**	
Empirical formula	C_{39} H_{26} Au_2 F_{10} P_2 S_2	
Formula weight	1204.59	
Temperature	130(2) K	
Wavelength	0.71073 Å	
Crystal system	Monoclinic	
Space group	P 21/c	
Unit cell dimensions	a = 12.2219(5) Å	α= 90°.
	b = 25.5328(7) Å	β= 114.543(4)°.
	c = 13.5337(5) Å	γ = 90°.
Volume	3841.7(3) $Å^3$	
Z	4	
Density (calculated)	2.083 Mg/m^3	
Absorption coefficient	7.899 mm^{-1}	
F(000)	2280	
Crystal size	0.420 x 0.280 x 0.050 mm^3	
Theta range for data collection	3.405 to 29.618°.	
Index ranges	-16<=h<=16, -33<=k<=32, -17<=l<=18	
Reflections collected	67142	
Independent reflections	9883 [R(int) = 0.0694]	
Completeness to theta = 25.242°	99.8 %	
Refinement method	Full-matrix least-squares on F^2	
Data / restraints / parameters	9883 / 0 / 484	
Goodness-of-fit on F^2	1.107	
Final R indices [I>2sigma(I)]	R1 = 0.0423, wR2 = 0.0762	
R indices (all data)	R1 = 0.0646, wR2 = 0.0868	
Largest diff. peak and hole	3.623 and -1.844 e.$Å^{-3}$	

Table S2. Atomic coordinates (x 10^4) and equivalent isotropic displacement parameters ($Å^2$x 10^3) for **1**. U(eq) is defined as one third of the trace of the orthogonalized U^{ij} tensor.

	x	y	z	U(eq)
C(1)	6562(6)	3626(3)	6188(6)	31(2)
C(2)	5951(9)	3192(4)	6332(7)	47(2)
C(3)	4702(10)	3187(5)	5921(8)	64(3)
C(4)	4062(8)	3596(6)	5363(9)	66(3)
C(5)	4619(8)	4032(4)	5186(10)	69(3)
C(6)	5861(7)	4040(3)	5608(8)	50(2)
C(7)	7081(6)	3807(3)	2536(5)	24(1)
C(8)	6544(7)	3514(4)	1606(6)	40(2)
C(9)	5413(7)	3655(4)	830(7)	52(2)
C(10)	4842(8)	4086(4)	988(8)	53(3)
C(11)	5353(7)	4375(4)	1907(9)	56(3)
C(12)	6481(7)	4234(3)	2690(7)	42(2)
C(13)	9547(5)	4147(3)	3426(5)	22(1)
C(14)	10273(6)	4460(3)	4275(6)	29(2)
C(15)	11046(7)	4813(3)	4131(6)	35(2)
C(16)	11126(6)	4855(3)	3158(6)	35(2)
C(17)	10406(7)	4540(3)	2299(6)	38(2)
C(18)	9616(7)	4196(3)	2434(6)	34(2)
C(19)	9037(5)	3053(3)	3217(5)	21(1)
C(20)	7538(6)	1617(3)	4336(5)	23(1)
C(21)	8528(6)	1298(3)	4553(5)	23(1)
C(22)	8443(6)	777(3)	4293(5)	27(1)
C(23)	7334(7)	551(3)	3745(6)	33(2)
C(24)	6327(7)	850(3)	3488(6)	33(2)
C(25)	6429(6)	1367(3)	3768(5)	26(1)
C(26)	12230(6)	2351(3)	7736(6)	30(1)
C(27)	13382(6)	2522(3)	8376(6)	30(1)
C(28)	14368(7)	2215(3)	8476(7)	40(2)
C(29)	14193(7)	1749(4)	7937(7)	46(2)
C(30)	13035(8)	1572(4)	7302(8)	59(3)
C(31)	12063(7)	1880(3)	7213(7)	45(2)
C(32)	11403(5)	3418(3)	7682(5)	25(1)
C(33)	11239(7)	3772(3)	8383(6)	34(2)
C(34)	11576(7)	4291(3)	8400(7)	43(2)
C(35)	12099(7)	4458(3)	7733(7)	43(2)
C(36)	12276(7)	4112(3)	7036(7)	41(2)
C(37)	11929(6)	3593(3)	6997(6)	32(2)

C(38)	10718(6)	2634(3)	8837(5)	21(1)
C(39)	10346(6)	2071(3)	8973(5)	25(1)
Au(1)	8472(1)	3653(1)	5231(1)	22(1)
Au(2)	9278(1)	2530(1)	6072(1)	21(1)
F(1)	6551(6)	2778(2)	6873(4)	77(2)
F(2)	4144(7)	2765(3)	6066(5)	110(3)
F(3)	2848(5)	3593(3)	4953(6)	107(3)
F(4)	3993(5)	4439(3)	4592(8)	128(4)
F(5)	6384(5)	4461(2)	5401(6)	78(2)
F(6)	9648(3)	1495(2)	5077(3)	33(1)
F(7)	9439(4)	493(2)	4517(3)	38(1)
F(8)	7249(4)	41(2)	3462(4)	51(1)
F(9)	5229(4)	640(2)	2925(4)	52(1)
F(10)	5407(3)	1649(2)	3473(3)	35(1)
P(1)	8561(1)	3663(1)	3601(1)	19(1)
P(2)	10927(1)	2739(1)	7588(1)	21(1)
S(1)	8152(2)	3653(1)	6796(1)	31(1)
S(2)	7535(2)	2289(1)	4609(1)	26(1)

Table S3. Bond lengths [Å] and angles [°] for **1**.

C(1)-C(6)	1.383(11)
C(1)-C(2)	1.394(11)
C(1)-S(1)	1.769(7)
C(2)-F(1)	1.321(11)
C(2)-C(3)	1.390(14)
C(3)-F(2)	1.335(11)
C(3)-C(4)	1.335(16)
C(4)-F(3)	1.351(10)
C(4)-C(5)	1.376(16)
C(5)-F(4)	1.343(13)
C(5)-C(6)	1.382(11)
C(6)-F(5)	1.338(10)
C(7)-C(8)	1.374(10)
C(7)-C(12)	1.377(10)
C(7)-P(1)	1.822(7)
C(8)-C(9)	1.392(11)
C(8)-H(8)	0.9500
C(9)-C(10)	1.366(14)
C(9)-H(9)	0.9500
C(10)-C(11)	1.356(14)
C(10)-H(10)	0.9500
C(11)-C(12)	1.393(11)
C(11)-H(11)	0.9500
C(12)-H(12)	0.9500
C(13)-C(14)	1.377(9)
C(13)-C(18)	1.386(9)
C(13)-P(1)	1.808(7)
C(14)-C(15)	1.377(10)
C(14)-H(14)	0.9500
C(15)-C(16)	1.365(10)
C(15)-H(15)	0.9500
C(16)-C(17)	1.386(11)
C(16)-H(16)	0.9500
C(17)-C(18)	1.374(10)
C(17)-H(17)	0.9500
C(18)-H(18)	0.9500
C(19)-C(39)#1	1.533(8)
C(19)-P(1)	1.812(6)
C(19)-H(19A)	0.9900

C(19)-H(19B)	0.9900
C(20)-C(21)	1.384(9)
C(20)-C(25)	1.404(9)
C(20)-S(2)	1.757(7)
C(21)-F(6)	1.349(7)
C(21)-C(22)	1.369(9)
C(22)-F(7)	1.341(8)
C(22)-C(23)	1.373(10)
C(23)-F(8)	1.349(8)
C(23)-C(24)	1.366(10)
C(24)-F(9)	1.348(8)
C(24)-C(25)	1.364(10)
C(25)-F(10)	1.349(8)
C(26)-C(31)	1.368(11)
C(26)-C(27)	1.382(9)
C(26)-P(2)	1.815(7)
C(27)-C(28)	1.396(10)
C(27)-H(27)	0.9500
C(28)-C(29)	1.366(12)
C(28)-H(28)	0.9500
C(29)-C(30)	1.392(12)
C(29)-H(29)	0.9500
C(30)-C(31)	1.388(11)
C(30)-H(30)	0.9500
C(31)-H(31)	0.9500
C(32)-C(33)	1.385(10)
C(32)-C(37)	1.402(9)
C(32)-P(2)	1.817(7)
C(33)-C(34)	1.384(10)
C(33)-H(33)	0.9500
C(34)-C(35)	1.372(11)
C(34)-H(34)	0.9500
C(35)-C(36)	1.374(12)
C(35)-H(35)	0.9500
C(36)-C(37)	1.387(11)
C(36)-H(36)	0.9500
C(37)-H(37)	0.9500
C(38)-C(39)	1.540(9)
C(38)-P(2)	1.832(6)

C(38)-H(38A)	0.9900
C(38)-H(38B)	0.9900
C(39)-C(19)#2	1.533(8)
C(39)-H(39A)	0.9900
C(39)-H(39B)	0.9900
Au(1)-P(1)	2.2535(16)
Au(1)-S(1)	2.3085(17)
Au(1)-Au(2)	3.0923(4)
Au(2)-P(2)	2.2629(17)
Au(2)-S(2)	2.3085(17)
C(6)-C(1)-C(2)	116.5(8)
C(6)-C(1)-S(1)	122.4(6)
C(2)-C(1)-S(1)	121.0(7)
F(1)-C(2)-C(3)	118.6(9)
F(1)-C(2)-C(1)	120.5(8)
C(3)-C(2)-C(1)	120.9(10)
F(2)-C(3)-C(4)	120.0(11)
F(2)-C(3)-C(2)	119.4(12)
C(4)-C(3)-C(2)	120.5(9)
C(3)-C(4)-F(3)	120.9(11)
C(3)-C(4)-C(5)	120.9(9)
F(3)-C(4)-C(5)	118.2(12)
F(4)-C(5)-C(4)	122.0(9)
F(4)-C(5)-C(6)	119.3(10)
C(4)-C(5)-C(6)	118.7(11)
F(5)-C(6)-C(5)	117.7(9)
F(5)-C(6)-C(1)	119.8(7)
C(5)-C(6)-C(1)	122.5(9)
C(8)-C(7)-C(12)	119.2(7)
C(8)-C(7)-P(1)	123.7(5)
C(12)-C(7)-P(1)	117.1(6)
C(7)-C(8)-C(9)	119.8(8)
C(7)-C(8)-H(8)	120.1
C(9)-C(8)-H(8)	120.1
C(10)-C(9)-C(8)	120.2(9)
C(10)-C(9)-H(9)	119.9
C(8)-C(9)-H(9)	119.9
C(11)-C(10)-C(9)	120.8(8)
C(11)-C(10)-H(10)	119.6
C(9)-C(10)-H(10)	119.6
C(10)-C(11)-C(12)	119.4(9)

C(10)-C(11)-H(11)	120.3
C(12)-C(11)-H(11)	120.3
C(7)-C(12)-C(11)	120.7(8)
C(7)-C(12)-H(12)	119.7
C(11)-C(12)-H(12)	119.7
C(14)-C(13)-C(18)	118.6(6)
C(14)-C(13)-P(1)	121.4(5)
C(18)-C(13)-P(1)	120.0(5)
C(13)-C(14)-C(15)	120.2(7)
C(13)-C(14)-H(14)	119.9
C(15)-C(14)-H(14)	119.9
C(16)-C(15)-C(14)	121.1(7)
C(16)-C(15)-H(15)	119.5
C(14)-C(15)-H(15)	119.5
C(15)-C(16)-C(17)	119.3(7)
C(15)-C(16)-H(16)	120.4
C(17)-C(16)-H(16)	120.4
C(18)-C(17)-C(16)	119.7(7)
C(18)-C(17)-H(17)	120.1
C(16)-C(17)-H(17)	120.1
C(17)-C(18)-C(13)	121.0(7)
C(17)-C(18)-H(18)	119.5
C(13)-C(18)-H(18)	119.5
C(39)#1-C(19)-P(1)	110.9(4)
C(39)#1-C(19)-H(19A)	109.5
P(1)-C(19)-H(19A)	109.5
C(39)#1-C(19)-H(19B)	109.5
P(1)-C(19)-H(19B)	109.5
H(19A)-C(19)-H(19B)	108.0
C(21)-C(20)-C(25)	114.2(6)
C(21)-C(20)-S(2)	127.4(5)
C(25)-C(20)-S(2)	118.2(5)
F(6)-C(21)-C(22)	116.7(6)
F(6)-C(21)-C(20)	119.9(6)
C(22)-C(21)-C(20)	123.3(6)
F(7)-C(22)-C(21)	120.2(6)
F(7)-C(22)-C(23)	119.6(6)
C(21)-C(22)-C(23)	120.1(6)
F(8)-C(23)-C(24)	120.9(7)
F(8)-C(23)-C(22)	120.2(7)
C(24)-C(23)-C(22)	118.9(7)
F(9)-C(24)-C(25)	119.7(7)

F(9)-C(24)-C(23)	120.1(7)
C(25)-C(24)-C(23)	120.2(7)
F(10)-C(25)-C(24)	117.8(6)
F(10)-C(25)-C(20)	119.0(6)
C(24)-C(25)-C(20)	123.2(7)
C(31)-C(26)-C(27)	119.8(7)
C(31)-C(26)-P(2)	119.1(5)
C(27)-C(26)-P(2)	121.1(6)
C(26)-C(27)-C(28)	119.8(7)
C(26)-C(27)-H(27)	120.1
C(28)-C(27)-H(27)	120.1
C(29)-C(28)-C(27)	120.0(7)
C(29)-C(28)-H(28)	120.0
C(27)-C(28)-H(28)	120.0
C(28)-C(29)-C(30)	120.4(8)
C(28)-C(29)-H(29)	119.8
C(30)-C(29)-H(29)	119.8
C(31)-C(30)-C(29)	118.9(9)
C(31)-C(30)-H(30)	120.5
C(29)-C(30)-H(30)	120.5
C(26)-C(31)-C(30)	121.0(8)
C(26)-C(31)-H(31)	119.5
C(30)-C(31)-H(31)	119.5
C(33)-C(32)-C(37)	118.8(7)
C(33)-C(32)-P(2)	122.6(5)
C(37)-C(32)-P(2)	118.6(6)
C(34)-C(33)-C(32)	120.6(7)
C(34)-C(33)-H(33)	119.7
C(32)-C(33)-H(33)	119.7
C(35)-C(34)-C(33)	120.2(8)
C(35)-C(34)-H(34)	119.9
C(33)-C(34)-H(34)	119.9
C(34)-C(35)-C(36)	120.0(8)
C(34)-C(35)-H(35)	120.0
C(36)-C(35)-H(35)	120.0
C(35)-C(36)-C(37)	120.6(7)
C(35)-C(36)-H(36)	119.7

C(37)-C(36)-H(36)	119.7
C(36)-C(37)-C(32)	119.7(7)
C(36)-C(37)-H(37)	120.1
C(32)-C(37)-H(37)	120.1
C(39)-C(38)-P(2)	114.1(4)
C(39)-C(38)-H(38A)	108.7
P(2)-C(38)-H(38A)	108.7
C(39)-C(38)-H(38B)	108.7
P(2)-C(38)-H(38B)	108.7
H(38A)-C(38)-H(38B)	107.6
C(19)#2-C(39)-C(38)	113.1(5)
C(19)#2-C(39)-H(39A)	109.0
C(38)-C(39)-H(39A)	109.0
C(19)#2-C(39)-H(39B)	109.0
C(38)-C(39)-H(39B)	109.0
H(39A)-C(39)-H(39B)	107.8
P(1)-Au(1)-S(1)	173.59(6)
P(1)-Au(1)-Au(2)	103.54(4)
S(1)-Au(1)-Au(2)	79.16(5)
P(2)-Au(2)-S(2)	175.72(6)
P(2)-Au(2)-Au(1)	98.17(4)
S(2)-Au(2)-Au(1)	83.76(4)
C(13)-P(1)-C(19)	103.9(3)
C(13)-P(1)-C(7)	104.8(3)
C(19)-P(1)-C(7)	106.1(3)
C(13)-P(1)-Au(1)	116.2(2)
C(19)-P(1)-Au(1)	115.0(2)
C(7)-P(1)-Au(1)	109.9(2)
C(26)-P(2)-C(32)	105.8(3)
C(26)-P(2)-C(38)	105.7(3)
C(32)-P(2)-C(38)	103.8(3)
C(26)-P(2)-Au(2)	112.4(2)
C(32)-P(2)-Au(2)	115.3(2)
C(38)-P(2)-Au(2)	112.9(2)
C(1)-S(1)-Au(1)	98.4(2)
C(20)-S(2)-Au(2)	111.0(2)

Table S4. Crystal data and structure refinement for **2**.

Identification code	**2**	
Empirical formula	$C_{39} H_{28} Au_2 F_8 P_2 S_2$	
Formula weight	1168.61	
Temperature	130(2) K	
Wavelength	0.71073 Å	
Crystal system	Monoclinic	
Space group	C 2/c	
Unit cell dimensions	a = 19.7541(14) Å	α= 90°.
	b = 18.1508(14) Å	β= 113.410(8)°.
	c = 23.2826(17) Å	γ = 90°.
Volume	7660.9(11) $Å^3$	
Z	8	
Density (calculated)	2.026 Mg/m^3	
Absorption coefficient	7.912 mm^{-1}	
F(000)	4432	
Crystal size	0.470 x 0.210 x 0.200 mm^3	
Theta range for data collection	3.550 to 29.534°.	
Index ranges	-27<=h<=24, -23<=k<=24, -31<=l<=29	
Reflections collected	25955	
Independent reflections	9266 [R(int) = 0.0335]	
Completeness to theta = 25.242°	99.7 %	
Refinement method	Full-matrix least-squares on F^2	
Data / restraints / parameters	9266 / 0 / 478	
Goodness-of-fit on F^2	1.055	
Final R indices [I>2sigma(I)]	R1 = 0.0282, wR2 = 0.0534	
R indices (all data)	R1 = 0.0390, wR2 = 0.0578	
Largest diff. peak and hole	0.943 and -1.299 e.$Å^{-3}$	

Table S5. Atomic coordinates (x 10^4) and equivalent isotropic displacement parameters ($Å^2$x 10^3) for **2**. U(eq) is defined as one third of the trace of the orthogonalized U^{ij} tensor.

	x	y	z	U(eq)
C(1)	2665(2)	387(2)	3730(2)	20(1)
C(2)	3071(2)	1031(2)	3905(2)	21(1)
C(3)	3774(2)	1045(3)	4375(2)	28(1)
C(4)	4102(2)	422(3)	4695(2)	35(1)
C(5)	3707(3)	-223(3)	4529(2)	39(1)
C(6)	3008(2)	-241(2)	4062(2)	26(1)
C(7)	1313(2)	1870(2)	944(2)	18(1)
C(8)	1964(2)	1644(2)	906(2)	23(1)
C(9)	1973(2)	1448(2)	334(2)	27(1)
C(10)	1330(3)	1448(2)	-199(2)	29(1)
C(11)	677(2)	1657(2)	-164(2)	27(1)
C(12)	662(2)	1875(2)	400(2)	22(1)
C(13)	448(2)	2532(2)	1571(2)	17(1)
C(14)	-76(2)	2120(2)	1686(2)	20(1)
C(15)	-754(2)	2425(3)	1597(2)	28(1)
C(16)	-919(3)	3137(3)	1390(2)	36(1)
C(17)	-403(3)	3552(3)	1275(2)	40(1)
C(18)	280(3)	3254(2)	1358(2)	32(1)
C(19)	2038(2)	2822(2)	1953(2)	21(1)
C(20)	2188(2)	3217(2)	2572(2)	18(1)
C(21)	2954(2)	3578(2)	2792(2)	16(1)
C(22)	1027(2)	6362(2)	2903(2)	20(1)
C(23)	797(2)	5817(2)	3207(2)	23(1)
C(24)	441(2)	5981(2)	3592(2)	24(1)
C(25)	284(2)	6694(2)	3694(2)	26(1)
C(26)	495(2)	7238(2)	3393(2)	25(1)
C(27)	865(2)	7082(2)	3021(2)	21(1)
C(28)	4047(2)	4610(2)	3567(2)	13(1)
C(29)	4362(2)	5066(2)	4090(2)	17(1)
C(30)	5075(2)	5323(2)	4259(2)	19(1)
C(31)	5474(2)	5138(2)	3909(2)	20(1)
C(32)	5164(2)	4685(2)	3393(2)	19(1)
C(33)	4457(2)	4417(2)	3223(2)	16(1)
C(34)	3021(2)	3995(2)	4027(2)	15(1)
C(35)	2610(2)	4377(2)	4293(2)	22(1)
C(36)	2569(2)	4127(3)	4842(2)	28(1)
C(37)	2937(2)	3500(2)	5126(2)	26(1)

C(38)	3346(2)	3113(2)	4868(2)	28(1)
C(39)	3388(2)	3360(2)	4320(2)	23(1)
Au(1)	1598(1)	1200(1)	2418(1)	15(1)
Au(2)	2299(1)	5279(1)	2872(1)	15(1)
F(1)	2783(1)	1676(1)	3634(1)	27(1)
F(2)	4137(1)	1694(2)	4515(1)	36(1)
F(3)	4006(2)	-858(2)	4820(1)	63(1)
F(4)	2646(2)	-890(1)	3934(1)	41(1)
F(5)	904(1)	5100(1)	3117(1)	32(1)
F(6)	228(2)	5428(2)	3866(1)	38(1)
F(7)	334(2)	7951(1)	3451(1)	34(1)
F(8)	1060(1)	7649(1)	2739(1)	31(1)
P(1)	1328(1)	2116(1)	1704(1)	15(1)
P(2)	3088(1)	4348(1)	3325(1)	12(1)
S(1)	1760(1)	304(1)	3167(1)	22(1)
S(2)	1467(1)	6206(1)	2392(1)	26(1)

Table S6. Bond lengths [Å] and angles [°] for **2**.

C(1)-C(2)	1.384(6)
C(1)-C(6)	1.393(6)
C(1)-S(1)	1.754(4)
C(2)-F(1)	1.344(5)
C(2)-C(3)	1.385(6)
C(3)-F(2)	1.350(5)
C(3)-C(4)	1.368(7)
C(4)-C(5)	1.374(7)
C(4)-H(4)	0.9500
C(5)-F(3)	1.349(5)
C(5)-C(6)	1.377(6)
C(6)-F(4)	1.349(5)
C(7)-C(8)	1.387(6)
C(7)-C(12)	1.401(5)
C(7)-P(1)	1.814(4)
C(8)-C(9)	1.385(6)
C(8)-H(8)	0.9500
C(9)-C(10)	1.379(6)
C(9)-H(9)	0.9500
C(10)-C(11)	1.378(6)
C(10)-H(10)	0.9500
C(11)-C(12)	1.381(6)
C(11)-H(11)	0.9500
C(12)-H(12)	0.9500
C(13)-C(14)	1.387(6)
C(13)-C(18)	1.393(6)
C(13)-P(1)	1.804(4)
C(14)-C(15)	1.387(6)
C(14)-H(14)	0.9500
C(15)-C(16)	1.374(7)
C(15)-H(15)	0.9500
C(16)-C(17)	1.375(7)
C(16)-H(16)	0.9500
C(17)-C(18)	1.394(7)
C(17)-H(17)	0.9500
C(18)-H(18)	0.9500
C(19)-C(20)	1.529(5)
C(19)-P(1)	1.817(4)
C(19)-H(19A)	0.9900
C(19)-H(19B)	0.9900
C(20)-C(21)	1.538(5)
C(20)-H(20A)	0.9900
C(20)-H(20B)	0.9900
C(21)-P(2)	1.817(4)
C(21)-H(21A)	0.9900
C(21)-H(21B)	0.9900
C(22)-C(23)	1.393(6)
C(22)-C(27)	1.398(6)
C(22)-S(2)	1.754(4)
C(23)-F(5)	1.347(5)
C(23)-C(24)	1.374(6)
C(24)-F(6)	1.342(5)
C(24)-C(25)	1.373(6)
C(25)-C(26)	1.367(6)
C(25)-H(25)	0.9500
C(26)-F(7)	1.352(5)
C(26)-C(27)	1.368(6)
C(27)-F(8)	1.355(5)
C(28)-C(33)	1.391(5)
C(28)-C(29)	1.396(5)
C(28)-P(2)	1.812(4)
C(29)-C(30)	1.384(5)
C(29)-H(29)	0.9500
C(30)-C(31)	1.381(6)
C(30)-H(30)	0.9500
C(31)-C(32)	1.384(6)
C(31)-H(31)	0.9500
C(32)-C(33)	1.380(5)
C(32)-H(32)	0.9500
C(33)-H(33)	0.9500
C(34)-C(39)	1.386(5)
C(34)-C(35)	1.389(6)
C(34)-P(2)	1.808(4)
C(35)-C(36)	1.387(6)
C(35)-H(35)	0.9500
C(36)-C(37)	1.371(6)
C(36)-H(36)	0.9500
C(37)-C(38)	1.377(6)
C(37)-H(37)	0.9500
C(38)-C(39)	1.387(6)
C(38)-H(38)	0.9500
C(39)-H(39)	0.9500
Au(1)-P(1)	2.2589(10)
Au(1)-S(1)	2.3131(10)
Au(1)-Au(2)#1	3.0287(3)
Au(2)-P(2)	2.2569(10)
Au(2)-S(2)	2.3077(10)
C(2)-C(1)-C(6)	115.5(4)
C(2)-C(1)-S(1)	126.3(3)

C(6)-C(1)-S(1)	118.1(3)
F(1)-C(2)-C(1)	120.6(3)
F(1)-C(2)-C(3)	117.3(4)
C(1)-C(2)-C(3)	122.0(4)
F(2)-C(3)-C(4)	119.9(4)
F(2)-C(3)-C(2)	118.5(4)
C(4)-C(3)-C(2)	121.6(4)
C(3)-C(4)-C(5)	117.3(4)
C(3)-C(4)-H(4)	121.4
C(5)-C(4)-H(4)	121.4
F(3)-C(5)-C(4)	120.1(4)
F(3)-C(5)-C(6)	118.5(5)
C(4)-C(5)-C(6)	121.4(4)
F(4)-C(6)-C(5)	118.2(4)
F(4)-C(6)-C(1)	119.6(4)
C(5)-C(6)-C(1)	122.2(4)
C(8)-C(7)-C(12)	118.9(4)
C(8)-C(7)-P(1)	118.7(3)
C(12)-C(7)-P(1)	122.3(3)
C(9)-C(8)-C(7)	120.3(4)
C(9)-C(8)-H(8)	119.8
C(7)-C(8)-H(8)	119.8
C(10)-C(9)-C(8)	120.4(4)
C(10)-C(9)-H(9)	119.8
C(8)-C(9)-H(9)	119.8
C(11)-C(10)-C(9)	119.7(4)
C(11)-C(10)-H(10)	120.1
C(9)-C(10)-H(10)	120.1
C(10)-C(11)-C(12)	120.6(4)
C(10)-C(11)-H(11)	119.7
C(12)-C(11)-H(11)	119.7
C(11)-C(12)-C(7)	120.0(4)
C(11)-C(12)-H(12)	120.0
C(7)-C(12)-H(12)	120.0
C(14)-C(13)-C(18)	119.0(4)
C(14)-C(13)-P(1)	118.9(3)
C(18)-C(13)-P(1)	122.1(3)
C(13)-C(14)-C(15)	120.5(4)
C(13)-C(14)-H(14)	119.8
C(15)-C(14)-H(14)	119.8
C(16)-C(15)-C(14)	120.5(4)
C(16)-C(15)-H(15)	119.8
C(14)-C(15)-H(15)	119.8
C(15)-C(16)-C(17)	119.6(4)
C(15)-C(16)-H(16)	120.2
C(17)-C(16)-H(16)	120.2
C(16)-C(17)-C(18)	120.7(4)
C(16)-C(17)-H(17)	119.7

C(18)-C(17)-H(17)	119.7
C(17)-C(18)-C(13)	119.8(4)
C(17)-C(18)-H(18)	120.1
C(13)-C(18)-H(18)	120.1
C(20)-C(19)-P(1)	117.8(3)
C(20)-C(19)-H(19A)	107.8
P(1)-C(19)-H(19A)	107.8
C(20)-C(19)-H(19B)	107.8
P(1)-C(19)-H(19B)	107.8
H(19A)-C(19)-H(19B)	107.2
C(19)-C(20)-C(21)	108.1(3)
C(19)-C(20)-H(20A)	110.1
C(21)-C(20)-H(20A)	110.1
C(19)-C(20)-H(20B)	110.1
C(21)-C(20)-H(20B)	110.1
H(20A)-C(20)-H(20B)	108.4
C(20)-C(21)-P(2)	114.4(3)
C(20)-C(21)-H(21A)	108.7
P(2)-C(21)-H(21A)	108.7
C(20)-C(21)-H(21B)	108.7
P(2)-C(21)-H(21B)	108.7
H(21A)-C(21)-H(21B)	107.6
C(23)-C(22)-C(27)	114.8(4)
C(23)-C(22)-S(2)	125.4(3)
C(27)-C(22)-S(2)	119.8(3)
F(5)-C(23)-C(24)	117.6(4)
F(5)-C(23)-C(22)	120.3(4)
C(24)-C(23)-C(22)	122.1(4)
F(6)-C(24)-C(25)	119.2(4)
F(6)-C(24)-C(23)	119.0(4)
C(25)-C(24)-C(23)	121.7(4)
C(26)-C(25)-C(24)	117.2(4)
C(26)-C(25)-H(25)	121.4
C(24)-C(25)-H(25)	121.4
F(7)-C(26)-C(27)	117.8(4)
F(7)-C(26)-C(25)	120.5(4)
C(27)-C(26)-C(25)	121.6(4)
F(8)-C(27)-C(26)	118.5(4)
F(8)-C(27)-C(22)	119.0(4)
C(26)-C(27)-C(22)	122.5(4)
C(33)-C(28)-C(29)	119.4(3)
C(33)-C(28)-P(2)	122.0(3)
C(29)-C(28)-P(2)	118.4(3)
C(30)-C(29)-C(28)	119.9(4)
C(30)-C(29)-H(29)	120.0
C(28)-C(29)-H(29)	120.0
C(31)-C(30)-C(29)	120.3(4)
C(31)-C(30)-H(30)	119.8

C(29)-C(30)-H(30)	119.8
C(30)-C(31)-C(32)	119.8(4)
C(30)-C(31)-H(31)	120.1
C(32)-C(31)-H(31)	120.1
C(33)-C(32)-C(31)	120.4(4)
C(33)-C(32)-H(32)	119.8
C(31)-C(32)-H(32)	119.8
C(32)-C(33)-C(28)	120.1(3)
C(32)-C(33)-H(33)	120.0
C(28)-C(33)-H(33)	120.0
C(39)-C(34)-C(35)	118.7(4)
C(39)-C(34)-P(2)	121.8(3)
C(35)-C(34)-P(2)	119.5(3)
C(36)-C(35)-C(34)	120.4(4)
C(36)-C(35)-H(35)	119.8
C(34)-C(35)-H(35)	119.8
C(37)-C(36)-C(35)	120.2(4)
C(37)-C(36)-H(36)	119.9
C(35)-C(36)-H(36)	119.9
C(36)-C(37)-C(38)	120.3(4)
C(36)-C(37)-H(37)	119.9
C(38)-C(37)-H(37)	119.9
C(37)-C(38)-C(39)	119.7(4)
C(37)-C(38)-H(38)	120.1

C(39)-C(38)-H(38)	120.1
C(34)-C(39)-C(38)	120.7(4)
C(34)-C(39)-H(39)	119.6
C(38)-C(39)-H(39)	119.6
P(1)-Au(1)-S(1)	173.63(4)
P(1)-Au(1)-Au(2)#1	102.91(3)
S(1)-Au(1)-Au(2)#1	83.26(3)
P(2)-Au(2)-S(2)	178.40(4)
P(2)-Au(2)-Au(1)#2	95.33(3)
S(2)-Au(2)-Au(1)#2	85.76(3)
C(13)-P(1)-C(7)	107.07(18)
C(13)-P(1)-C(19)	109.19(19)
C(7)-P(1)-C(19)	101.31(18)
C(13)-P(1)-Au(1)	111.37(13)
C(7)-P(1)-Au(1)	116.32(13)
C(19)-P(1)-Au(1)	110.97(14)
C(34)-P(2)-C(28)	104.79(16)
C(34)-P(2)-C(21)	107.56(18)
C(28)-P(2)-C(21)	105.41(17)
C(34)-P(2)-Au(2)	113.77(13)
C(28)-P(2)-Au(2)	113.04(12)
C(21)-P(2)-Au(2)	111.66(13)
C(1)-S(1)-Au(1)	107.32(14)
C(22)-S(2)-Au(2)	103.80(13)

Table S7. Crystal data and structure refinement for **4**.

Identification code	**4**	
Empirical formula	$C_{41} H_{34} Au_2 F_6 P_2 S_2$	
Formula weight	1160.67	
Temperature	130(2) K	
Wavelength	0.71073 Å	
Crystal system	Triclinic	
Space group	P -1	
Unit cell dimensions	a = 8.8464(3) Å	α= 98.173(4)°.
	b = 10.8946(6) Å	β= 99.266(3)°.
	c = 21.8504(8) Å	γ = 104.869(4)°.
Volume	1971.31(15) $Å^3$	
Z	2	
Density (calculated)	1.955 Mg/m^3	
Absorption coefficient	7.679 mm^{-1}	
F(000)	1108	
Crystal size	0.400 x 0.130 x 0.080 mm^3	
Theta range for data collection	3.545 to 29.573°.	
Index ranges	-11<=h<=12, -11<=k<=13, -30<=l<=28	
Reflections collected	17616	
Independent reflections	9220 [R(int) = 0.0278]	
Completeness to theta = 25.242°	99.7 %	
Refinement method	Full-matrix least-squares on F^2	
Data / restraints / parameters	9220 / 0 / 478	
Goodness-of-fit on F^2	1.030	
Final R indices [I>2sigma(I)]	R1 = 0.0291, wR2 = 0.0525	
R indices (all data)	R1 = 0.0390, wR2 = 0.0571	
Largest diff. peak and hole	1.335 and -1.466 e.$Å^{-3}$	

Table S8. Atomic coordinates (x 10^4) and equivalent isotropic displacement parameters ($Å^2$x 10^3) for **4**. U(eq) is defined as one third of the trace of the orthogonalized U^{ij} tensor.

	x	y	z	U(eq)
C(1)	420(4)	-1656(4)	3979(2)	20(1)
C(2)	990(4)	-1589(4)	4625(2)	24(1)
C(3)	751(6)	-2765(5)	4912(2)	34(1)
C(4)	1799(5)	-394(5)	5018(2)	32(1)
C(5)	2047(5)	729(5)	4787(2)	35(1)
C(6)	1464(5)	677(5)	4151(2)	32(1)
C(7)	653(5)	-498(4)	3761(2)	27(1)
C(8)	1646(4)	-1056(4)	1500(2)	18(1)
C(9)	2635(5)	-284(4)	2057(2)	27(1)
C(10)	3724(5)	865(5)	2026(3)	40(1)
C(11)	3858(5)	1224(5)	1457(3)	39(1)
C(12)	2902(5)	462(5)	909(2)	37(1)
C(13)	1789(5)	-678(4)	929(2)	27(1)
C(14)	-1557(4)	-2658(4)	958(2)	19(1)
C(15)	-2123(4)	-3532(4)	384(2)	24(1)
C(16)	-3544(5)	-3545(5)	-4(2)	34(1)
C(17)	-4383(5)	-2716(5)	175(2)	36(1)
C(18)	-3832(5)	-1837(5)	743(2)	33(1)
C(19)	-2412(5)	-1814(4)	1129(2)	26(1)
C(20)	1135(4)	-3773(4)	1150(2)	18(1)
C(21)	2731(4)	-3758(4)	1553(2)	19(1)
C(22)	-1576(4)	-6745(4)	1819(2)	18(1)
C(23)	-939(4)	-7659(4)	1517(2)	20(1)
C(24)	-1222(5)	-8013(4)	813(2)	27(1)
C(25)	-40(5)	-8300(4)	1868(2)	26(1)
C(26)	213(5)	-8065(4)	2523(2)	29(1)
C(27)	-468(5)	-7203(4)	2819(2)	27(1)
C(28)	-1340(4)	-6551(4)	2477(2)	21(1)
C(29)	-5683(4)	-4928(4)	3283(2)	22(1)
C(30)	-4439(5)	-5371(5)	3544(2)	31(1)
C(31)	-4527(5)	-5955(5)	4067(2)	37(1)
C(32)	-5855(5)	-6096(5)	4324(2)	34(1)
C(33)	-7088(5)	-5663(5)	4069(2)	32(1)
C(34)	-7023(5)	-5085(4)	3545(2)	28(1)
C(35)	-4895(4)	-2386(4)	2955(2)	22(1)
C(36)	-5640(5)	-1903(5)	3410(2)	30(1)
C(37)	-5272(6)	-599(5)	3636(2)	39(1)

C(38)	-4158(6)	245(5)	3407(2)	41(1)
C(39)	-3405(5)	-213(5)	2963(3)	47(2)
C(40)	-3757(5)	-1521(4)	2740(2)	31(1)
C(41)	-7396(4)	-4408(4)	2121(2)	20(1)
Au(1)	-239(1)	-2846(1)	2478(1)	17(1)
Au(2)	-3803(1)	-4811(1)	2051(1)	18(1)
F(1)	1208(4)	-2497(3)	5543(1)	52(1)
F(2)	1562(4)	-3561(3)	4706(1)	52(1)
F(3)	-791(3)	-3484(3)	4794(1)	46(1)
F(4)	-2777(3)	-8497(3)	543(1)	36(1)
F(5)	-532(3)	-8912(3)	619(1)	42(1)
F(6)	-656(3)	-7004(3)	536(1)	39(1)
P(1)	240(1)	-2597(1)	1512(1)	15(1)
P(2)	-5428(1)	-4103(1)	2624(1)	18(1)
S(1)	-565(1)	-3161(1)	3472(1)	26(1)
S(2)	-2580(1)	-5848(1)	1374(1)	20(1)

Table S9. Bond lengths [Å] and angles [°] for **4**.

C(1)-C(7)	1.389(6)	C(20)-C(21)	1.533(5)
C(1)-C(2)	1.406(5)	C(20)-P(1)	1.824(4)
C(1)-S(1)	1.764(4)	C(20)-H(20A)	0.9900
C(2)-C(4)	1.396(6)	C(20)-H(20B)	0.9900
C(2)-C(3)	1.486(7)	C(21)-C(41)#1	1.519(5)
C(3)-F(2)	1.335(6)	C(21)-H(21A)	0.9900
C(3)-F(1)	1.340(5)	C(21)-H(21B)	0.9900
C(3)-F(3)	1.350(5)	C(22)-C(28)	1.396(5)
C(4)-C(5)	1.369(7)	C(22)-C(23)	1.402(6)
C(4)-H(4)	0.9500	C(22)-S(2)	1.767(4)
C(5)-C(6)	1.391(6)	C(23)-C(25)	1.396(6)
C(5)-H(5)	0.9500	C(23)-C(24)	1.495(5)
C(6)-C(7)	1.379(6)	C(24)-F(5)	1.336(5)
C(6)-H(6)	0.9500	C(24)-F(4)	1.341(5)
C(7)-H(7)	0.9500	C(24)-F(6)	1.353(5)
C(8)-C(13)	1.382(6)	C(25)-C(26)	1.388(6)
C(8)-C(9)	1.393(5)	C(25)-H(25)	0.9500
C(8)-P(1)	1.822(4)	C(26)-C(27)	1.380(6)
C(9)-C(10)	1.387(6)	C(26)-H(26)	0.9500
C(9)-H(9)	0.9500	C(27)-C(28)	1.380(6)
C(10)-C(11)	1.371(7)	C(27)-H(27)	0.9500
C(10)-H(10)	0.9500	C(28)-H(28)	0.9500
C(11)-C(12)	1.367(6)	C(29)-C(34)	1.381(6)
C(11)-H(11)	0.9500	C(29)-C(30)	1.385(6)
C(12)-C(13)	1.384(6)	C(29)-P(2)	1.816(4)
C(12)-H(12)	0.9500	C(30)-C(31)	1.387(6)
C(13)-H(13)	0.9500	C(30)-H(30)	0.9500
C(14)-C(19)	1.381(6)	C(31)-C(32)	1.365(6)
C(14)-C(15)	1.394(5)	C(31)-H(31)	0.9500
C(14)-P(1)	1.816(4)	C(32)-C(33)	1.366(7)
C(15)-C(16)	1.395(6)	C(32)-H(32)	0.9500
C(15)-H(15)	0.9500	C(33)-C(34)	1.384(6)
C(16)-C(17)	1.364(7)	C(33)-H(33)	0.9500
C(16)-H(16)	0.9500	C(34)-H(34)	0.9500
C(17)-C(18)	1.386(7)	C(35)-C(40)	1.383(6)
C(17)-H(17)	0.9500	C(35)-C(36)	1.393(6)
C(18)-C(19)	1.388(6)	C(35)-P(2)	1.816(4)
C(18)-H(18)	0.9500	C(36)-C(37)	1.371(6)
C(19)-H(19)	0.9500	C(36)-H(36)	0.9500

C(37)-C(38)	1.376(7)
C(37)-H(37)	0.9500
C(38)-C(39)	1.370(7)
C(38)-H(38)	0.9500
C(39)-C(40)	1.375(7)
C(39)-H(39)	0.9500
C(40)-H(40)	0.9500
C(41)-C(21)#2	1.519(5)
C(41)-P(2)	1.826(4)
C(41)-H(41A)	0.9900
C(41)-H(41B)	0.9900
Au(1)-P(1)	2.2583(10)
Au(1)-S(1)	2.2985(10)
Au(1)-Au(2)	3.2276(3)
Au(2)-P(2)	2.2595(10)
Au(2)-S(2)	2.3026(10)
C(7)-C(1)-C(2)	117.4(4)
C(7)-C(1)-S(1)	121.9(3)
C(2)-C(1)-S(1)	120.7(3)
C(4)-C(2)-C(1)	120.1(4)
C(4)-C(2)-C(3)	118.1(4)
C(1)-C(2)-C(3)	121.8(4)
F(2)-C(3)-F(1)	105.8(4)
F(2)-C(3)-F(3)	105.9(4)
F(1)-C(3)-F(3)	104.9(4)
F(2)-C(3)-C(2)	113.0(4)
F(1)-C(3)-C(2)	113.2(4)
F(3)-C(3)-C(2)	113.2(4)
C(5)-C(4)-C(2)	121.3(4)
C(5)-C(4)-H(4)	119.4
C(2)-C(4)-H(4)	119.4
C(4)-C(5)-C(6)	119.2(4)
C(4)-C(5)-H(5)	120.4
C(6)-C(5)-H(5)	120.4
C(7)-C(6)-C(5)	119.9(5)
C(7)-C(6)-H(6)	120.1
C(5)-C(6)-H(6)	120.1
C(6)-C(7)-C(1)	122.1(4)
C(6)-C(7)-H(7)	118.9
C(1)-C(7)-H(7)	118.9
C(13)-C(8)-C(9)	119.6(4)

C(13)-C(8)-P(1)	119.6(3)
C(9)-C(8)-P(1)	120.7(3)
C(10)-C(9)-C(8)	119.1(4)
C(10)-C(9)-H(9)	120.5
C(8)-C(9)-H(9)	120.5
C(11)-C(10)-C(9)	120.7(4)
C(11)-C(10)-H(10)	119.7
C(9)-C(10)-H(10)	119.7
C(12)-C(11)-C(10)	120.4(4)
C(12)-C(11)-H(11)	119.8
C(10)-C(11)-H(11)	119.8
C(11)-C(12)-C(13)	119.8(5)
C(11)-C(12)-H(12)	120.1
C(13)-C(12)-H(12)	120.1
C(8)-C(13)-C(12)	120.4(4)
C(8)-C(13)-H(13)	119.8
C(12)-C(13)-H(13)	119.8
C(19)-C(14)-C(15)	119.2(4)
C(19)-C(14)-P(1)	117.6(3)
C(15)-C(14)-P(1)	123.2(3)
C(14)-C(15)-C(16)	119.6(4)
C(14)-C(15)-H(15)	120.2
C(16)-C(15)-H(15)	120.2
C(17)-C(16)-C(15)	120.4(4)
C(17)-C(16)-H(16)	119.8
C(15)-C(16)-H(16)	119.8
C(16)-C(17)-C(18)	120.7(4)
C(16)-C(17)-H(17)	119.7
C(18)-C(17)-H(17)	119.7
C(17)-C(18)-C(19)	119.0(5)
C(17)-C(18)-H(18)	120.5
C(19)-C(18)-H(18)	120.5
C(14)-C(19)-C(18)	121.1(4)
C(14)-C(19)-H(19)	119.5
C(18)-C(19)-H(19)	119.5
C(21)-C(20)-P(1)	113.0(3)
C(21)-C(20)-H(20A)	109.0
P(1)-C(20)-H(20A)	109.0
C(21)-C(20)-H(20B)	109.0
P(1)-C(20)-H(20B)	109.0
H(20A)-C(20)-H(20B)	107.8
C(41)#1-C(21)-C(20)	115.4(3)

C(41)#1-C(21)-H(21A)	108.4
C(20)-C(21)-H(21A)	108.4
C(41)#1-C(21)-H(21B)	108.4
C(20)-C(21)-H(21B)	108.4
H(21A)-C(21)-H(21B)	107.5
C(28)-C(22)-C(23)	117.5(4)
C(28)-C(22)-S(2)	122.1(3)
C(23)-C(22)-S(2)	120.5(3)
C(25)-C(23)-C(22)	120.8(4)
C(25)-C(23)-C(24)	117.4(4)
C(22)-C(23)-C(24)	121.9(4)
F(5)-C(24)-F(4)	105.7(3)
F(5)-C(24)-F(6)	105.6(4)
F(4)-C(24)-F(6)	105.8(3)
F(5)-C(24)-C(23)	112.7(4)
F(4)-C(24)-C(23)	112.7(4)
F(6)-C(24)-C(23)	113.6(3)
C(26)-C(25)-C(23)	120.6(4)
C(26)-C(25)-H(25)	119.7
C(23)-C(25)-H(25)	119.7
C(27)-C(26)-C(25)	118.6(4)
C(27)-C(26)-H(26)	120.7
C(25)-C(26)-H(26)	120.7
C(28)-C(27)-C(26)	121.2(4)
C(28)-C(27)-H(27)	119.4
C(26)-C(27)-H(27)	119.4
C(27)-C(28)-C(22)	121.3(4)
C(27)-C(28)-H(28)	119.4
C(22)-C(28)-H(28)	119.4
C(34)-C(29)-C(30)	119.2(4)
C(34)-C(29)-P(2)	123.0(3)
C(30)-C(29)-P(2)	117.7(3)
C(29)-C(30)-C(31)	120.6(4)
C(29)-C(30)-H(30)	119.7
C(31)-C(30)-H(30)	119.7
C(32)-C(31)-C(30)	119.6(4)
C(32)-C(31)-H(31)	120.2
C(30)-C(31)-H(31)	120.2
C(31)-C(32)-C(33)	120.2(4)
C(31)-C(32)-H(32)	119.9
C(33)-C(32)-H(32)	119.9
C(32)-C(33)-C(34)	120.9(4)

C(32)-C(33)-H(33)	119.5
C(34)-C(33)-H(33)	119.5
C(29)-C(34)-C(33)	119.5(4)
C(29)-C(34)-H(34)	120.3
C(33)-C(34)-H(34)	120.3
C(40)-C(35)-C(36)	118.6(4)
C(40)-C(35)-P(2)	119.8(3)
C(36)-C(35)-P(2)	121.6(3)
C(37)-C(36)-C(35)	120.9(5)
C(37)-C(36)-H(36)	119.6
C(35)-C(36)-H(36)	119.6
C(36)-C(37)-C(38)	119.5(5)
C(36)-C(37)-H(37)	120.2
C(38)-C(37)-H(37)	120.2
C(39)-C(38)-C(37)	120.3(5)
C(39)-C(38)-H(38)	119.8
C(37)-C(38)-H(38)	119.8
C(38)-C(39)-C(40)	120.3(5)
C(38)-C(39)-H(39)	119.8
C(40)-C(39)-H(39)	119.8
C(39)-C(40)-C(35)	120.3(5)
C(39)-C(40)-H(40)	119.9
C(35)-C(40)-H(40)	119.9
C(21)#2-C(41)-P(2)	110.8(2)
C(21)#2-C(41)-H(41A)	109.5
P(2)-C(41)-H(41A)	109.5
C(21)#2-C(41)-H(41B)	109.5
P(2)-C(41)-H(41B)	109.5
H(41A)-C(41)-H(41B)	108.1
P(1)-Au(1)-S(1)	175.75(4)
P(1)-Au(1)-Au(2)	98.75(2)
S(1)-Au(1)-Au(2)	83.37(2)
P(2)-Au(2)-S(2)	169.04(3)
P(2)-Au(2)-Au(1)	109.65(3)
S(2)-Au(2)-Au(1)	81.31(2)
C(14)-P(1)-C(8)	104.50(18)
C(14)-P(1)-C(20)	107.61(18)
C(8)-P(1)-C(20)	102.71(18)
C(14)-P(1)-Au(1)	112.20(13)
C(8)-P(1)-Au(1)	114.39(13)
C(20)-P(1)-Au(1)	114.50(13)
C(29)-P(2)-C(35)	105.91(19)

C(29)-P(2)-C(41)	107.97(17)
C(35)-P(2)-C(41)	103.06(19)
C(29)-P(2)-Au(2)	111.85(15)
C(35)-P(2)-Au(2)	118.00(14)
C(41)-P(2)-Au(2)	109.34(14)
C(1)-S(1)-Au(1)	105.49(14)
C(22)-S(2)-Au(2)	106.47(14)

Table S10. Crystal data and structure refinement for **5**.

Identification code	**5**	
Empirical formula	$C_{43} H_{36} Au_2 Cl_6 F_6 P_2 S_2$	
Formula weight	1399.41	
Temperature	130(2) K	
Wavelength	0.71073 Å	
Crystal system	Monoclinic	
Space group	P 21/c	
Unit cell dimensions	a = 16.9549(6) Å	α= 90°.
	b = 19.0738(6) Å	β= 97.626(3)°.
	c = 14.7274(5) Å	γ = 90°.
Volume	4720.6(3) $Å^3$	
Z	4	
Density (calculated)	1.969 Mg/m^3	
Absorption coefficient	6.760 mm^{-1}	
F(000)	2680	
Crystal size	0.370 x 0.270 x 0.120 mm^3	
Theta range for data collection	3.426 to 29.421°.	
Index ranges	-23<=h<=23, -25<=k<=18, -11<=l<=20	
Reflections collected	23644	
Independent reflections	11145 [R(int) = 0.0338]	
Completeness to theta = 25.242°	99.7 %	
Refinement method	Full-matrix least-squares on F^2	
Data / restraints / parameters	11145 / 0 / 550	
Goodness-of-fit on F^2	1.051	
Final R indices [I>2sigma(I)]	R1 = 0.0378, wR2 = 0.0827	
R indices (all data)	R1 = 0.0545, wR2 = 0.0918	
Largest diff. peak and hole	1.581 and -1.931 e.$Å^{-3}$	

Table S11. Atomic coordinates (x 10^4) and equivalent isotropic displacement parameters ($Å^2$x 10^3) for **5**. U(eq) is defined as one third of the trace of the orthogonalized U^{ij} tensor.

	x	y	z	U(eq)
Au(1)	8670(1)	3546(1)	257(1)	18(1)
Au(2)	9392(1)	2955(1)	4830(1)	16(1)
S(1)	8573(1)	3736(1)	-1298(1)	21(1)
S(2)	8534(1)	3618(1)	5561(1)	22(1)
P(2)	10461(1)	2500(1)	4256(1)	16(1)
P(1)	8589(1)	3496(1)	1774(1)	17(1)
F(1)	4707(3)	4567(3)	-2239(4)	67(1)
F(4)	4799(3)	2881(3)	3668(4)	70(2)
F(5)	4628(3)	3325(3)	4944(4)	77(2)
F(2)	4725(3)	3565(2)	-2862(4)	67(2)
F(3)	5000(3)	4463(3)	-3598(4)	70(2)
F(6)	4707(3)	3990(3)	3808(5)	94(2)
C(20)	9027(3)	2748(3)	2410(4)	17(1)
C(37)	11049(4)	3855(3)	4117(4)	21(1)
C(30)	10912(3)	1798(3)	4971(4)	18(1)
C(36)	11242(3)	3145(3)	4201(4)	18(1)
C(23)	7530(3)	3522(3)	5065(4)	18(1)
C(31)	10791(4)	1098(3)	4739(4)	25(1)
C(21)	9930(3)	2690(3)	2387(4)	18(1)
C(22)	10315(3)	2150(3)	3088(4)	18(1)
C(2)	6953(4)	3843(3)	-1117(4)	26(1)
C(24)	7108(4)	2904(3)	5131(4)	28(1)
C(9)	7042(4)	3983(3)	1508(4)	25(1)
C(8)	7544(3)	3467(3)	1927(4)	19(1)
C(41)	12019(4)	2933(3)	4158(4)	26(1)
C(14)	9006(3)	4254(3)	2415(4)	19(1)
C(15)	8766(4)	4410(3)	3262(4)	23(1)
C(1)	7548(3)	3884(3)	-1680(4)	20(1)
C(35)	11337(4)	1960(3)	5826(4)	24(1)
C(29)	7122(4)	4103(3)	4689(4)	24(1)
C(39)	12403(4)	4128(3)	3955(4)	28(1)
C(17)	9707(4)	5353(3)	3452(4)	29(1)
C(19)	9609(4)	4642(3)	2096(4)	22(1)
C(32)	11092(4)	573(3)	5342(5)	33(2)
C(7)	7338(4)	4035(3)	-2596(4)	26(1)
C(16)	9125(4)	4969(3)	3772(4)	28(1)
C(13)	7226(4)	2924(3)	2380(4)	27(1)

C(34)	11637(4)	1435(3)	6419(4)	30(1)
C(38)	11625(4)	4343(3)	3983(4)	28(1)
C(25)	6297(4)	2878(4)	4855(5)	35(2)
C(4)	5955(4)	4093(3)	-2395(4)	29(1)
C(26)	5898(4)	3462(3)	4492(4)	30(1)
C(6)	6548(4)	4135(3)	-2953(4)	31(1)
C(3)	6159(4)	3950(4)	-1467(5)	34(2)
C(40)	12601(4)	3433(3)	4049(5)	34(2)
C(33)	11513(4)	736(3)	6179(4)	30(1)
C(28)	6303(4)	4083(3)	4400(4)	29(1)
C(10)	6232(4)	3948(4)	1533(5)	36(2)
C(18)	9960(4)	5195(3)	2618(4)	25(1)
C(11)	5909(4)	3398(4)	1968(5)	38(2)
C(27)	5017(5)	3423(4)	4237(5)	47(2)
C(5)	5104(5)	4170(4)	-2766(5)	42(2)
C(12)	6403(4)	2885(3)	2398(5)	33(2)
Cl(1)	3714(2)	4111(1)	1743(2)	68(1)
Cl(2)	3721(2)	3027(2)	409(2)	100(1)
Cl(3)	4078(2)	4435(2)	-59(2)	95(1)
C(42)	3516(5)	3906(4)	566(6)	54(2)
Cl(4)	2168(1)	939(1)	3201(2)	58(1)
Cl(5)	3036(2)	2104(1)	2554(3)	103(1)
Cl(6)	2693(2)	951(2)	1394(2)	117(1)
C(43)	2909(5)	1193(4)	2536(6)	49(2)

Table S12. Bond lengths [Å] and angles [°] for **5**.

Au(1)-P(1)	2.2586(13)
Au(1)-S(1)	2.3025(13)
Au(1)-Au(2)#1	3.2070(3)
Au(2)-P(2)	2.2718(14)
Au(2)-S(2)	2.3011(14)
S(1)-C(1)	1.777(6)
S(2)-C(23)	1.769(6)
P(2)-C(30)	1.809(5)
P(2)-C(36)	1.817(6)
P(2)-C(22)	1.831(5)
P(1)-C(20)	1.813(5)
P(1)-C(8)	1.815(6)
P(1)-C(14)	1.818(5)
F(1)-C(5)	1.329(8)
F(4)-C(27)	1.351(9)
F(5)-C(27)	1.318(9)
F(2)-C(5)	1.319(8)
F(3)-C(5)	1.337(8)
F(6)-C(27)	1.325(9)
C(20)-C(21)	1.540(8)
C(20)-H(20A)	0.9900
C(20)-H(20B)	0.9900
C(37)-C(38)	1.382(8)
C(37)-C(36)	1.396(8)
C(37)-H(37)	0.9500
C(30)-C(31)	1.386(8)
C(30)-C(35)	1.400(8)
C(36)-C(41)	1.387(8)
C(23)-C(29)	1.384(8)
C(23)-C(24)	1.389(8)
C(31)-C(32)	1.390(8)
C(31)-H(31)	0.9500
C(21)-C(22)	1.541(7)
C(21)-H(21A)	0.9900
C(21)-H(21B)	0.9900
C(22)-H(22A)	0.9900
C(22)-H(22B)	0.9900
C(2)-C(1)	1.390(8)
C(2)-C(3)	1.392(9)

C(2)-H(2)	0.9500
C(24)-C(25)	1.382(9)
C(24)-H(24)	0.9500
C(9)-C(10)	1.381(9)
C(9)-C(8)	1.391(8)
C(9)-H(9)	0.9500
C(8)-C(13)	1.379(8)
C(41)-C(40)	1.396(9)
C(41)-H(41)	0.9500
C(14)-C(19)	1.393(8)
C(14)-C(15)	1.395(8)
C(15)-C(16)	1.396(8)
C(15)-H(15)	0.9500
C(1)-C(7)	1.380(8)
C(35)-C(34)	1.381(8)
C(35)-H(35)	0.9500
C(29)-C(28)	1.397(8)
C(29)-H(29)	0.9500
C(39)-C(40)	1.370(9)
C(39)-C(38)	1.386(9)
C(39)-H(39)	0.9500
C(17)-C(16)	1.363(9)
C(17)-C(18)	1.387(9)
C(17)-H(17)	0.9500
C(19)-C(18)	1.391(8)
C(19)-H(19)	0.9500
C(32)-C(33)	1.376(9)
C(32)-H(32)	0.9500
C(7)-C(6)	1.384(9)
C(7)-H(7)	0.9500
C(16)-H(16)	0.9500
C(13)-C(12)	1.401(9)
C(13)-H(13)	0.9500
C(34)-C(33)	1.388(9)
C(34)-H(34)	0.9500
C(38)-H(38)	0.9500
C(25)-C(26)	1.374(9)
C(25)-H(25)	0.9500
C(4)-C(6)	1.383(9)

C(4)-C(3)	1.391(9)
C(4)-C(5)	1.480(10)
C(26)-C(28)	1.385(9)
C(26)-C(27)	1.494(10)
C(6)-H(6)	0.9500
C(3)-H(3)	0.9500
C(40)-H(40)	0.9500
C(33)-H(33)	0.9500
C(28)-H(28)	0.9500
C(10)-C(11)	1.379(10)
C(10)-H(10)	0.9500
C(18)-H(18)	0.9500
C(11)-C(12)	1.385(10)
C(11)-H(11)	0.9500
C(12)-H(12)	0.9500
Cl(1)-C(42)	1.766(8)
Cl(2)-C(42)	1.734(9)
Cl(3)-C(42)	1.734(9)
C(42)-H(42)	1.0000
Cl(4)-C(43)	1.761(8)
Cl(5)-C(43)	1.749(8)
Cl(6)-C(43)	1.736(8)
C(43)-H(43)	1.0000
P(1)-Au(1)-S(1)	169.92(5)
P(1)-Au(1)-Au(2)#1	103.39(4)
S(1)-Au(1)-Au(2)#1	85.65(4)
P(2)-Au(2)-S(2)	165.83(5)
P(2)-Au(2)-Au(1)#2	94.37(3)
S(2)-Au(2)-Au(1)#2	96.79(4)
C(1)-S(1)-Au(1)	106.10(19)
C(23)-S(2)-Au(2)	112.47(18)
C(30)-P(2)-C(36)	105.8(3)
C(30)-P(2)-C(22)	105.7(2)
C(36)-P(2)-C(22)	102.4(2)
C(30)-P(2)-Au(2)	111.22(18)
C(36)-P(2)-Au(2)	112.36(18)
C(22)-P(2)-Au(2)	118.27(19)
C(20)-P(1)-C(8)	104.5(3)
C(20)-P(1)-C(14)	104.6(2)
C(8)-P(1)-C(14)	106.2(3)
C(20)-P(1)-Au(1)	118.02(18)

C(8)-P(1)-Au(1)	108.26(18)
C(14)-P(1)-Au(1)	114.19(18)
C(21)-C(20)-P(1)	112.7(4)
C(21)-C(20)-H(20A)	109.1
P(1)-C(20)-H(20A)	109.1
C(21)-C(20)-H(20B)	109.1
P(1)-C(20)-H(20B)	109.1
H(20A)-C(20)-H(20B)	107.8
C(38)-C(37)-C(36)	120.3(6)
C(38)-C(37)-H(37)	119.8
C(36)-C(37)-H(37)	119.8
C(31)-C(30)-C(35)	118.4(5)
C(31)-C(30)-P(2)	122.1(4)
C(35)-C(30)-P(2)	119.2(4)
C(41)-C(36)-C(37)	119.4(5)
C(41)-C(36)-P(2)	120.5(4)
C(37)-C(36)-P(2)	119.9(4)
C(29)-C(23)-C(24)	118.2(6)
C(29)-C(23)-S(2)	118.9(4)
C(24)-C(23)-S(2)	122.4(4)
C(30)-C(31)-C(32)	120.5(6)
C(30)-C(31)-H(31)	119.8
C(32)-C(31)-H(31)	119.8
C(20)-C(21)-C(22)	111.5(4)
C(20)-C(21)-H(21A)	109.3
C(22)-C(21)-H(21A)	109.3
C(20)-C(21)-H(21B)	109.3
C(22)-C(21)-H(21B)	109.3
H(21A)-C(21)-H(21B)	108.0
C(21)-C(22)-P(2)	112.2(4)
C(21)-C(22)-H(22A)	109.2
P(2)-C(22)-H(22A)	109.2
C(21)-C(22)-H(22B)	109.2
P(2)-C(22)-H(22B)	109.2
H(22A)-C(22)-H(22B)	107.9
C(1)-C(2)-C(3)	120.9(6)
C(1)-C(2)-H(2)	119.5
C(3)-C(2)-H(2)	119.5
C(25)-C(24)-C(23)	120.7(6)
C(25)-C(24)-H(24)	119.6
C(23)-C(24)-H(24)	119.6
C(10)-C(9)-C(8)	120.5(6)

C(10)-C(9)-H(9)	119.8
C(8)-C(9)-H(9)	119.8
C(13)-C(8)-C(9)	119.3(6)
C(13)-C(8)-P(1)	122.3(5)
C(9)-C(8)-P(1)	118.2(4)
C(36)-C(41)-C(40)	119.8(5)
C(36)-C(41)-H(41)	120.1
C(40)-C(41)-H(41)	120.1
C(19)-C(14)-C(15)	120.2(5)
C(19)-C(14)-P(1)	120.0(4)
C(15)-C(14)-P(1)	119.6(4)
C(14)-C(15)-C(16)	119.0(6)
C(14)-C(15)-H(15)	120.5
C(16)-C(15)-H(15)	120.5
C(7)-C(1)-C(2)	118.7(6)
C(7)-C(1)-S(1)	117.2(4)
C(2)-C(1)-S(1)	124.0(5)
C(34)-C(35)-C(30)	120.7(5)
C(34)-C(35)-H(35)	119.6
C(30)-C(35)-H(35)	119.6
C(23)-C(29)-C(28)	121.6(6)
C(23)-C(29)-H(29)	119.2
C(28)-C(29)-H(29)	119.2
C(40)-C(39)-C(38)	120.3(6)
C(40)-C(39)-H(39)	119.9
C(38)-C(39)-H(39)	119.9
C(16)-C(17)-C(18)	120.9(6)
C(16)-C(17)-H(17)	119.6
C(18)-C(17)-H(17)	119.6
C(18)-C(19)-C(14)	119.7(5)
C(18)-C(19)-H(19)	120.2
C(14)-C(19)-H(19)	120.2
C(33)-C(32)-C(31)	120.8(6)
C(33)-C(32)-H(32)	119.6
C(31)-C(32)-H(32)	119.6
C(1)-C(7)-C(6)	120.7(6)
C(1)-C(7)-H(7)	119.6
C(6)-C(7)-H(7)	119.6
C(17)-C(16)-C(15)	120.5(6)
C(17)-C(16)-H(16)	119.7
C(15)-C(16)-H(16)	119.7
C(8)-C(13)-C(12)	120.3(6)

C(8)-C(13)-H(13)	119.9
C(12)-C(13)-H(13)	119.9
C(35)-C(34)-C(33)	120.4(6)
C(35)-C(34)-H(34)	119.8
C(33)-C(34)-H(34)	119.8
C(37)-C(38)-C(39)	119.9(6)
C(37)-C(38)-H(38)	120.1
C(39)-C(38)-H(38)	120.1
C(26)-C(25)-C(24)	120.4(6)
C(26)-C(25)-H(25)	119.8
C(24)-C(25)-H(25)	119.8
C(6)-C(4)-C(3)	119.2(6)
C(6)-C(4)-C(5)	121.7(6)
C(3)-C(4)-C(5)	119.0(6)
C(25)-C(26)-C(28)	120.4(6)
C(25)-C(26)-C(27)	118.8(6)
C(28)-C(26)-C(27)	120.7(6)
C(7)-C(6)-C(4)	120.7(6)
C(7)-C(6)-H(6)	119.7
C(4)-C(6)-H(6)	119.7
C(4)-C(3)-C(2)	119.7(6)
C(4)-C(3)-H(3)	120.2
C(2)-C(3)-H(3)	120.2
C(39)-C(40)-C(41)	120.3(6)
C(39)-C(40)-H(40)	119.8
C(41)-C(40)-H(40)	119.8
C(32)-C(33)-C(34)	119.2(5)
C(32)-C(33)-H(33)	120.4
C(34)-C(33)-H(33)	120.4
C(26)-C(28)-C(29)	118.6(6)
C(26)-C(28)-H(28)	120.7
C(29)-C(28)-H(28)	120.7
C(11)-C(10)-C(9)	120.4(6)
C(11)-C(10)-H(10)	119.8
C(9)-C(10)-H(10)	119.8
C(17)-C(18)-C(19)	119.6(6)
C(17)-C(18)-H(18)	120.2
C(19)-C(18)-H(18)	120.2
C(10)-C(11)-C(12)	119.8(6)
C(10)-C(11)-H(11)	120.1
C(12)-C(11)-H(11)	120.1
F(5)-C(27)-F(6)	106.5(7)

F(5)-C(27)-F(4)	104.9(7)
F(6)-C(27)-F(4)	105.7(7)
F(5)-C(27)-C(26)	113.3(6)
F(6)-C(27)-C(26)	113.6(7)
F(4)-C(27)-C(26)	112.1(7)
F(2)-C(5)-F(1)	106.4(6)
F(2)-C(5)-F(3)	105.3(7)
F(1)-C(5)-F(3)	106.3(6)
F(2)-C(5)-C(4)	113.0(6)
F(1)-C(5)-C(4)	112.6(6)
F(3)-C(5)-C(4)	112.5(6)
C(11)-C(12)-C(13)	119.7(6)
C(11)-C(12)-H(12)	120.1

C(13)-C(12)-H(12)	120.1
Cl(2)-C(42)-Cl(3)	111.0(5)
Cl(2)-C(42)-Cl(1)	109.3(5)
Cl(3)-C(42)-Cl(1)	110.2(5)
Cl(2)-C(42)-H(42)	108.8
Cl(3)-C(42)-H(42)	108.8
Cl(1)-C(42)-H(42)	108.8
Cl(6)-C(43)-Cl(5)	106.8(5)
Cl(6)-C(43)-Cl(4)	113.0(5)
Cl(5)-C(43)-Cl(4)	111.1(4)
Cl(6)-C(43)-H(43)	108.6
Cl(5)-C(43)-H(43)	108.6
Cl(4)-C(43)-H(43)	108.6

Table S13. Crystal data and structure refinement for **9**.

Identification code	**9**	
Empirical formula	$C_{39} H_{33} Au_2 F_2 P_2 S_2$	
Formula weight	1059.65	
Temperature	130(2) K	
Wavelength	0.71073 Å	
Crystal system	Triclinic	
Space group	P -1	
Unit cell dimensions	a = 8.7519(5) Å	α= 93.371(5)°.
	b = 11.6131(7) Å	β= 92.386(4)°.
	c = 17.9964(10) Å	γ = 108.178(5)°.
Volume	1731.33(18) $Å^3$	
Z	2	
Density (calculated)	2.033 Mg/m^3	
Absorption coefficient	8.717 mm^{-1}	
F(000)	1010	
Crystal size	0.460 x 0.370 x 0.350 mm^3	
Theta range for data collection	3.439 to 30.206°.	
Index ranges	-11<=h<=11, -16<=k<=15, -21<=l<=22	
Reflections collected	17088	
Independent reflections	8092 [R(int) = 0.1130]	
Completeness to theta = 25.242°	99.7 %	
Refinement method	Full-matrix least-squares on F^2	
Data / restraints / parameters	8092 / 0 / 433	
Goodness-of-fit on F^2	0.953	
Final R indices [I>2sigma(I)]	R1 = 0.0539, wR2 = 0.1230	
R indices (all data)	R1 = 0.0886, wR2 = 0.1609	
Largest diff. peak and hole	4.006 and -4.769 e.$Å^{-3}$	

Table S14. Atomic coordinates (x 10^4) and equivalent isotropic displacement parameters ($Å^2$x 10^3) for **9**. U(eq) is defined as one third of the trace of the orthogonalized U^{ij} tensor.

	x	y	z	U(eq)
Au(1)	1185(1)	4434(1)	1846(1)	20(1)
Au(2)	-5484(1)	6243(1)	2501(1)	21(1)
C(1)	3659(12)	2784(9)	1664(5)	20(2)
C(2)	3616(13)	2796(10)	2444(6)	26(2)
C(3)	4572(14)	2286(11)	2844(7)	33(3)
C(4)	5625(15)	1767(10)	2512(7)	34(3)
C(5)	5675(15)	1761(11)	1751(7)	37(3)
C(6)	4707(14)	2245(10)	1330(6)	29(2)
C(7)	-1002(12)	4531(10)	3345(5)	21(2)
C(8)	-2327(13)	4631(10)	3710(6)	27(2)
C(9)	-2693(14)	4127(12)	4375(6)	35(3)
C(10)	-1803(15)	3445(12)	4698(7)	38(3)
C(11)	-487(15)	3328(11)	4310(7)	39(3)
C(12)	-89(14)	3858(11)	3659(6)	32(3)
C(13)	469(12)	6886(10)	2784(6)	23(2)
C(14)	52(13)	7422(10)	3424(6)	26(2)
C(15)	639(14)	8668(11)	3598(7)	34(3)
C(16)	1636(14)	9400(11)	3118(6)	30(3)
C(17)	2058(14)	8873(12)	2479(7)	35(3)
C(18)	1467(12)	7651(11)	2310(6)	26(2)
C(19)	-2286(12)	5192(10)	1988(6)	24(2)
C(20)	-2030(12)	5889(11)	1279(5)	25(2)
C(21)	-3620(12)	5962(10)	892(5)	22(2)
C(22)	-4821(13)	7267(11)	4110(6)	30(3)
C(23)	-3548(15)	7434(11)	4607(6)	32(3)
C(24)	-2697(16)	8563(13)	4907(7)	42(3)
C(25)	-3003(16)	9605(13)	4700(7)	43(3)
C(26)	-4308(17)	9437(13)	4208(6)	43(3)
C(27)	-5232(14)	8271(12)	3926(6)	35(3)
C(28)	-2868(12)	8385(10)	1592(6)	25(2)
C(29)	-1843(13)	8842(10)	1036(6)	28(2)
C(30)	-652(15)	9934(12)	1158(7)	39(3)
C(31)	-434(15)	10592(12)	1848(8)	45(3)
C(32)	-1433(14)	10135(11)	2394(7)	38(3)
C(33)	-2645(12)	9012(10)	2277(6)	25(2)
C(34)	-5910(12)	7321(10)	792(5)	20(2)
C(35)	-7123(13)	6406(9)	394(5)	22(2)

C(36)	-8302(13)	6689(11)	-31(6)	27(2)
C(37)	-8284(14)	7879(11)	-75(6)	33(3)
C(38)	-7061(14)	8810(12)	316(7)	38(3)
C(39)	-5884(14)	8522(10)	742(6)	27(2)
F(2)	-1374(11)	8740(9)	5375(6)	80(3)
P(1)	-403(3)	5284(3)	2504(1)	20(1)
P(2)	-4478(3)	6965(3)	1423(1)	20(1)
S(1)	2451(3)	3379(3)	1090(1)	24(1)
S(2)	-5951(3)	5783(3)	3719(2)	30(1)
F(1)	4408(11)	2223(8)	3575(4)	36(2)
F(1A)	6540(30)	1230(30)	1448(18)	47(8)

Table S15. Bond lengths [Å] and angles [°] for **9**.

Au(1)-P(1)	2.272(2)
Au(1)-S(1)	2.314(2)
Au(1)-Au(2)#1	3.1475(6)
Au(2)-P(2)	2.263(3)
Au(2)-S(2)	2.309(3)
Au(2)-Au(1)#2	3.1475(6)
C(1)-C(6)	1.398(14)
C(1)-C(2)	1.404(14)
C(1)-S(1)	1.766(10)
C(2)-C(3)	1.371(16)
C(2)-H(2)	0.9500
C(3)-F(1)	1.332(14)
C(3)-C(4)	1.386(17)
C(4)-C(5)	1.372(17)
C(4)-H(4)	0.9500
C(5)-F(1A)	1.24(3)
C(5)-C(6)	1.380(16)
C(6)-H(6)	0.9500
C(7)-C(8)	1.388(14)
C(7)-C(12)	1.404(15)
C(7)-P(1)	1.808(10)
C(8)-C(9)	1.369(16)
C(8)-H(8)	0.9500
C(9)-C(10)	1.405(17)
C(9)-H(9)	0.9500
C(10)-C(11)	1.410(16)
C(10)-H(10)	0.9500
C(11)-C(12)	1.363(17)
C(11)-H(11)	0.9500
C(12)-H(12)	0.9500
C(13)-C(14)	1.392(13)
C(13)-C(18)	1.396(15)
C(13)-P(1)	1.808(11)
C(14)-C(15)	1.387(16)
C(14)-H(14)	0.9500
C(15)-C(16)	1.385(17)
C(15)-H(15)	0.9500
C(16)-C(17)	1.387(16)
C(16)-H(16)	0.9500
C(17)-C(18)	1.361(16)
C(17)-H(17)	0.9500
C(18)-H(18)	0.9500
C(19)-C(20)	1.538(15)
C(19)-P(1)	1.827(10)
C(19)-H(19A)	0.9900
C(19)-H(19B)	0.9900
C(20)-C(21)	1.559(14)
C(20)-H(20A)	0.9900
C(20)-H(20B)	0.9900
C(21)-P(2)	1.819(10)
C(21)-H(21A)	0.9900
C(21)-H(21B)	0.9900
C(22)-C(23)	1.356(15)
C(22)-C(27)	1.377(17)
C(22)-S(2)	1.783(12)
C(23)-C(24)	1.356(18)
C(23)-H(23)	0.9500
C(24)-F(2)	1.358(14)
C(24)-C(25)	1.386(18)
C(25)-C(26)	1.372(17)
C(25)-H(25)	0.9500
C(26)-C(27)	1.396(18)
C(26)-H(26)	0.9500
C(27)-H(27)	0.9500
C(28)-C(33)	1.369(15)
C(28)-C(29)	1.388(15)
C(28)-P(2)	1.803(11)
C(29)-C(30)	1.366(17)
C(29)-H(29)	0.9500
C(30)-C(31)	1.393(19)
C(30)-H(30)	0.9500
C(31)-C(32)	1.362(19)
C(31)-H(31)	0.9500
C(32)-C(33)	1.399(16)
C(32)-H(32)	0.9500
C(33)-H(33)	0.9500
C(34)-C(35)	1.383(14)
C(34)-C(39)	1.396(15)

C(34)-P(2)	1.813(10)
C(35)-C(36)	1.391(14)
C(35)-H(35)	0.9500
C(36)-C(37)	1.384(16)
C(36)-H(36)	0.9500
C(37)-C(38)	1.392(16)
C(37)-H(37)	0.9500
C(38)-C(39)	1.394(16)
C(38)-H(38)	0.9500
C(39)-H(39)	0.9500
P(1)-Au(1)-S(1)	171.54(10)
P(1)-Au(1)-Au(2)#1	96.89(7)
S(1)-Au(1)-Au(2)#1	91.55(7)
P(2)-Au(2)-S(2)	167.01(10)
P(2)-Au(2)-Au(1)#2	98.66(7)
S(2)-Au(2)-Au(1)#2	94.31(7)
C(6)-C(1)-C(2)	117.4(9)
C(6)-C(1)-S(1)	118.7(8)
C(2)-C(1)-S(1)	123.9(8)
C(3)-C(2)-C(1)	119.9(10)
C(3)-C(2)-H(2)	120.0
C(1)-C(2)-H(2)	120.0
F(1)-C(3)-C(2)	118.5(11)
F(1)-C(3)-C(4)	118.8(11)
C(2)-C(3)-C(4)	122.6(11)
C(5)-C(4)-C(3)	117.5(11)
C(5)-C(4)-H(4)	121.2
C(3)-C(4)-H(4)	121.2
F(1A)-C(5)-C(4)	117.5(18)
F(1A)-C(5)-C(6)	120.9(19)
C(4)-C(5)-C(6)	121.4(11)
C(5)-C(6)-C(1)	121.1(10)
C(5)-C(6)-H(6)	119.4
C(1)-C(6)-H(6)	119.4
C(8)-C(7)-C(12)	118.4(10)
C(8)-C(7)-P(1)	121.6(8)
C(12)-C(7)-P(1)	120.0(8)
C(9)-C(8)-C(7)	120.7(10)
C(9)-C(8)-H(8)	119.7
C(7)-C(8)-H(8)	119.7
C(8)-C(9)-C(10)	122.0(11)

C(8)-C(9)-H(9)	119.0
C(10)-C(9)-H(9)	119.0
C(9)-C(10)-C(11)	116.4(11)
C(9)-C(10)-H(10)	121.8
C(11)-C(10)-H(10)	121.8
C(12)-C(11)-C(10)	121.8(11)
C(12)-C(11)-H(11)	119.1
C(10)-C(11)-H(11)	119.1
C(11)-C(12)-C(7)	120.7(11)
C(11)-C(12)-H(12)	119.7
C(7)-C(12)-H(12)	119.7
C(14)-C(13)-C(18)	117.6(10)
C(14)-C(13)-P(1)	122.5(8)
C(18)-C(13)-P(1)	119.6(8)
C(15)-C(14)-C(13)	121.4(11)
C(15)-C(14)-H(14)	119.3
C(13)-C(14)-H(14)	119.3
C(16)-C(15)-C(14)	119.5(11)
C(16)-C(15)-H(15)	120.3
C(14)-C(15)-H(15)	120.3
C(15)-C(16)-C(17)	119.4(11)
C(15)-C(16)-H(16)	120.3
C(17)-C(16)-H(16)	120.3
C(18)-C(17)-C(16)	120.7(12)
C(18)-C(17)-H(17)	119.6
C(16)-C(17)-H(17)	119.6
C(17)-C(18)-C(13)	121.3(10)
C(17)-C(18)-H(18)	119.4
C(13)-C(18)-H(18)	119.4
C(20)-C(19)-P(1)	113.2(7)
C(20)-C(19)-H(19A)	108.9
P(1)-C(19)-H(19A)	108.9
C(20)-C(19)-H(19B)	108.9
P(1)-C(19)-H(19B)	108.9
H(19A)-C(19)-H(19B)	107.7
C(19)-C(20)-C(21)	113.7(8)
C(19)-C(20)-H(20A)	108.8
C(21)-C(20)-H(20A)	108.8
C(19)-C(20)-H(20B)	108.8
C(21)-C(20)-H(20B)	108.8
H(20A)-C(20)-H(20B)	107.7
C(20)-C(21)-P(2)	112.4(7)

C(20)-C(21)-H(21A)	109.1
P(2)-C(21)-H(21A)	109.1
C(20)-C(21)-H(21B)	109.1
P(2)-C(21)-H(21B)	109.1
H(21A)-C(21)-H(21B)	107.9
C(23)-C(22)-C(27)	118.2(11)
C(23)-C(22)-S(2)	120.8(10)
C(27)-C(22)-S(2)	121.0(9)
C(24)-C(23)-C(22)	120.7(12)
C(24)-C(23)-H(23)	119.7
C(22)-C(23)-H(23)	119.7
C(23)-C(24)-F(2)	120.8(12)
C(23)-C(24)-C(25)	122.9(11)
F(2)-C(24)-C(25)	115.9(12)
C(26)-C(25)-C(24)	116.4(12)
C(26)-C(25)-H(25)	121.8
C(24)-C(25)-H(25)	121.8
C(25)-C(26)-C(27)	120.6(12)
C(25)-C(26)-H(26)	119.7
C(27)-C(26)-H(26)	119.7
C(22)-C(27)-C(26)	121.0(11)
C(22)-C(27)-H(27)	119.5
C(26)-C(27)-H(27)	119.5
C(33)-C(28)-C(29)	119.7(11)
C(33)-C(28)-P(2)	119.7(9)
C(29)-C(28)-P(2)	120.6(8)
C(30)-C(29)-C(28)	120.5(11)
C(30)-C(29)-H(29)	119.7
C(28)-C(29)-H(29)	119.7
C(29)-C(30)-C(31)	120.2(12)
C(29)-C(30)-H(30)	119.9
C(31)-C(30)-H(30)	119.9
C(32)-C(31)-C(30)	119.1(12)
C(32)-C(31)-H(31)	120.5
C(30)-C(31)-H(31)	120.5
C(31)-C(32)-C(33)	121.1(12)
C(31)-C(32)-H(32)	119.5
C(33)-C(32)-H(32)	119.5
C(28)-C(33)-C(32)	119.3(11)
C(28)-C(33)-H(33)	120.3
C(32)-C(33)-H(33)	120.3
C(35)-C(34)-C(39)	118.5(9)
C(35)-C(34)-P(2)	120.8(8)
C(39)-C(34)-P(2)	120.4(8)
C(34)-C(35)-C(36)	120.1(10)
C(34)-C(35)-H(35)	120.0
C(36)-C(35)-H(35)	120.0
C(37)-C(36)-C(35)	121.4(11)
C(37)-C(36)-H(36)	119.3
C(35)-C(36)-H(36)	119.3
C(36)-C(37)-C(38)	119.2(10)
C(36)-C(37)-H(37)	120.4
C(38)-C(37)-H(37)	120.4
C(37)-C(38)-C(39)	119.2(12)
C(37)-C(38)-H(38)	120.4
C(39)-C(38)-H(38)	120.4
C(38)-C(39)-C(34)	121.6(10)
C(38)-C(39)-H(39)	119.2
C(34)-C(39)-H(39)	119.2
C(7)-P(1)-C(13)	106.4(5)
C(7)-P(1)-C(19)	105.3(5)
C(13)-P(1)-C(19)	103.5(5)
C(7)-P(1)-Au(1)	111.8(3)
C(13)-P(1)-Au(1)	115.7(3)
C(19)-P(1)-Au(1)	113.2(3)
C(28)-P(2)-C(34)	103.8(5)
C(28)-P(2)-C(21)	105.3(5)
C(34)-P(2)-C(21)	106.9(5)
C(28)-P(2)-Au(2)	111.4(4)
C(34)-P(2)-Au(2)	114.6(3)
C(21)-P(2)-Au(2)	114.0(4)
C(1)-S(1)-Au(1)	108.4(3)
C(22)-S(2)-Au(2)	94.5(4)

Table S16. Crystal data and structure refinement for **10**.

Identification code	**10**	
Empirical formula	$C_{39} H_{34} Au_2 F_2 P_2 S_2$	
Formula weight	1060.65	
Temperature	130(2) K	
Wavelength	0.71073 Å	
Crystal system	Triclinic	
Space group	P -1	
Unit cell dimensions	a = 8.6845(3) Å	α= 88.207(4)°.
	b = 11.7604(6) Å	β= 87.070(3)°.
	c = 18.2867(9) Å	γ = 71.711(4)°.
Volume	1770.79(15) $Å^3$	
Z	2	
Density (calculated)	1.989 Mg/m^3	
Absorption coefficient	8.522 mm^{-1}	
F(000)	1012	
Crystal size	0.230 x 0.120 x 0.080 mm^3	
Theta range for data collection	3.402 to 30.136°.	
Index ranges	-12<=h<=11, -16<=k<=16, -25<=l<=25	
Reflections collected	25527	
Independent reflections	9104 [R(int) = 0.0369]	
Completeness to theta = 25.242°	99.8 %	
Refinement method	Full-matrix least-squares on F^2	
Data / restraints / parameters	9104 / 0 / 424	
Goodness-of-fit on F^2	1.053	
Final R indices [I>2sigma(I)]	R1 = 0.0270, wR2 = 0.0494	
R indices (all data)	R1 = 0.0371, wR2 = 0.0538	
Largest diff. peak and hole	1.146 and -1.017 e.$Å^{-3}$	

Table S17. Atomic coordinates (x 10^4) and equivalent isotropic displacement parameters ($Å^2$x 10^3) for **10**. U(eq) is defined as one third of the trace of the orthogonalized U^{ij} tensor.

	x	y	z	U(eq)
C(1)	9906(4)	1538(4)	4095(2)	23(1)
C(2)	10059(5)	2678(4)	3972(2)	31(1)
C(3)	9088(6)	3650(4)	4366(2)	41(1)
C(4)	7994(6)	3461(4)	4884(2)	41(1)
C(5)	7811(5)	2349(4)	5022(2)	36(1)
C(6)	8760(5)	1403(4)	4624(2)	27(1)
C(7)	10892(4)	2246(3)	741(2)	14(1)
C(8)	12081(4)	1382(3)	337(2)	20(1)
C(9)	13248(4)	1693(3)	-91(2)	24(1)
C(10)	13251(5)	2865(4)	-140(2)	28(1)
C(11)	12076(5)	3737(4)	249(2)	29(1)
C(12)	10912(4)	3425(3)	689(2)	25(1)
C(13)	7829(4)	3214(3)	1527(2)	18(1)
C(14)	7600(4)	3764(3)	2199(2)	23(1)
C(15)	6364(5)	4827(4)	2312(2)	30(1)
C(16)	5347(5)	5338(4)	1756(3)	34(1)
C(17)	5557(5)	4789(4)	1089(2)	30(1)
C(18)	6791(4)	3731(3)	971(2)	24(1)
C(19)	8585(4)	885(3)	879(2)	17(1)
C(20)	7033(4)	753(3)	1266(2)	17(1)
C(21)	7326(4)	-65(3)	1942(2)	18(1)
C(22)	1370(4)	-2410(3)	1673(2)	18(1)
C(23)	1481(4)	-2449(3)	2432(2)	22(1)
C(24)	697(4)	-3093(4)	2867(2)	26(1)
C(25)	-220(5)	-3683(3)	2543(2)	28(1)
C(26)	-408(5)	-3629(4)	1804(2)	28(1)
C(27)	388(4)	-2998(3)	1369(2)	25(1)
C(28)	4617(4)	1445(3)	2778(2)	19(1)
C(29)	3627(4)	2295(3)	2308(2)	22(1)
C(30)	3029(5)	3491(4)	2504(2)	27(1)
C(31)	3377(5)	3849(4)	3172(2)	32(1)
C(32)	4336(5)	3013(4)	3640(2)	30(1)
C(33)	4940(5)	1819(4)	3451(2)	27(1)
C(34)	6203(4)	-1058(3)	3237(2)	21(1)
C(35)	7457(4)	-955(4)	3647(2)	25(1)
C(36)	7976(5)	-1719(4)	4243(2)	30(1)
C(37)	7282(5)	-2603(4)	4424(2)	33(1)

C(38)	6072(5)	-2739(4)	4005(2)	32(1)
C(39)	5522(4)	-1973(3)	3422(2)	25(1)
Au(1)	10549(1)	980(1)	2441(1)	16(1)
Au(2)	3815(1)	-767(1)	1796(1)	16(1)
F(1)	7049(4)	4424(3)	5278(2)	68(1)
F(2)	-938(3)	-4352(2)	2981(2)	45(1)
P(1)	9482(1)	1826(1)	1379(1)	14(1)
P(2)	5474(1)	-81(1)	2458(1)	16(1)
S(1)	11178(1)	261(1)	3612(1)	25(1)
S(2)	2446(1)	-1680(1)	1073(1)	21(1)

Table S18 Bond lengths [Å] and angles [°] for **10**.

C(1)-C(6)	1.392(5)
C(1)-C(2)	1.399(6)
C(1)-S(1)	1.789(4)
C(2)-C(3)	1.386(6)
C(2)-H(2)	0.9500
C(3)-C(4)	1.370(7)
C(3)-H(3)	0.9500
C(4)-F(1)	1.372(5)
C(4)-C(5)	1.380(7)
C(5)-C(6)	1.364(6)
C(5)-H(5)	0.9500
C(6)-H(6)	0.9500
C(7)-C(12)	1.393(5)
C(7)-C(8)	1.398(5)
C(7)-P(1)	1.817(4)
C(8)-C(9)	1.381(5)
C(8)-H(8)	0.9500
C(9)-C(10)	1.379(5)
C(9)-H(9)	0.9500
C(10)-C(11)	1.383(5)
C(10)-H(10)	0.9500
C(11)-C(12)	1.391(5)
C(11)-H(11)	0.9500
C(12)-H(12)	0.9500
C(13)-C(14)	1.382(5)
C(13)-C(18)	1.390(5)
C(13)-P(1)	1.821(4)
C(14)-C(15)	1.382(5)
C(14)-H(14)	0.9500
C(15)-C(16)	1.377(6)
C(15)-H(15)	0.9500
C(16)-C(17)	1.375(6)
C(16)-H(16)	0.9500
C(17)-C(18)	1.378(5)
C(17)-H(17)	0.9500
C(18)-H(18)	0.9500
C(19)-C(20)	1.539(5)
C(19)-P(1)	1.830(3)
C(19)-H(19A)	0.9900

C(19)-H(19B)	0.9900
C(20)-C(21)	1.524(5)
C(20)-H(20A)	0.9900
C(20)-H(20B)	0.9900
C(21)-P(2)	1.828(3)
C(21)-H(21A)	0.9900
C(21)-H(21B)	0.9900
C(22)-C(23)	1.394(5)
C(22)-C(27)	1.400(5)
C(22)-S(2)	1.776(4)
C(23)-C(24)	1.379(5)
C(23)-H(23)	0.9500
C(24)-C(25)	1.373(6)
C(24)-H(24)	0.9500
C(25)-F(2)	1.366(4)
C(25)-C(26)	1.367(6)
C(26)-C(27)	1.375(6)
C(26)-H(26)	0.9500
C(27)-H(27)	0.9500
C(28)-C(33)	1.391(5)
C(28)-C(29)	1.400(5)
C(28)-P(2)	1.816(4)
C(29)-C(30)	1.390(5)
C(29)-H(29)	0.9500
C(30)-C(31)	1.382(6)
C(30)-H(30)	0.9500
C(31)-C(32)	1.378(6)
C(31)-H(31)	0.9500
C(32)-C(33)	1.384(5)
C(32)-H(32)	0.9500
C(33)-H(33)	0.9500
C(34)-C(35)	1.391(5)
C(34)-C(39)	1.407(5)
C(34)-P(2)	1.812(4)
C(35)-C(36)	1.390(5)
C(35)-H(35)	0.9500
C(36)-C(37)	1.380(6)
C(36)-H(36)	0.9500
C(37)-C(38)	1.384(6)

C(37)-H(37)	0.9500
C(38)-C(39)	1.377(5)
C(38)-H(38)	0.9500
C(39)-H(39)	0.9500
Au(1)-P(1)	2.2589(9)
Au(1)-S(1)	2.3049(9)
Au(1)-Au(2)#1	3.1325(2)
Au(2)-P(2)	2.2712(9)
Au(2)-S(2)	2.3162(9)
C(6)-C(1)-C(2)	118.5(4)
C(6)-C(1)-S(1)	120.0(3)
C(2)-C(1)-S(1)	121.5(3)
C(3)-C(2)-C(1)	120.6(4)
C(3)-C(2)-H(2)	119.7
C(1)-C(2)-H(2)	119.7
C(4)-C(3)-C(2)	118.4(4)
C(4)-C(3)-H(3)	120.8
C(2)-C(3)-H(3)	120.8
C(3)-C(4)-F(1)	118.1(5)
C(3)-C(4)-C(5)	122.6(4)
F(1)-C(4)-C(5)	119.3(4)
C(6)-C(5)-C(4)	118.4(4)
C(6)-C(5)-H(5)	120.8
C(4)-C(5)-H(5)	120.8
C(5)-C(6)-C(1)	121.5(4)
C(5)-C(6)-H(6)	119.2
C(1)-C(6)-H(6)	119.2
C(12)-C(7)-C(8)	117.9(3)
C(12)-C(7)-P(1)	120.8(3)
C(8)-C(7)-P(1)	121.1(3)
C(9)-C(8)-C(7)	120.7(3)
C(9)-C(8)-H(8)	119.7
C(7)-C(8)-H(8)	119.7
C(10)-C(9)-C(8)	120.8(4)
C(10)-C(9)-H(9)	119.6
C(8)-C(9)-H(9)	119.6
C(9)-C(10)-C(11)	119.5(4)
C(9)-C(10)-H(10)	120.3
C(11)-C(10)-H(10)	120.3
C(10)-C(11)-C(12)	119.9(4)
C(10)-C(11)-H(11)	120.0

C(12)-C(11)-H(11)	120.0
C(11)-C(12)-C(7)	121.2(3)
C(11)-C(12)-H(12)	119.4
C(7)-C(12)-H(12)	119.4
C(14)-C(13)-C(18)	119.4(3)
C(14)-C(13)-P(1)	119.9(3)
C(18)-C(13)-P(1)	120.7(3)
C(15)-C(14)-C(13)	120.3(4)
C(15)-C(14)-H(14)	119.8
C(13)-C(14)-H(14)	119.8
C(16)-C(15)-C(14)	120.0(4)
C(16)-C(15)-H(15)	120.0
C(14)-C(15)-H(15)	120.0
C(17)-C(16)-C(15)	120.0(4)
C(17)-C(16)-H(16)	120.0
C(15)-C(16)-H(16)	120.0
C(16)-C(17)-C(18)	120.4(4)
C(16)-C(17)-H(17)	119.8
C(18)-C(17)-H(17)	119.8
C(17)-C(18)-C(13)	119.9(4)
C(17)-C(18)-H(18)	120.0
C(13)-C(18)-H(18)	120.0
C(20)-C(19)-P(1)	112.9(2)
C(20)-C(19)-H(19A)	109.0
P(1)-C(19)-H(19A)	109.0
C(20)-C(19)-H(19B)	109.0
P(1)-C(19)-H(19B)	109.0
H(19A)-C(19)-H(19B)	107.8
C(21)-C(20)-C(19)	114.5(3)
C(21)-C(20)-H(20A)	108.6
C(19)-C(20)-H(20A)	108.6
C(21)-C(20)-H(20B)	108.6
C(19)-C(20)-H(20B)	108.6
H(20A)-C(20)-H(20B)	107.6
C(20)-C(21)-P(2)	114.2(2)
C(20)-C(21)-H(21A)	108.7
P(2)-C(21)-H(21A)	108.7
C(20)-C(21)-H(21B)	108.7
P(2)-C(21)-H(21B)	108.7
H(21A)-C(21)-H(21B)	107.6
C(23)-C(22)-C(27)	118.3(3)
C(23)-C(22)-S(2)	123.3(3)

C(27)-C(22)-S(2)	118.4(3)
C(24)-C(23)-C(22)	120.7(4)
C(24)-C(23)-H(23)	119.6
C(22)-C(23)-H(23)	119.6
C(25)-C(24)-C(23)	119.0(4)
C(25)-C(24)-H(24)	120.5
C(23)-C(24)-H(24)	120.5
F(2)-C(25)-C(26)	119.9(4)
F(2)-C(25)-C(24)	118.1(4)
C(26)-C(25)-C(24)	122.0(4)
C(25)-C(26)-C(27)	119.0(4)
C(25)-C(26)-H(26)	120.5
C(27)-C(26)-H(26)	120.5
C(26)-C(27)-C(22)	120.9(4)
C(26)-C(27)-H(27)	119.6
C(22)-C(27)-H(27)	119.6
C(33)-C(28)-C(29)	118.5(3)
C(33)-C(28)-P(2)	123.5(3)
C(29)-C(28)-P(2)	117.9(3)
C(30)-C(29)-C(28)	120.3(4)
C(30)-C(29)-H(29)	119.8
C(28)-C(29)-H(29)	119.8
C(31)-C(30)-C(29)	120.4(4)
C(31)-C(30)-H(30)	119.8
C(29)-C(30)-H(30)	119.8
C(32)-C(31)-C(30)	119.5(4)
C(32)-C(31)-H(31)	120.3
C(30)-C(31)-H(31)	120.3
C(31)-C(32)-C(33)	120.8(4)
C(31)-C(32)-H(32)	119.6
C(33)-C(32)-H(32)	119.6
C(32)-C(33)-C(28)	120.5(4)
C(32)-C(33)-H(33)	119.7
C(28)-C(33)-H(33)	119.7
C(35)-C(34)-C(39)	118.7(3)
C(35)-C(34)-P(2)	121.9(3)
C(39)-C(34)-P(2)	119.4(3)
C(34)-C(35)-C(36)	120.1(4)
C(34)-C(35)-H(35)	120.0
C(36)-C(35)-H(35)	120.0
C(37)-C(36)-C(35)	120.6(4)
C(37)-C(36)-H(36)	119.7

C(35)-C(36)-H(36)	119.7
C(36)-C(37)-C(38)	119.7(4)
C(36)-C(37)-H(37)	120.1
C(38)-C(37)-H(37)	120.1
C(39)-C(38)-C(37)	120.3(4)
C(39)-C(38)-H(38)	119.9
C(37)-C(38)-H(38)	119.9
C(38)-C(39)-C(34)	120.5(4)
C(38)-C(39)-H(39)	119.7
C(34)-C(39)-H(39)	119.7
P(1)-Au(1)-S(1)	169.88(3)
P(1)-Au(1)-Au(2)#1	98.41(2)
S(1)-Au(1)-Au(2)#1	91.36(2)
P(2)-Au(2)-S(2)	172.02(3)
P(2)-Au(2)-Au(1)#2	96.23(2)
S(2)-Au(2)-Au(1)#2	91.64(2)
C(7)-P(1)-C(13)	104.27(16)
C(7)-P(1)-C(19)	106.82(16)
C(13)-P(1)-C(19)	104.52(16)
C(7)-P(1)-Au(1)	114.96(11)
C(13)-P(1)-Au(1)	112.05(13)
C(19)-P(1)-Au(1)	113.30(12)
C(34)-P(2)-C(28)	108.61(17)
C(34)-P(2)-C(21)	104.00(16)
C(28)-P(2)-C(21)	103.62(17)
C(34)-P(2)-Au(2)	111.21(13)
C(28)-P(2)-Au(2)	115.67(11)
C(21)-P(2)-Au(2)	112.85(12)
C(1)-S(1)-Au(1)	97.69(12)
C(22)-S(2)-Au(2)	107.14(12)

Lead Thiolates ($Pb(SR_F)_2$) IR Spectra

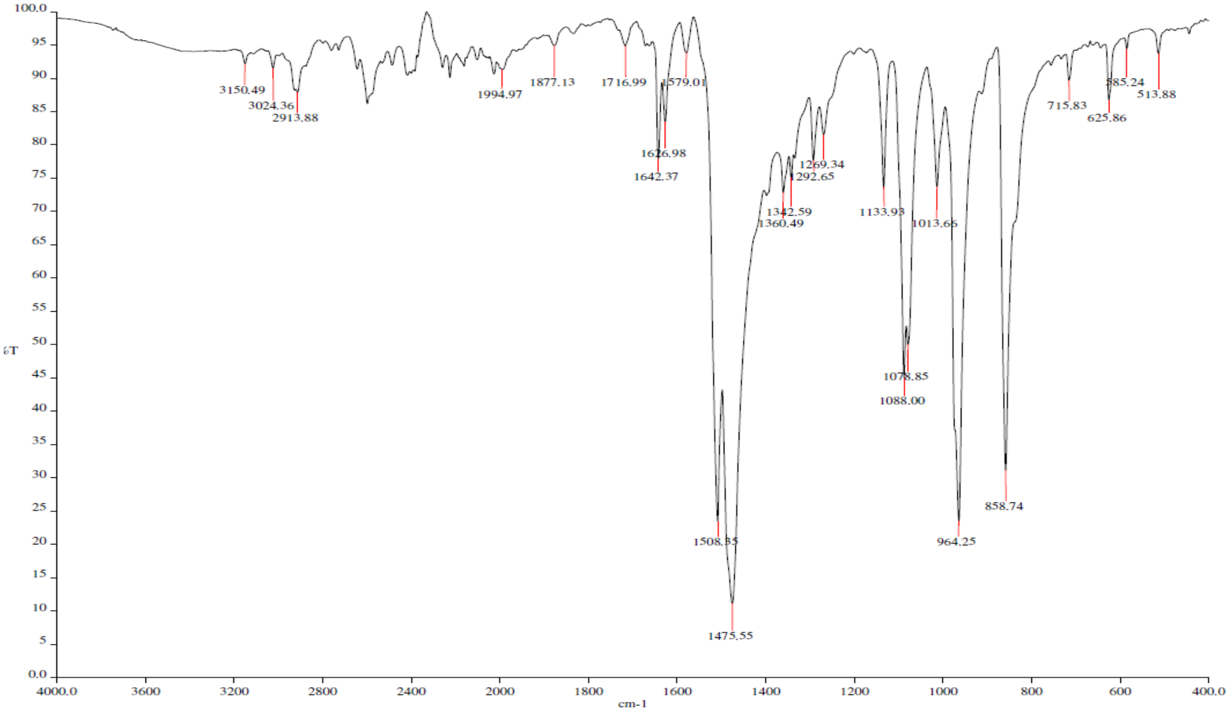

Figure S39. ATR-FTIR Spectra of $Pb(SC_6F_5)_2$

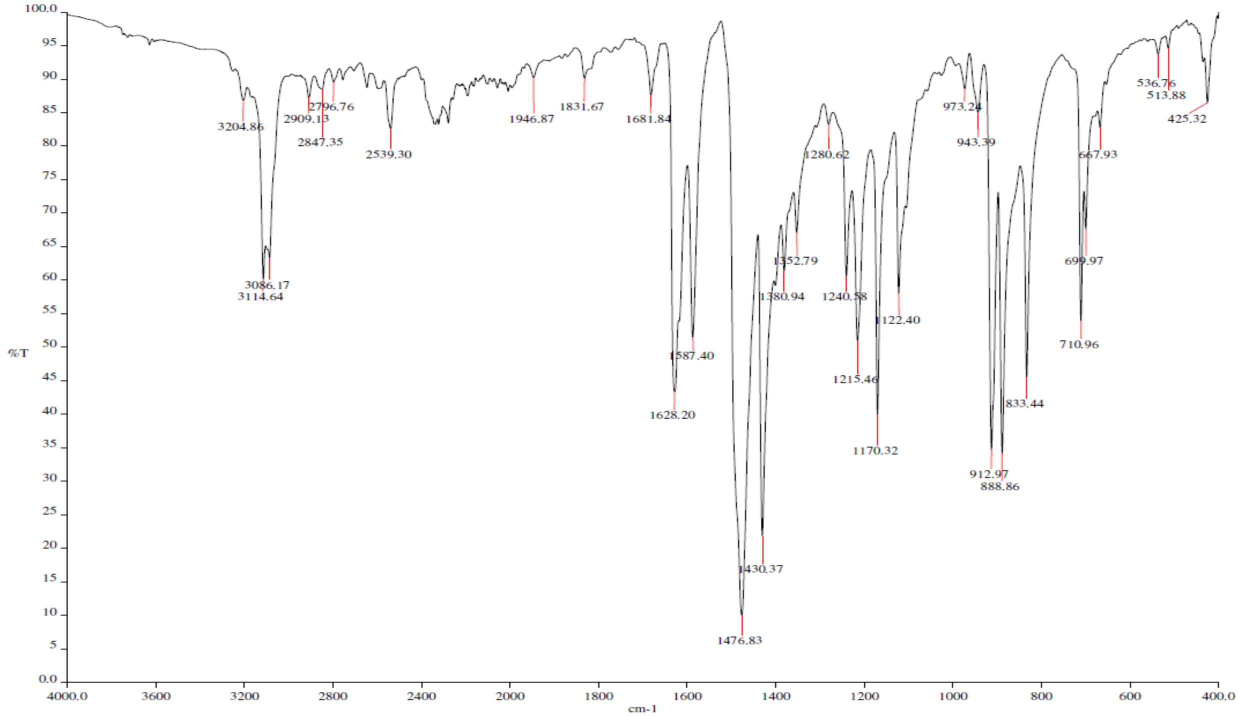

Figure S40. ATR-FTIR Spectra of $Pb(SC_6HF_4\text{-}4)_2$

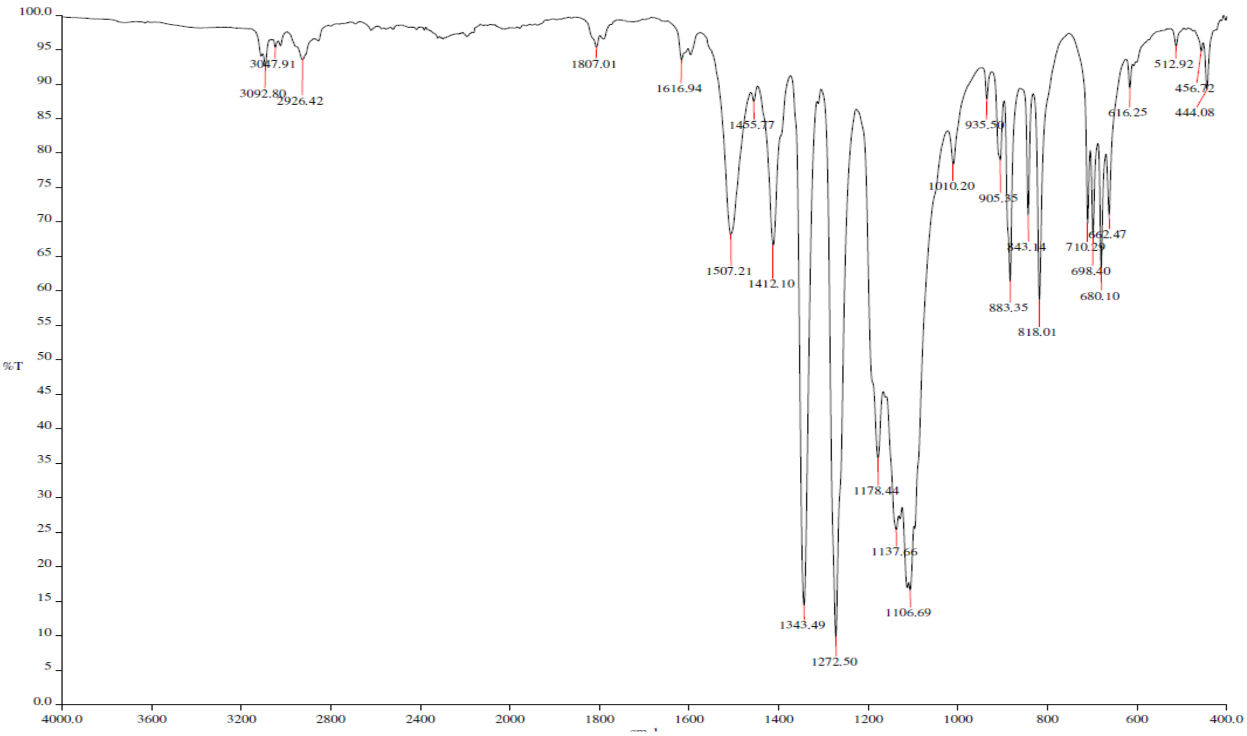

Figure S41. ATR-FTIR Spectra of $Pb(SC_6H_3(CF_3)_2\text{-}3,5)_2$

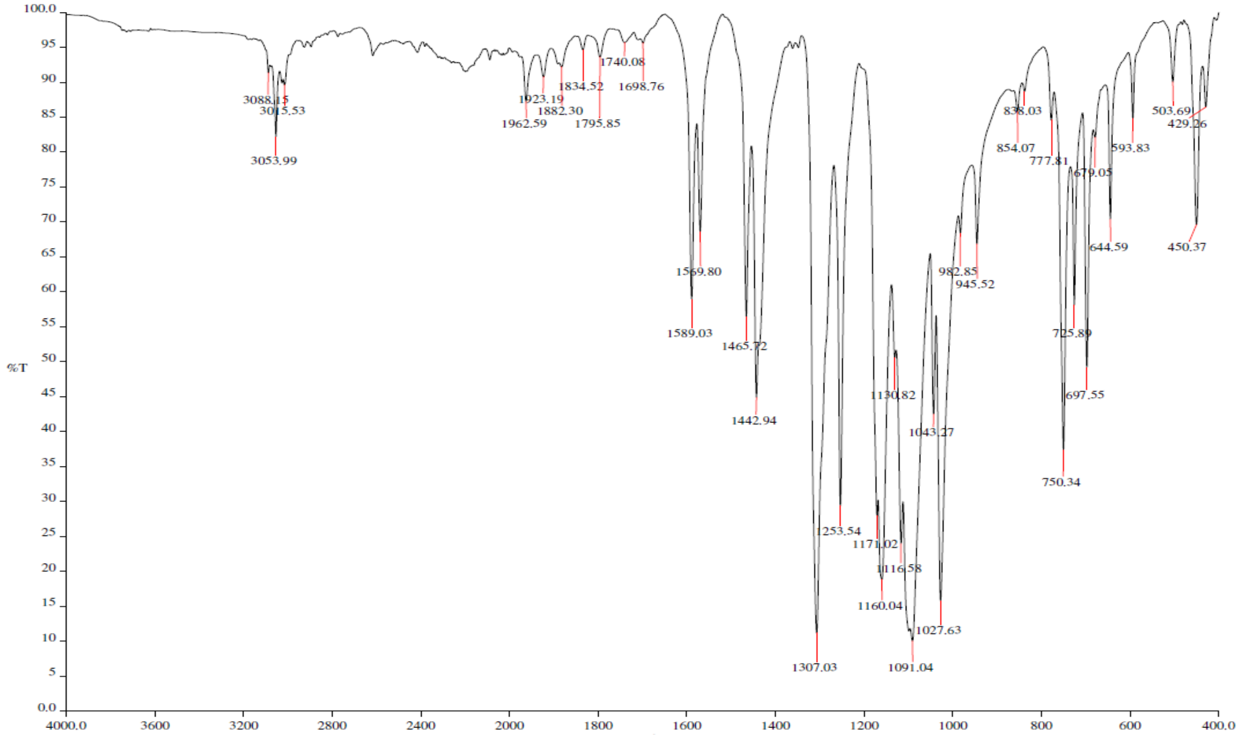

Figure S42. ATR-FTIR Spectra of $Pb(SC_6H_4(CF_3)\text{-}2)_2$

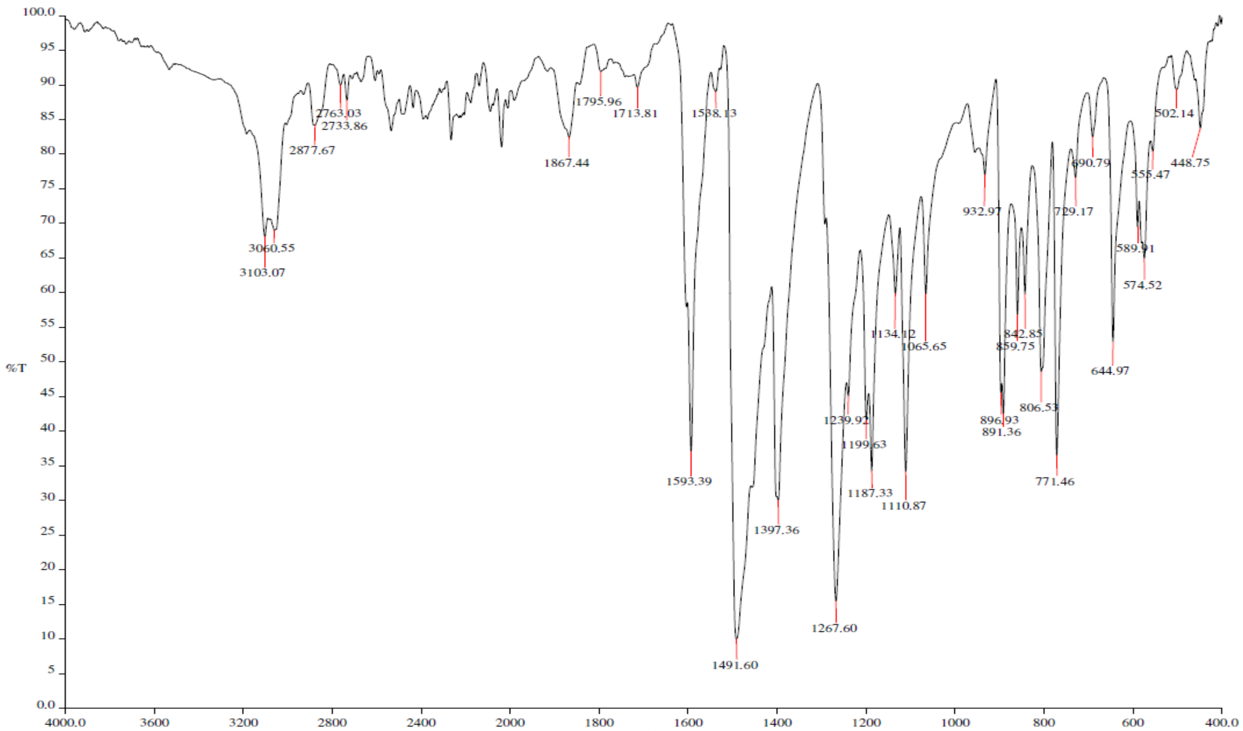

Figure S43. ATR-FTIR Spectra of Pb(SC$_6$H$_4$(CF$_3$)-4)$_2$

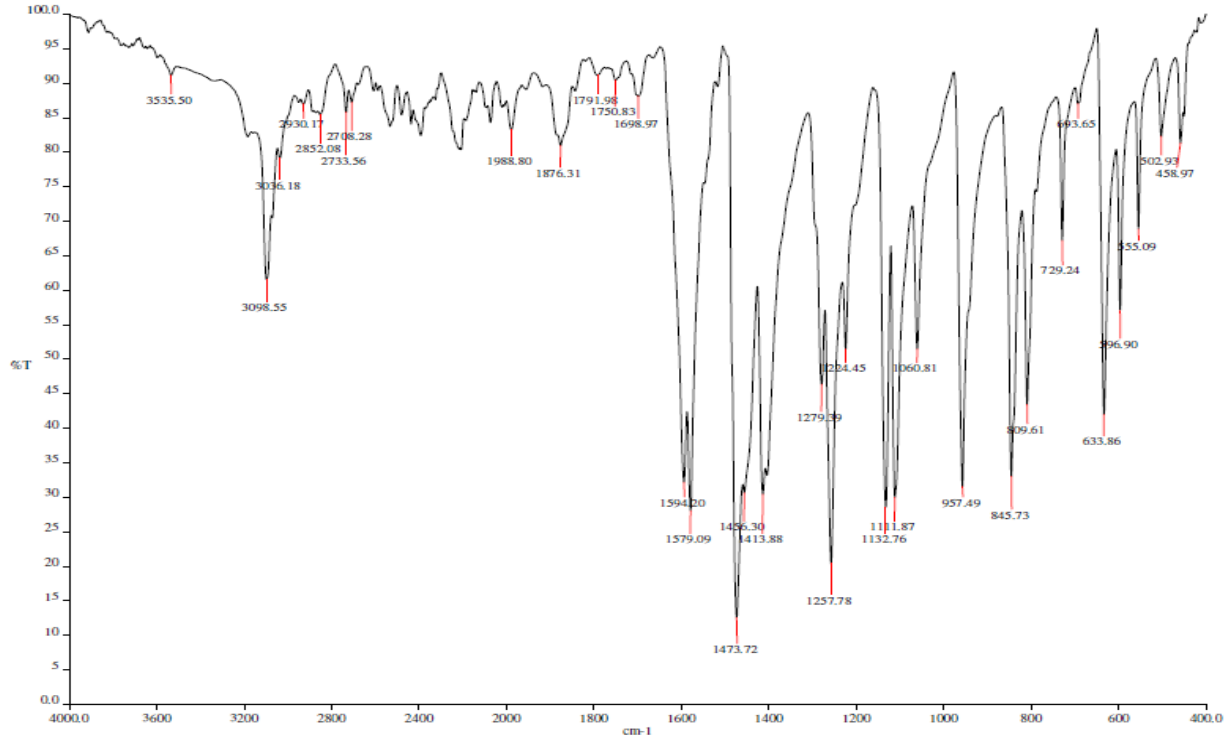

Figure S44. ATR-FTIR Spectra of Pb(SC$_6$H$_3$F$_2$-3,4)$_2$

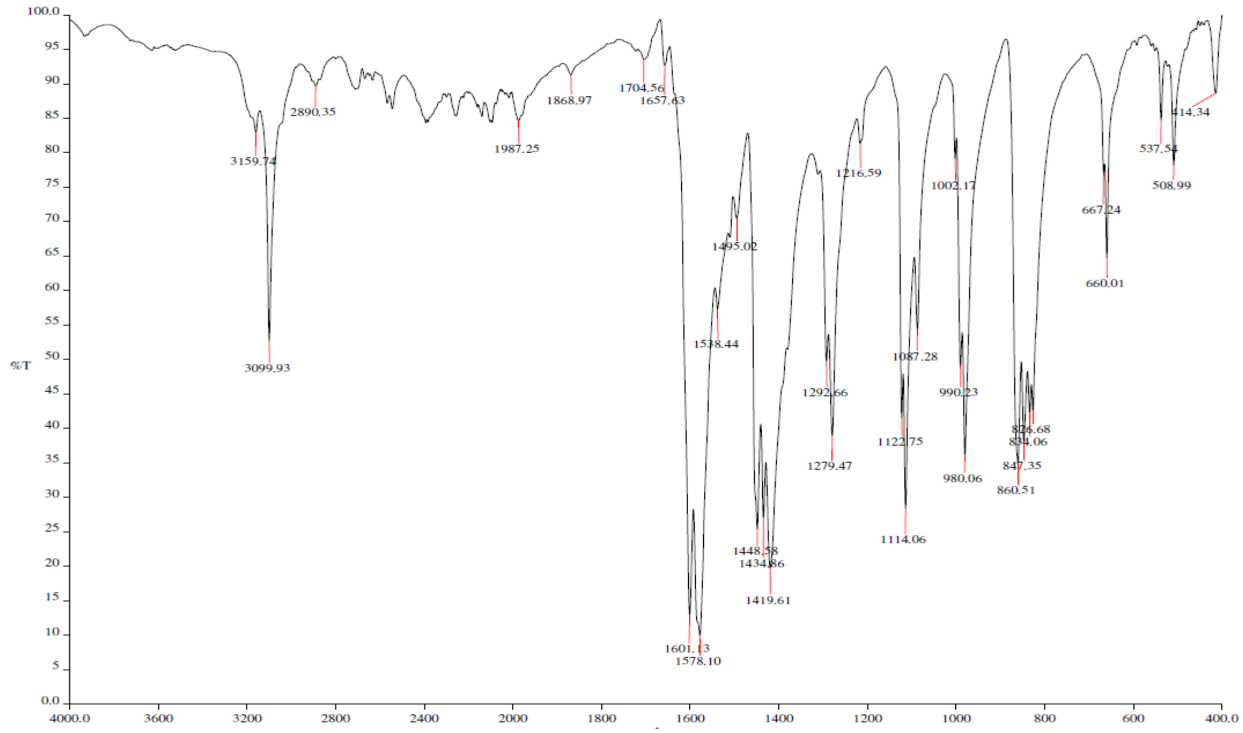

Figure S45. ATR-FTIR Spectra of $Pb(SC_6H_3F_2\text{-}3,5)_2$

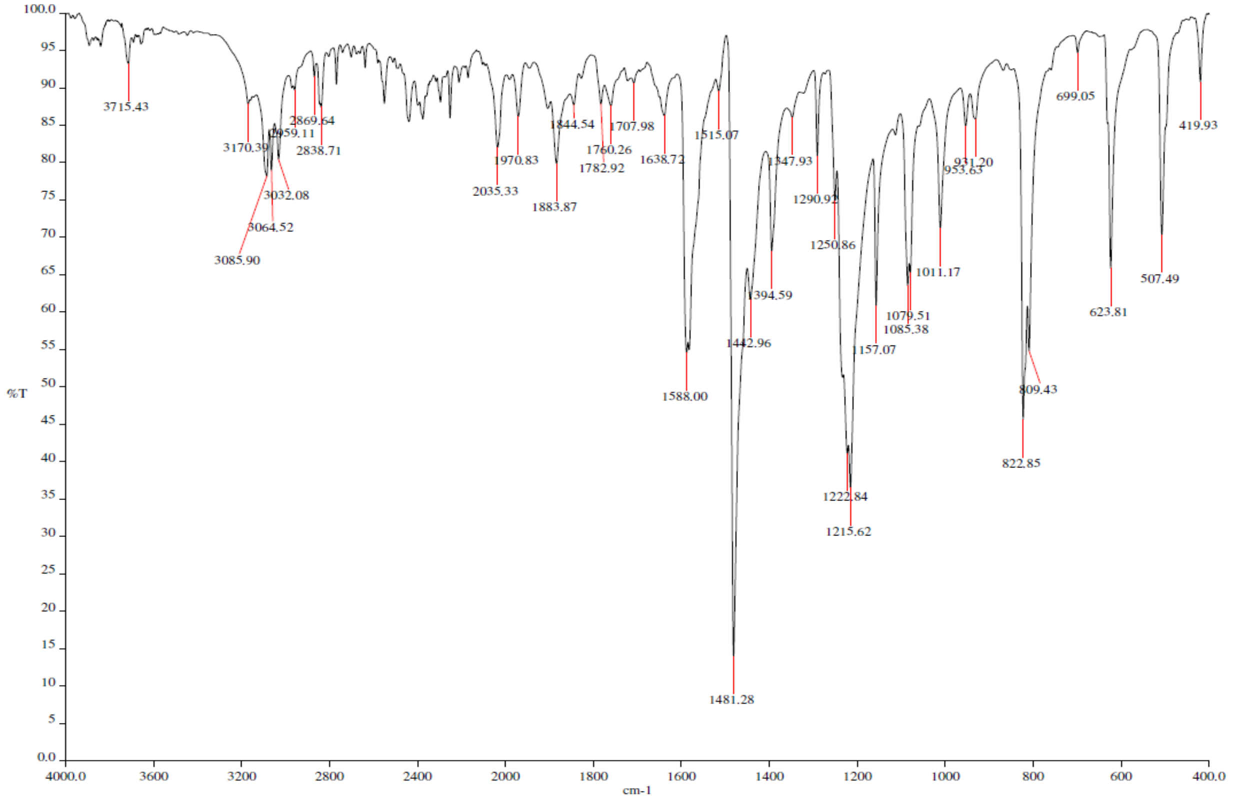

Figure S46. ATR-FTIR Spectra of $Pb(SC_6H_4F\text{-}2)_2$

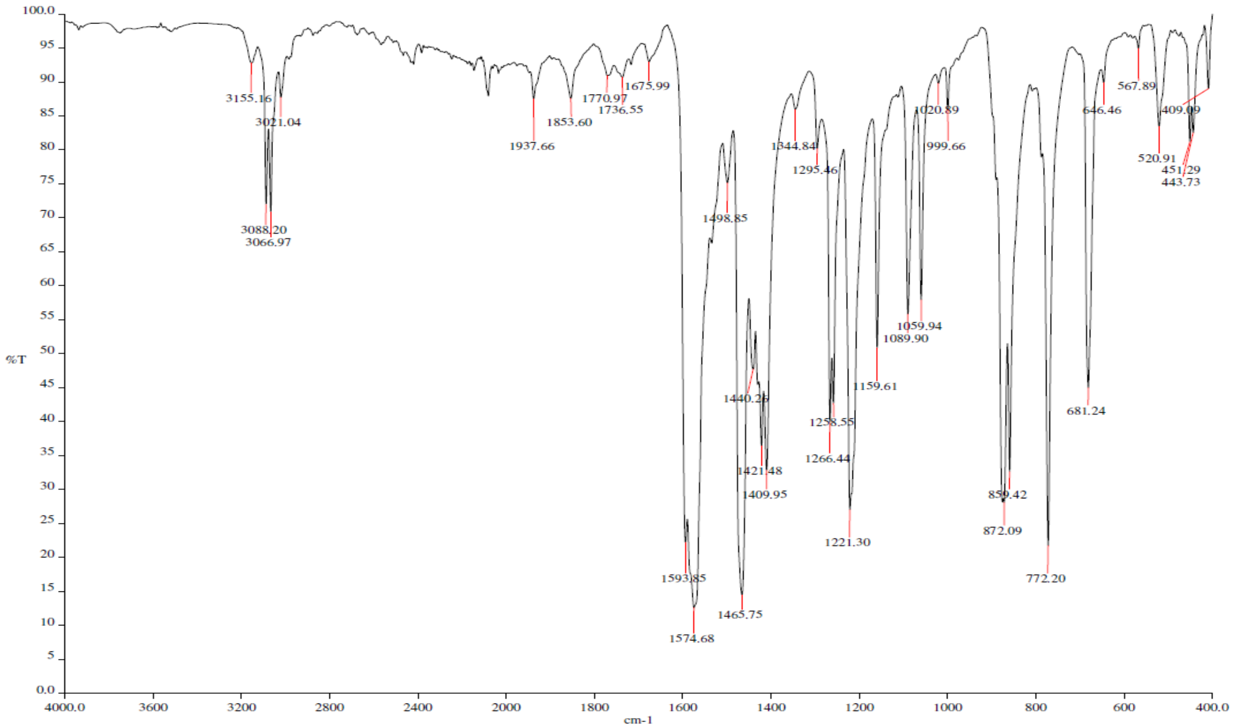

Figure S47. ATR-FTIR Spectra of Pb(SC$_6$H$_4$F-3)$_2$

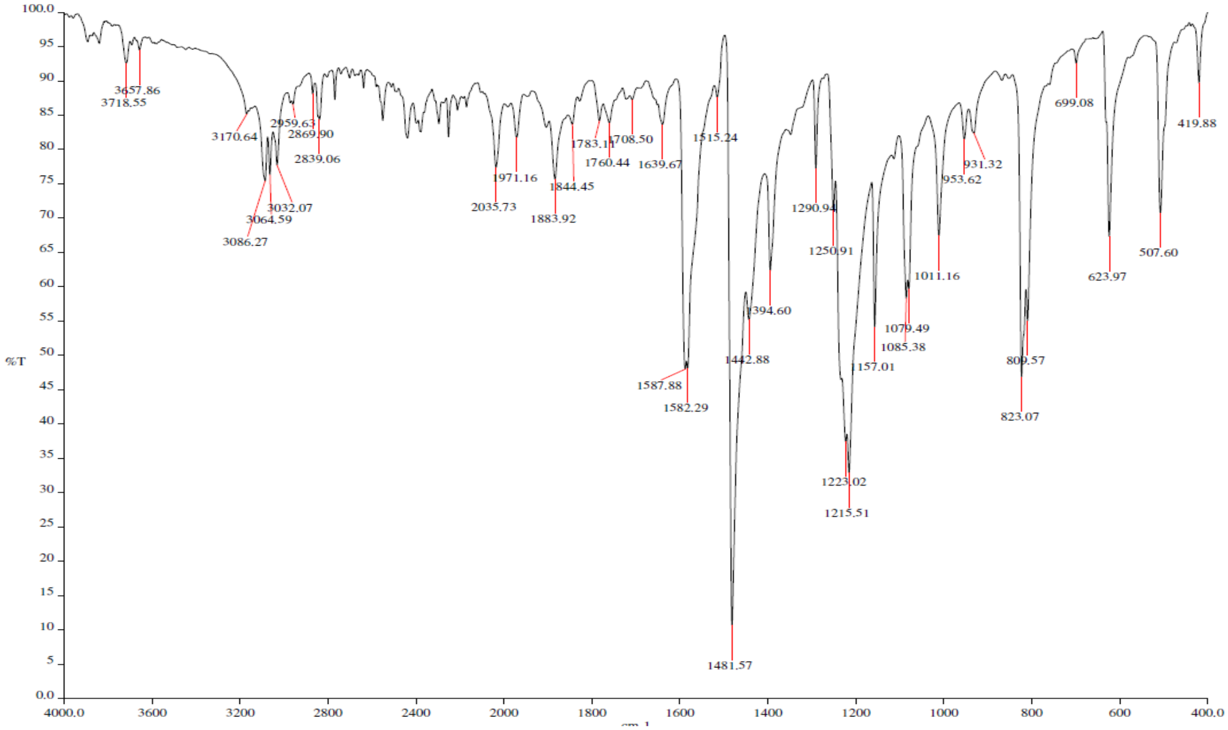

Figure S48. ATR-FTIR Spectra of Pb(SC$_6$H$_4$F-4)$_2$
